# A [CoSiH_2_] Silylene Synthon Provides Modular Access to Homo- and Heterobimetallic [Co=Si=M] (M = Co, Fe) Silicide Complexes

**DOI:** 10.1021/jacs.3c07998

**Published:** 2023-10-31

**Authors:** Rex C. Handford, T. Don Tilley

**Affiliations:** Department of Chemistry, University of California, Berkeley, Berkeley, California 94720, United States

## Abstract

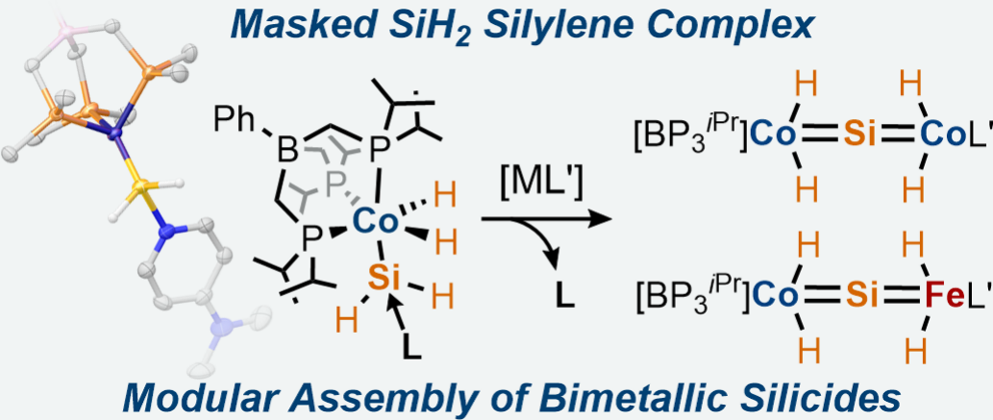

Base-stabilized [BP_3_^*i*Pr^](H)_2_CoSiH_2_(DMAP) (**1**, [BP_3_^*i*Pr^] = PhB(CH_2_P^*i*^Pr_2_)_3_^–^; DMAP = 4-dimethylaminopyridine) is a rare instance of a synthon for the simplest “parent” silylene complex (LM=SiH_2_). Complex **1** was accessed in high yields via double Si–H bond activation in SiH_4_ by [BP_3_^*i*Pr^]Co(DMAP), and in solution, it undergoes rapid exchange between bound and free DMAP by an associative mechanism (as determined by variable-temperature ^1^H NMR dynamic studies). The DMAP ligand of **1** is readily displaced by metal-based fragments that bind silicon and cleave the Si–H bonds of the SiH_2_ moiety to produce bimetallic [Co=Si=M] (M = Co, Fe) molecular silicides. Thus, treatment of **1** with 0.5 equiv of (LCo^I^)_2_(μ-N_2_) (L = a tripodal ligand) resulted in the spontaneous formation of [BP_3_^*i*Pr^](H)_2_Co=Si=Co(H)_2_L (L = [BP_2_^*t*Bu^Pz], PhB(CH_2_P^*t*^Bu_2_)_2_(pyrazolyl)^−^ (**3**); Tp″, HB(3,5-diisopropylpyrazolyl)_3_^–^ (**4**)) with the concomitant release of DMAP. The symmetrical silicide [BP_3_^*i*Pr^](H)_2_Co=Si=Co(H)_2_[BP_3_^*i*Pr^] (**5**) was prepared by treatment of a mixture of **1** and [BP_3_^*i*Pr^]Co(DMAP) with 2 equiv of Ph_3_B, which in this case is required to sequester DMAP as the elimination product Ph_3_B-DMAP. A heterobimetallic silicide, [BP_3_^*i*Pr^](H)_2_Co=Si=Fe(H)_2_[SiP_3_^*i*Pr^] (**7**; [SiP_3_^*i*Pr^] = PhSi(CH_2_P^*i*^Pr_2_)_3_), was obtained via *in situ* KC_8_ reduction of [SiP_3_^*i*Pr^]FeCl and subsequent addition of **1** and Ph_3_B. These transformations involving a metal–SiH_2_ derivative demonstrate a fundamentally new type of reactivity for silylene complexes and provide a unique synthetic method for construction of molecular silicide complexes.

## 1 Introduction

**Figure 1:**
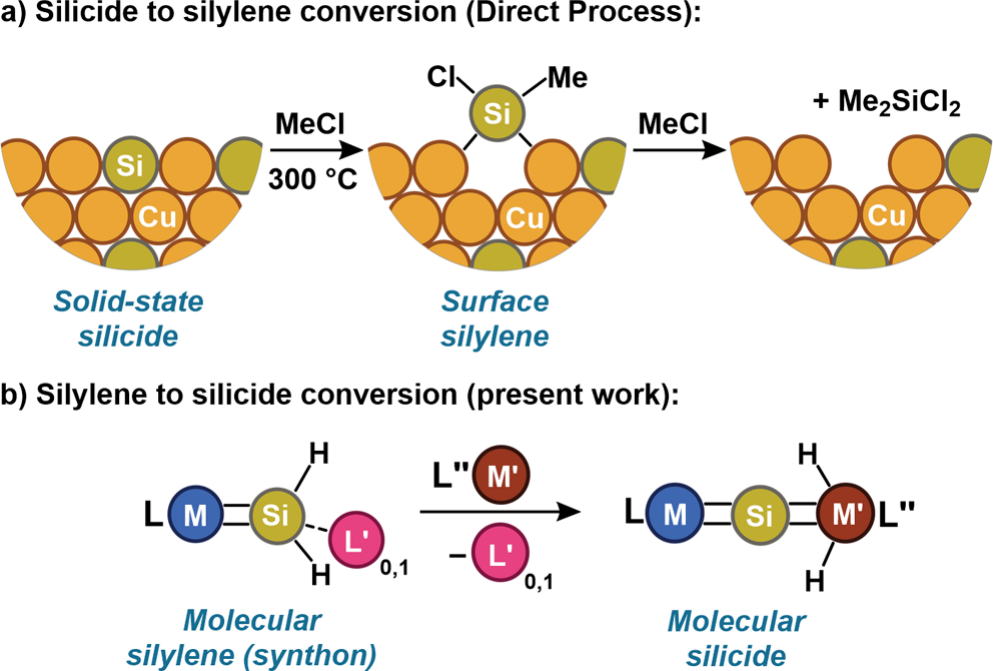
(a) Direct process reaction of copper silicide with MeCl to produce Me_2_SiCl_2_ via a silylene intermediate. (b) Conversion of a molecular SiH_2_ complex (or synthon) to a molecular silicide.

Transition-metal–silicon materials are of broad importance to industry, catalysis, and solid-state technologies.^1−6^ This is especially apparent in the direct process, which is responsible for the global production of chlorosilanes that serve as precursors to the silicone industry.^1,2^ In this process, a copper catalyst combines with silicon to form copper silicide phases (e.g., Cu_3_Si, Cu_5_Si), and reactions with MeCl on the silicide surface produce silylene species [Cu_*n*_SiClMe] that undergo further reaction to release Me_2_SiCl_2_ (Figure 1a).^1,2,7−11^ Silicide phases find additional utility in the production of silicon nanomaterials (e.g., nanowires) using silanes as a source of silicon atoms.^4^ These applications of solid-state silicides highlight the utility of both silicide (M_*x*_Si_*y*_) and silylene (LM=SiRR’) reaction centers in promoting useful transformations, and a better understanding of both types of intermediates is expected to enable new silicon-based transformations. The latter challenge can be addressed with the study of well-defined molecular models designed to reveal mechanistic details and provide starting points for catalyst design strategies.^12−15^

Molecular silicide complexes are uncommon since there are very few general, controlled synthetic routes to molecules possessing M_*x*_Si_*y*_ cores. Thus, an understanding of structure–function relationships for silicon atoms ligated to transition metals is severely lacking. Bimetallic silicides, LM=Si=ML, represent prototypical molecules of this class due to their structural simplicity and the high reactivity expected for the two-coordinate μ-silicon center. Access to such complexes was demonstrated through two primary strategies. In one case, metathetical exchange between (SIPr)SiBr_2_ (SIPr = 1,3-*bis*-(2,6-diisopropylphenyl)-4,5-dihydroimidazol-2-ylidene) and 2 equiv of [Tp*Mo(CO)_2_(PMe_3_)]^−^ (Tp* = HB(3,5-dimethylpyrazolyl)_3_^–^) produced the unsymmetrical silicide Tp*(OC)_2_Mo≡Si–Mo(CO)_2_(PMe_3_)Tp*.^16^ In addition, our laboratories reported the formation of [M=Si=M] cores from simple silanes, for example via quadruple Si–H bond activations of SiH_4_ by ([BP_2_^*t*Bu^Pz]Co)_2_(μ-N_2_), to form [BP_2_^*t*Bu^Pz](H)_2_Co=Si=Co(H)_2_[BP_2_^*t*Bu^Pz] ([BP_2_^*t*Bu^Pz] = PhB(CH_2_P^*t*^Bu_2_)_2_(pyrazolyl)^−^).^17^ The [MSiM] cores of these silicides react in unprecedented ways; for example, the μ-silicon center of Tp*(OC)_2_Mo≡Si–Mo(CO)_2_(PMe_3_)Tp* binds alkynes to generate adducts incorporating planar, tetracoordinate silicon in [(RCCR’)SiMo_2_] units.^18^ In addition, reaction of [BP_2_^*t*Bu^Pz](H)_2_Co=Si=Co(H)_2_[BP_2_^*t*Bu^Pz] with MeCl produced small quantities of functionalized silane products Me_*x*_SiH_4–*x*_ (*x* = 1–3).^17^

Despite these recent advances, molecular silicide chemistry remains largely unexplored, mainly due to the absence of synthetic methods that provide control over stoichiometry and structural features (e.g., symmetry, coordination numbers, metal identities, etc.). The results reported here demonstrate the versatile, modular assembly of complex silicide structures by way of a synthon for the simplest terminal silylene complex, the adduct [BP_3_^*i*Pr^](H)_2_CoSiH_2_(DMAP) (**1**; [BP_3_^*i*Pr^] = PhB(CH_2_P^*i*^Pr_2_)_3_^–^; DMAP = 4-dimethylaminopyridine). This complex undergoes oxidative addition of the Si···H bonds by a second metal center to afford bimetallic complexes possessing a linear [MSiM′] core (Figure 1b).

## 2 Results and Discussion

### 2.1 Access to a Base-Stabilized SiH_2_ Complex

**Scheme 1:**
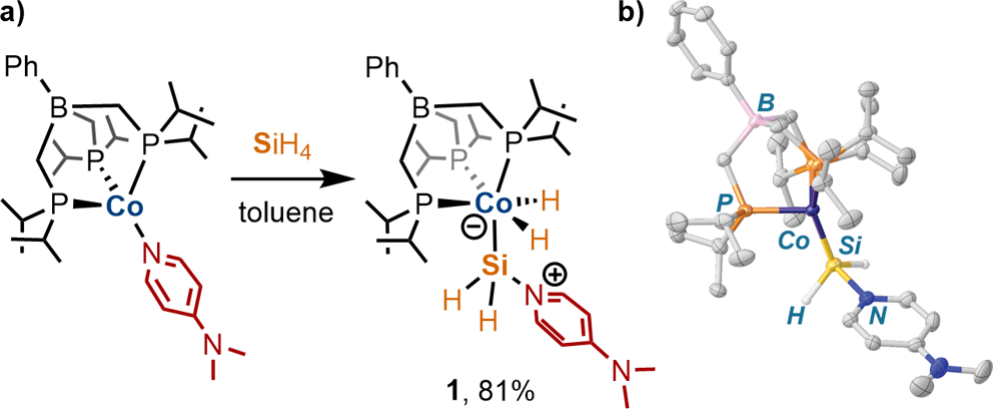
(a) Synthesis of **1**. (b) Solid-State Molecular Structure of **1** with 50% Probability Thermal Ellipsoids Drawn. Most Hydrogen Atoms Are Omitted for Clarity

The base-stabilized silylene complex [BP_3_^*i*Pr^](H)_2_CoSiH_2_(DMAP) (**1**) was obtained by reaction of [BP_3_^*i*Pr^]Co(DMAP)^19^ with SiH_4_ (1 equiv, 15% in nitrogen) in toluene (Scheme 1a), which resulted in a color change from dark brown to bright orange. Complex **1** was isolated in high yield as an analytically pure solid following crystallization by diffusion of pentane into a saturated 1,2-difluorobenzene solution at −35 °C. The solid-state molecular structure of **1** consists of two unique molecules in the asymmetric unit, one of which exhibits considerable disorder of the [BP_3_^*i*Pr^] ligand; the structure of the nondisordered complex is shown in Scheme 1b. The Co–Si bond length in this molecule (2.135(2) Å) is modestly contracted in comparison to related base-stabilized cobalt silylenes (e.g., [BP_3_^*i*Pr^](H)_2_CoSiHPh(DMAP), *d*(Co–Si) = 2.1524(8) Å;^19^ [BP_2_^*t*Bu^Pz](H)_2_CoSiHPh(DMAP), *d*(Co–Si) = 2.1428(5) Å^17^), reflecting a lower steric demand of the SiH_2_(DMAP) “silylene” unit. Complex **1** appears to be only the second example of a LMSiH_2_(L′) complex, since Radius et al. reported that reaction of *trans*-Mes_2_(Me_2_Im)_2_Fe with 2 equiv of PhSiH_3_ produced (Me_2_Im)_2_Fe[SiH_2_(Me_2_Im)][μ-(H)_3_SiHPh_2_] via PhSiH_3_ redistribution (Me_2_Im = 1,3-dimethylimidazol-2-ylidene).^20^

In a benzene-*d*_6_ solution, **1** exhibits dynamic behavior as evidenced by its ^1^H and ^31^P{^1^H} NMR spectra. At 292 K, the Co–*H* and Si–*H* hydrogen nuclei resonate at δ −15.44 ppm (br s, 2H) and 6.36 ppm (*pseudo*-q, *J* = 7.5 Hz, ^1^*J*_SiH_ = 159 Hz, 2H), respectively. The ^31^P{^1^H} NMR spectrum displays a single broad resonance at δ 62.6 ppm, indicating interconversion of phosphorus environments in the [BP_3_^*i*Pr^] ligand. The silicon nucleus of **1** resonates at δ 33 ppm according to a ^29^Si–^1^H HMBC NMR spectrum. Variable-temperature (VT) NMR spectroscopic studies of **1** in a toluene-*d*_8_ solution show that upon cooling, the ^31^P{^1^H} NMR resonance decoalesces, and at 237 K, it is fully resolved into two resonances (1:2 ratio) at δ 61.1 and 62.9 ppm. The solution ^29^Si chemical shift of complex **1**, coupled with the tetrahedral coordination geometry of silicon in the solid-state structure, indicates that there is minimal double-bond character within the Co–Si linkage, a feature that is consistent with other base-stabilized silylene species.^13^

The lability of the coordinated DMAP donor in **1** was of interest as an indication of possible reactivity modes for the SiH_2_(DMAP) ligand. For context, note that previous VT-NMR studies of the base-stabilized silylene complex [Cp*(Me_3_P)_2_RuSiPh_2_(NCMe)]^+^ (Cp* = η^5^-C_5_Me_5_) demonstrated that exchange of bound and free NCMe occurs by a dissociative mechanism involving a base-free [Ru=SiPh_2_] silylene intermediate.^21^ Indeed, the ^1^H NMR spectrum of a mixture containing **1** and 1 equiv of added DMAP (292 K, toluene-*d*_8_) contains broadened resonances for the uncoordinated DMAP ligand, indicating a facile exchange process. Upon cooling, the resonances corresponding to coordinated and “free” DMAP sharpen. Line-shape analyses of spectra from 215 to 256 K provided an Eyring plot, from which Δ*H*^‡^ (1.8 ± 0.2 kcal^–1^ mol^–1^) and Δ*S*^‡^ (−40.0 ± 0.7 J mol^–1^ K^–1^) parameters were extracted (Δ*G*_(298 K)_^‡^ = 13.7 ± 0.3 kcal^–1^ mol^–1^). The large negative entropy of activation points to an associative mechanism for DMAP exchange in **1**, in contrast to the dissociative mechanism identified for [Cp*(Me_3_P)_2_RuSiPh_2_(NCMe)]^+^.

**Scheme 2:**
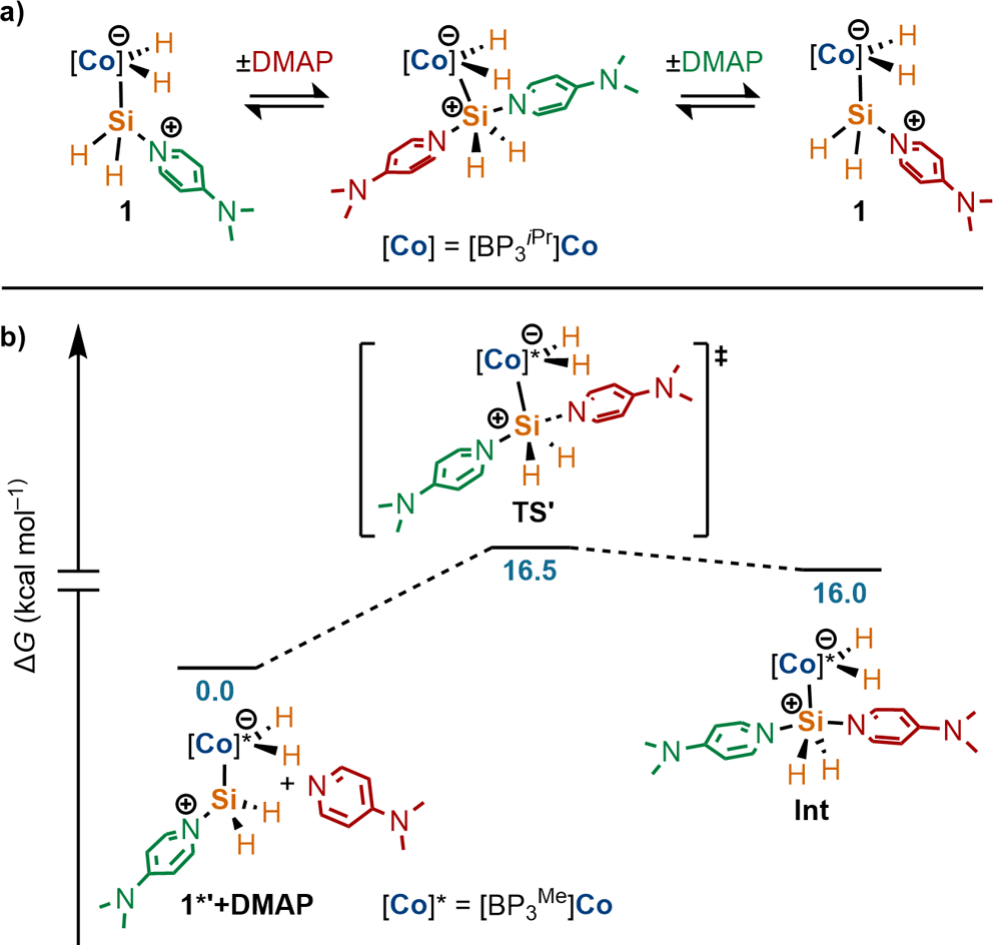
(a) Proposed Exchange Pathway for the DMAP Ligand of **1**. (b) Reaction Coordinate Diagram for the DFT-Computed DMAP Exchange Mechanism of 1*’. The Stereochemistry of 1*’ Is as Presented in the Scheme

A reasonable pathway for DMAP exchange in **1** involves the hypercoordinate intermediate [BP_3_^*i*Pr^](H)_2_CoSiH_2_(DMAP)_2_ (Scheme 2a). This exchange pathway was investigated by density functional theory (DFT) studies (ωB97X-D3/def2-TZVP(Co,Si,P),def2-SVP(C,H,B)// CPCM(toluene)) for the model complex [BP_3_^Me^](H)_2_CoSiH_2_(DMAP) ([BP_3_^Me^] = PhB(CH_2_PMe_2_)_3_^–^; 1*). The geometry-optimized structure of 1* was obtained following the adaptation of the crystallographic atomic coordinates of **1**. A potential energy scan of rotation about the Co–Si bond identified a rotamer, 1*’ (Scheme 2b), which is further stabilized by −1.1 kcal^–1^ mol^–1^ compared to 1*; the rotamers 1* and 1*’ are separated by a small rotational barrier of 5.0 kcal^–1^ mol^–1^ (Table S4). Exchange of DMAP with either 1* or 1*’ is expected to involve the *bis*-DMAP adduct [BP_3_^Me^](H)_2_CoSiH_2_(DMAP)_2_ (**Int**). A nudged-elastic band transition-state (NEB-TS)^22^ search located transition-state structures for DMAP addition to 1* or 1*’ to form **Int** (Scheme 2b). The calculated thermochemical parameters (relative to 1*’ + DMAP = 0 kcal^–1^ mol^–1^) of Δ*H*^‡^ (4.4 kcal^–1^ mol^–1^), Δ*S*^‡^ (−48.8 J mol^–1^ K^–1^), and Δ*G*_(298 K)_^‡^ (16.5 kcal^–1^ mol^–1^) are consistent with those derived from the Eyring plot, lending support to the proposed associative mechanism.

**Scheme 3:**
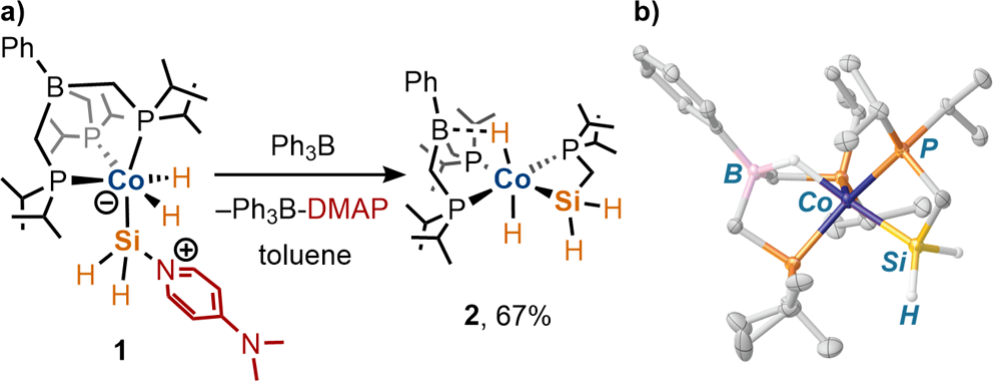
(a) Synthesis of **2**. (b) Solid-State Molecular Structure of **2** with 50% Probability Thermal Ellipsoids. Most Hydrogen Atoms Are Omitted for Clarity

To examine whether DMAP could be removed from **1** by chemical abstraction, the complex was treated with 1 equiv of Ph_3_B in a toluene solution. A darkening of the solution’s color was immediately apparent, and ^1^H and ^31^P{^1^H} NMR spectra of the reaction mixture indicated clean conversion to Ph_3_B–DMAP and a new diamagnetic species, which was identified by X-ray crystallography and solution-state multinuclear NMR spectroscopy as [PhB(CH_2_P^*i*^Pr_2_)_2_](H)_2_Co[κ^2^-*Si*,*P*-H_2_SiCH_2_P^*i*^Pr_2_] (**2**; Scheme 3). Complex **2** results from coupling of a [BP_3_^*i*Pr^] ligand –CH_2_P^*i*^Pr_2_ side arm with the SiH_2_ moiety, suggesting the possible intermediacy of the base-free species [BP_3_^*i*Pr^](H)_2_CoSiH_2_. However, the rapid course of the reaction (complete in <5 min) indicates that any such base-free silylene cannot be readily isolated under these conditions. Nevertheless, addition of Ph_3_B to **1** represents a potential strategy for activating the [SiH_2_] unit toward further reactivity in the presence of a suitable substrate (*vide infra*).

### 2.2 Homo- and Heterobimetallic Silicides

Possible transformations of LMSiH_2_ and LMSiH_2_(L′) species involve further activation of the Si–H bonds by an exogenous metal reagent to afford silicide complexes with a [MSiM′] core. Since donor-free [BP_3_^*i*Pr^](H)_2_CoSiH_2_ has proven synthetically elusive, complex **1** was employed as a synthon in reactions with a second metal center designed to promote Si–H bond activation, DMAP displacement, and Si–M′ bond formations. As described below, this approach has led to the formation of bimetallic silicide complexes [BP_3_^*i*Pr^](H)_2_Co=Si=M′(H)_2_L (M′ = Co, Fe; L = tripodal ligand).

**Scheme 4:**
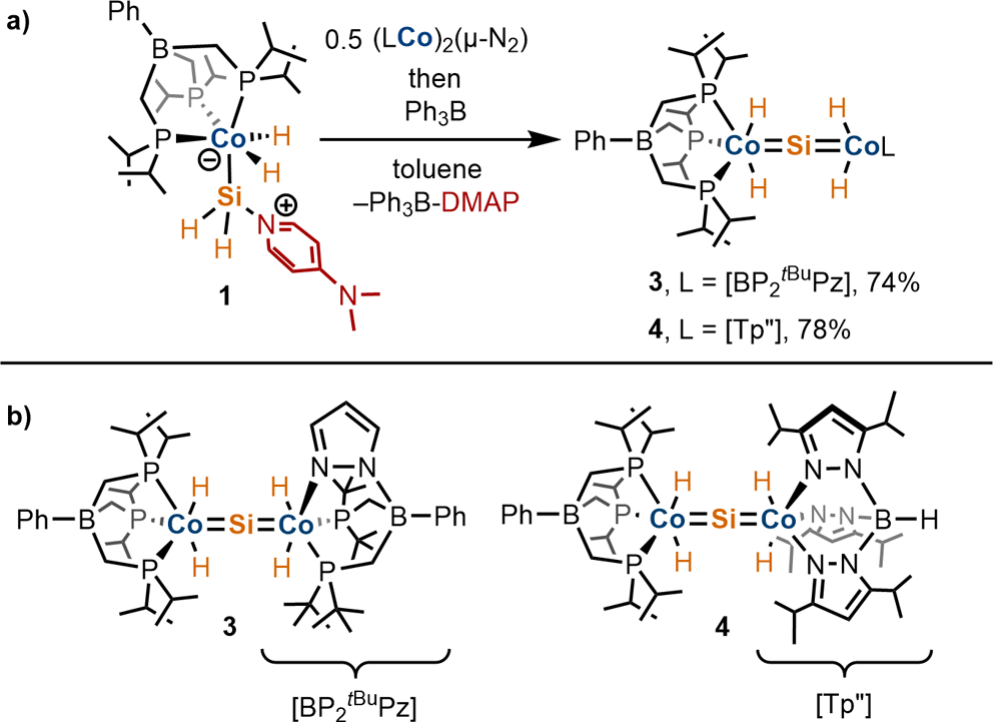
(a) Reaction of **1** with (LCo)_2_(μ-N_2_) to Form Silicides **3** and **4** (L = [BP_2_^tBu^Pz], Tp″). Spontaneously Released DMAP Is Sequestered by Ph_3_B. (b) Depiction of Molecular Structures of **3** and **4**

Previous work from this laboratory demonstrated that ([BP_2_^*t*Bu^Pz]Co)_2_(μ-N_2_) serves as a source of [BP_2_^*t*Bu^Pz]Co^I^, which readily activates the Si–H bonds of SiH_4_ to generate the silicide [BP_2_^*t*Bu^Pz](H)_2_Co=Si=Co(H)_2_[BP_2_^*t*Bu^Pz].^17^ It therefore seemed that ([BP_2_^*t*Bu^Pz]Co)_2_(μ-N_2_), serving as a source of [BP_2_^*t*Bu^Pz]Co^I^, might react with **1** to generate the unsymmetrical silicide [BP_3_^*i*Pr^](H)_2_Co=Si=Co(H)_2_[BP_2_^*t*Bu^Pz] (**3**). Indeed, the addition of a clear red-brown toluene solution of ([BP_2_^*t*Bu^Pz]Co)_2_(μ-N_2_) to a toluene solution of **1** resulted in a rapid color change to deep blue. Multinuclear ^1^H and ^31^P{^1^H} NMR spectroscopic analyses of the crude reaction mixture indicated full consumption of the starting materials to form a new diamagnetic species whose features are consistent with **3**, in high (93%) yield, according to integration against an internal standard of (Me_3_Si)_2_O (Scheme 4). Introduction of Ph_3_B to the reaction mixture resulted in near-quantitative capture of the released DMAP to form Ph_3_B–DMAP (97%, via ^1^H NMR spectroscopy). The adduct Ph_3_B–DMAP was conveniently removed from the reaction mixture via crystallization by diffusion of pentane into a dilute tetrahydrofuran (THF) solution at −35 °C. Complex **3**, enriched in the supernatant solution, was crystallized by diffusion of (Me_3_Si)_2_O into a saturated THF solution cooled to −35 °C and was isolated in an analytically pure form in good yield (74%).

An additional unsymmetrical dicobalt silicide, [BP_3_^*i*Pr^](H)_2_Co=Si=Co(H)_2_Tp″ (**4**, Tp″ = HB(3,5-diisopropylpyrazolyl)_3_^–^), was generated in an analogous fashion by treatment of **1** with 0.5 equiv of (Tp″Co)_2_(μ-N_2_).^17^ A color change of the reaction mixture from dark brown to blue was observed upon addition of (Tp″Co)_2_(μ-N_2_), and multinuclear NMR spectroscopy (^1^H, ^31^P{^1^H}) indicated formation of a single diamagnetic species (in addition to DMAP) possessing features consistent with [BP_3_^*i*Pr^](H)_2_Co=Si=Co(H)_2_Tp″ (**4**; Scheme 4). Complex **4** was isolated as an analytically pure dark-blue powder, following precipitation from a concentrated diethyl ether solution. However, **4** has yet to be isolated in a crystalline form suitable for X-ray diffraction studies.

The isolation of **4** is surprising as direct treatment of (Tp″Co)_2_(μ-N_2_) with SiH_4_ or PhSiH_3_ has been shown to result in mixtures of the μ-hydrides (Tp″Co)_2_(μ-H)_*x*_ (*x* = 1, 2).^17^ This hydrogen-atom abstraction was favored over silicide formation, possibly due to the inability of the Tp″ ligands to enforce sufficiently long Co···Co separations to stabilize a linear [CoSiCo] core.^17^ Thus, the accessibility of **4** is likely related to beneficial steric properties for the [BP_3_^*i*Pr^] ligand.


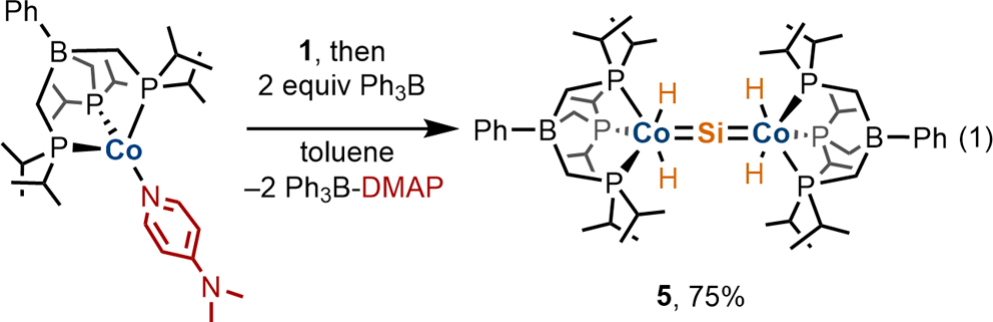
1

Using this synthetic approach, complex **1** was converted to the symmetrical homobimetallic silicide [BP_3_^*i*Pr^](H)_2_Co=Si=Co(H)_2_[BP_3_^*i*Pr^] (**5**; eq 1). Complex **5** has remained elusive despite previous attempts to access this structure by treatment of Na(THF)_6_([BP_3_^*i*Pr^]CoI) with 0.5 equiv of SiH_4_.^19^ In that case, the silicide [BP_3_^*i*Pr^](H)_2_Co=Si=Co(H)_2_(SiH_3_)[(^*i*^Pr_2_PCH_2_)_2_BPh] was generated via a process involving the degradation of a [BP_3_^*i*Pr^] ligand. With complex **1** in hand, **5** was synthesized by addition of 2 equiv of Ph_3_B to a solution containing **1** and [BP_3_^*i*Pr^]Co(DMAP) (eq 1). Monitoring the reaction by ^1^H NMR spectroscopy showed that prior to the addition of Ph_3_B, no reaction of the starting complexes was evident. Addition of Ph_3_B was accompanied by a rapid color change from dark orange-brown to red, and ^1^H and ^31^P{^1^H} NMR spectroscopic analyses of the crude reaction mixture indicated that **5** and Ph_3_B—DMAP were generated as the exclusive products in quantitative yields. Complex **5** was isolated as an analytically pure crystalline solid by crystallization from a concentrated neat THF solution.

**Scheme 5:**
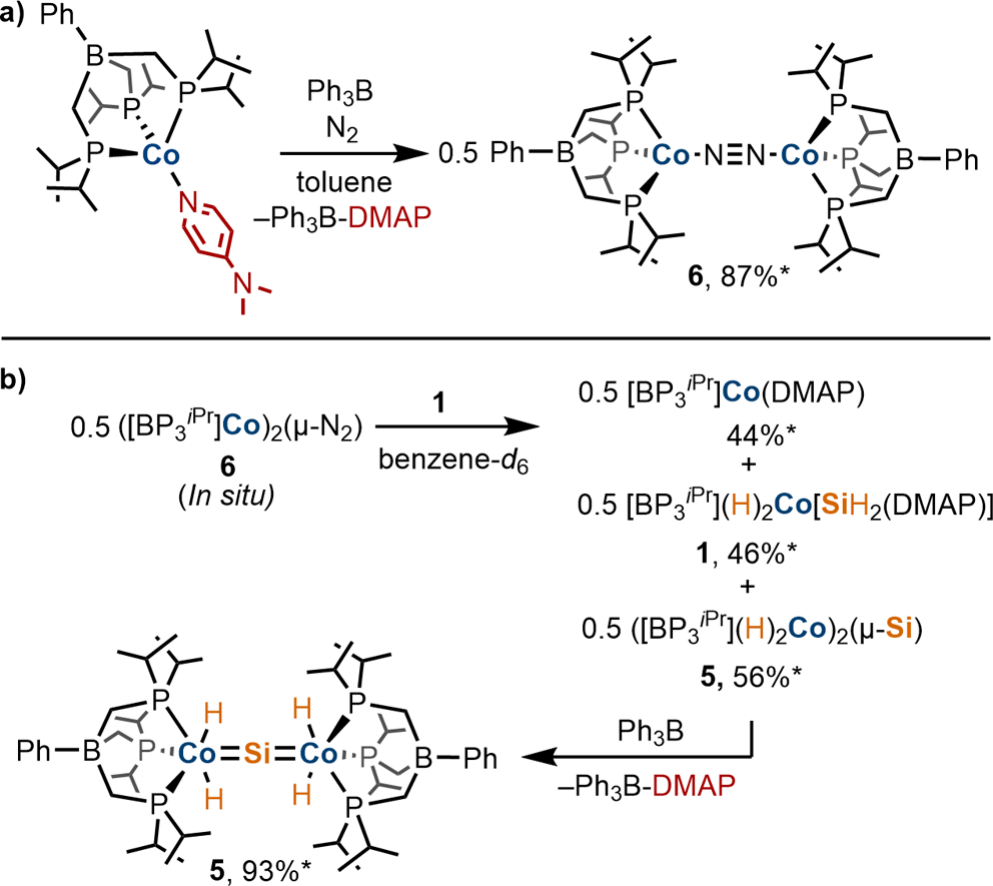
(a) *In Situ* Generation of **6** via DMAP Abstraction from [BP_3_^iPr^]Co(DMAP). (b) Reaction of **6** with **1** to Generate a Mixture Containing [BP_3_^iPr^]Co(DMAP), **1**, and **5**, and Subsequent Convergence of the Reaction Mixture to **5** upon Addition of Ph_3_B. *Yield Determined by Integration of the ^1^H NMR Spectrum against an Internal Standard

In contrast to the reactions that produce **3** and **4**, the reaction of **1** and [BP_3_^*i*Pr^]Co(DMAP) to generate **5** did not spontaneously liberate DMAP. Thus, coordinatively labile starting materials are not strictly necessary for silicide formation, and Ph_3_B is a potential trigger for such coupling processes. By analogy with the reactions depicted in Scheme 4, another potentially suitable starting material for the preparation of **5** seemed to be the μ-dinitrogen complex ([BP_3_^*i*Pr^]Co)_2_(μ-N_2_) (**6**). Complex **6** was independently generated by the addition of Ph_3_B to [BP_3_^*i*Pr^]Co(DMAP) in a benzene-*d*_6_ solution in high yield (Scheme 5a). Complex **6** is thermally sensitive and decomposes to an intractable brown-colored material upon exposure to vacuum although small quantities of crystalline **6** were obtained following storage of a (Me_3_Si)_2_O/benzene-*d*_6_ mixture at −35 °C, allowing for structural determination by single-crystal X-ray diffraction analysis (see the Supporting Information page S16).

Surprisingly, ^1^H and ^31^P{^1^H} NMR spectroscopic monitoring of the reaction of *in situ* generated **6** (0.5 equiv) and **1** (1 equiv) revealed the formation of a mixture containing 0.5 equiv of [BP_3_^*i*Pr^]Co(DMAP), 0.5 equiv of **1**, and 0.5 equiv of **5** rather than full conversion to **5** (Scheme 5b). Further addition of Ph_3_B (1 equiv) resulted in the near-quantitative formation of **5** and Ph_3_B—DMAP (by integration of the ^1^H NMR spectrum against an internal standard). These observations are consistent with a dynamic system in which **6** serves both to activate Si–H bonds in **1** and to abstract DMAP. The latter event results in sequestration of 0.5 equiv of [BP_3_^*i*Pr^]Co^I^ units supplied by **6** as [BP_3_^*i*Pr^]Co(DMAP), necessitating an additional equiv of Ph_3_B for complete conversion to **5**. The differing behavior of **6** toward **1** in comparison to the congeneric [BP_2_^*t*Bu^Pz]- and Tp″-complexes indicates that DMAP is the most strongly bound to the [BP_3_^*i*Pr^]Co^I^ fragment.

**Figure 2:**
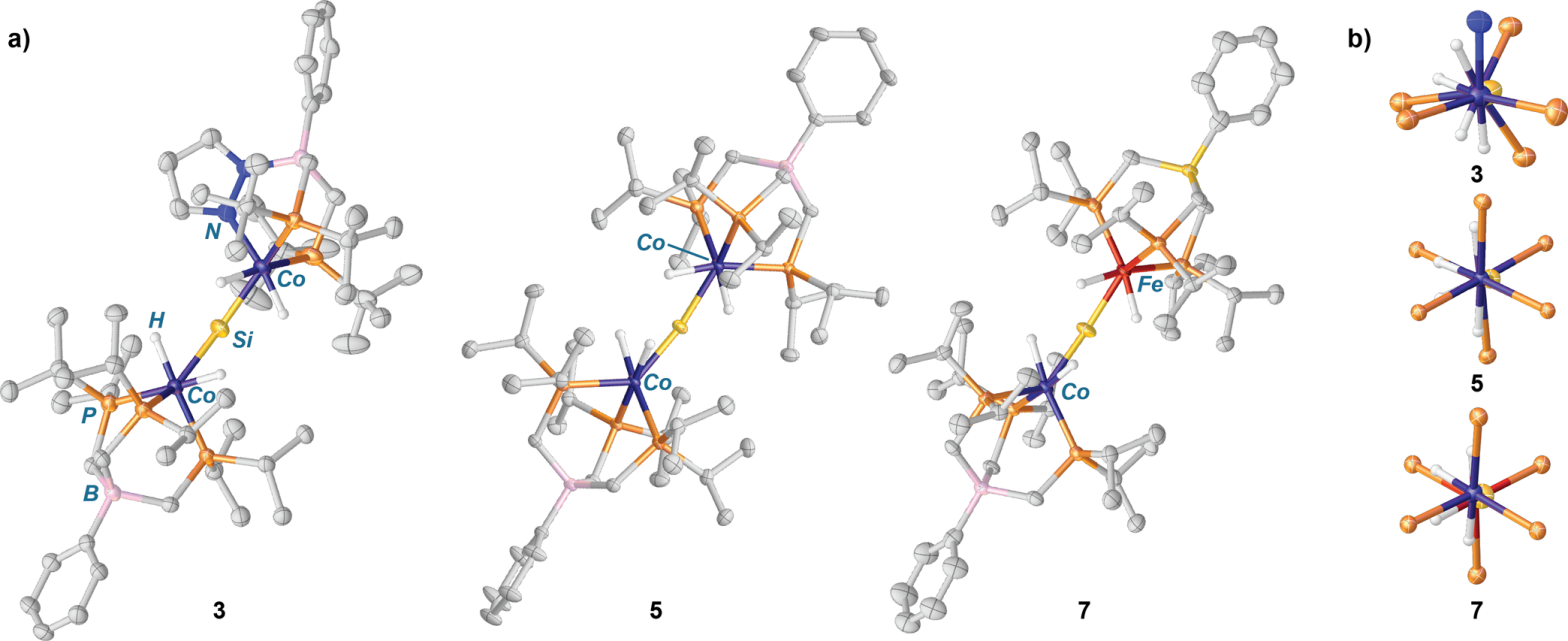
(a) Solid-sate molecular structures of silicide molecules **3**, **5**, and **7** with 50% probability thermal ellipsoids drawn. Most hydrogen atoms are omitted for clarity. (b) Views of **3**, **5**, and **7** along the Co···M′ (M′ = Co, Fe) axes, illustrating nontetrahedral arrays of hydride ligands about the μ-silicon atoms.


The utility of **1** for preparation of *hetero*bimetallic silicides (i.e., [MSiM′]) was also evaluated. For this purpose, the Fe^I^ starting material [SiP_3_^*i*Pr^]FeCl ([SiP_3_^*i*Pr^] = PhSi(CH_2_P^*i*^Pr_2_)_3_), previously reported by this laboratory,^23^ was employed. It seemed that the 14-electron [SiP_3_^*i*Pr^]Fe^0^ fragment, which is electronically similar to [BP_3_^*i*Pr^]Co^I^, would be well suited for activation of the Si–H bonds of **1**. Treatment of [SiP_3_^*i*Pr^]FeCl with 1 equiv of KC_8_ in a THF solution was accompanied by a color change from clear green to dark red. Although the dark-red intermediate has yet to be characterized, closely related tridentate *tris*-phosphine iron(0) systems have been described and isolated as LFe(N_2_)_2_ adducts.^24,25^ No conversion to a silicide was apparent upon addition of **1** to the dark-red solution, according to ^1^H and ^31^P{^1^H} NMR spectroscopies. However, the addition of Ph_3_B to the reaction mixture resulted in rapid conversion to a new diamagnetic species, formulated as [BP_3_^*i*Pr^](H)_2_Co=Si=Fe(H)_2_[SiP_3_^*i*Pr^] (**7**; eq 2). Complex **7** was authenticated by single-crystal X-ray diffraction analysis (Figure 2a, right), elemental analysis, and multinuclear NMR spectroscopy (*vide infra*).

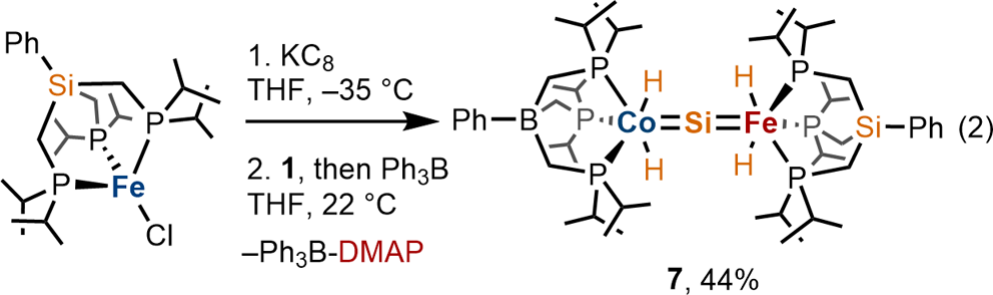
2

### 2.3 Spectroscopic and Structural Properties of Silicides

The ^1^H, ^31^P{^1^H}, and ^29^Si{^1^H} DEPT NMR spectra of **3**, **4**, **5**, and **7** display features consistent with silicide structures of this class.^17,19^ Owing to its high molecular symmetry, complex **5** displays the most straightforward NMR spectroscopic features. In benzene-*d*_6_ solution, the hydride ligands of **5** appear as a broad singlet (δ −13.16 ppm, 4H) in the ^1^H NMR spectrum, and only one line is displayed in the ^31^P{^1^H} NMR spectrum (δ 65.5 ppm). The NMR spectroscopic features of **3** resemble a superposition of those for **5** and [BP_2_^*t*Bu^Pz](H)_2_Co=Si=Co(H)_2_[BP_2_^*t*Bu^Pz], reflecting the incorporation of [BP_3_^*i*Pr^](H)_2_Co and [BP_2_^*t*Bu^Pz](H)_2_Co fragments into **3**.^17^ The hydride resonances of **3** appear at δ −13.02 ppm ([BP_3_^*i*Pr^](*H*)_2_Co, 2H), −24.58 ppm ([BP_2_^*t*Bu^Pz](*H*)_2_Co, 1H), and −9.91 ppm ([BP_2_^*t*Bu^Pz](*H*)_2_Co, 1H). The “blended” character of **3** is also apparent in the ^31^P{^1^H} NMR spectrum, which displays resonances at δ 65.6 ppm ([BP_3_^*i*Pr^]) and 78.8 ppm ([BP_2_^*t*Bu^Pz]) in a ca. 3:2 ratio, respectively. For **4** and **7**, two distinct 2:2 resonances are apparent in the high-field (“hydride”) regions of their ^1^H NMR spectra. For **3**, **4**, and **5**, H–Si and H–P coupling were not observable for the hydride resonances, presumably due to the presence of quadrupolar ^59^Co (*I* = 7/2) nuclei.^26^ The heterobimetallic silicide **7** is unique in this regard as fine structure of the Fe–*H* resonance is present (δ –14.49 ppm, ^2^*J*_HP_ = 16.9 Hz); notably, ^29^Si satellites for this resonance were not detectable in either the ^1^H or ^1^H{^31^P} NMR spectra of **7**, implying minimal coupling to the μ-silicon nucleus.

A notable feature of these silicides is the low-field ^29^Si resonances in their ^29^Si{^1^H} DEPT NMR spectra (δ 264–358 ppm; Table 1). These ^29^Si chemical shifts span a well-defined range that is diagnostic for this family of 3d-metal silicides and is consistent with structurally related, previously reported silicides.^17,19^ Notably, the 4d- and 5d-metal silicides in the Tp*(OC)_2_M≡Si–M(CO)_2_(L)Tp* class (M = Mo, W) generally possess lower-field ^29^Si resonances (δ 396–439).^16,18,27^

**Table 1:** Tabulated Experimental and Computed Metrical Parameters and Solution ^29^Si NMR Data for Silicide Complexes **3**, **4**, **5**, and **7**

species	δ_Si_ (ppm)^a^	*d*(Co^A^–Si)^b^ (Å)	*d*(Si–M′)^b^ (Å)	∠(Co–Si–M′) (degrees)	WBI (Co^A^–Si)^b^	WBI (Si–M′)	*q*Co^A^	*q*M′	*q*Si
**3**	305	2.086(2)	2.084(2)	169.00(5)					
3*		2.071	2.068	163.9	1.44	1.48	–0.30	0.00	+0.52
**4**	264								
**5**	333	2.0883(6)	2.0883(6)^c^	166.11(5)					
**5**^e^		2.071	2.070	163.0	1.44	1.44	–0.31	–0.31	+0.50
**7**	358, −5^d^	2.096(9)	2.096(9)^c^	169.0(4)					
7*		2.098	2.072	162.0	1.40	1.56	–0.34	–0.88	+0.53

a Recorded in benzene-*d*_6_ solution (119 MHz).

b Co^A^ is the [BP_3_^*i*Pr^]-ligated cobalt atom; M′ refers to the second metal atom in each complex (Co or Fe).

c Generated from symmetry equivalent fragments.

d Recorded in THF-*d*_8_ solution (119 MHz).

e Asterisks denote DFT-computed molecules.

Comparison of the solid-state molecular structures of **3**, **5**, and **7** reveal similar geometries for the [(H)_2_M=Si=M′(H)_2_] cores (M = Co, M′ = Co, Fe). The metric parameters for the solid-state structures are summarized in Table 1. In each case, the single-crystal X-ray diffraction data are of sufficient quality for location of the hydride ligands in the difference maps; accordingly, these hydrogen atoms were refined isotropically. For **3**, **5**, and **7**, the hydride ligands do not form a tetrahedral array about the μ-silicon atom, which would be expected for [M(μ-SiH_4_)M′] systems in which Si–H activation is minimal.^28,29^ Instead, the hydride ligands are oriented to accommodate a *pseudo*-octahedral coordination geometry for the associated metal centers (Figure 2b). Additionally, in **3**, **5**, and **7**, the hydride ligands of the neighboring [L(H)_2_M] units are canted toward one hemisphere of the molecule, leaving a face of the μ-silicon atom exposed. These features are consistent across **3**, **5**, and **7**, as shown in Figure 2b, which presents perspective views of the silicide solid-state structures along the Co···M′ axes. Coupled with the solution-state multinuclear NMR data for these species, the data strongly point toward assignment of these silicides as [(H)_2_M=Si=M′(H)_2_] rather than [M(μ-SiH_4_)M′] structures, in which significant residual Si···H interactions are present.^17,19,28,29^

The asymmetric unit in the crystal structure of **3** contains one nondisordered molecule of [BP_3_^*i*Pr^](H)_2_Co=Si=Co(H)_2_[BP_2_^*t*Bu^Pz], displaying close Co–Si contacts (*d*([BP_3_^*i*Pr^]Co–Si) = 2.086(2) Å, *d*([BP_2_^*t*Bu^Pz]Co–Si) = 2.084(2) Å), and a nearly linear Co–Si–Co linkage (∠= 169.00(5)°). For crystals of **5** and **7** grown from neat THF solutions at −35 °C, the asymmetric units of their crystal structures (in *C*2/*c*) contain half of a molecule; the full molecules are generated by a symmetry operation. For complex **7**, this feature of the solid-state molecular structure precludes the distinction of the M–Si bond lengths. The presence of a half-occupied silicon atom at the bridgehead site of the overlapping [BP_3_^*i*Pr^]/[SiP_3_^*i*Pr^] ligands is apparent from the electron density map, and the site is well-modeled as being occupied by 0.5 B and 0.5 Si atoms. Significant distortions of the atomic thermal parameters occurred when the site was modeled as either fully occupied boron or silicon.

Geometry optimizations (DFT) of **3**, **5**, and **7** were initiated from the crystallographic atomic coordinates at the ωB97X-D3/def2-TZVP(Co,Fe,Si,P),def2-SVP(C,H,B,N) level of theory. Across the series, the natural charge^30^ (*q*) distribution in the [CoSiM′] cores of (nontruncated) 3*, 5*, and 7* remains broadly consistent, with modest positive charge accumulation at the μ-silicon atom (Table 1). The small but generally slightly negative charges of the metal centers in 3*, 5*, and 7* indicate that the metal centers are not well-described as Co^V^ and Fe^IV^ in the oxidation state formalism, which is often ambiguous in cases of atoms engaged in highly covalent bonding.^31,32^

Analysis of the Wiberg bond indices^33^ (WBIs) of 3*, 5*, and 7* provided further insight into the bonding situation within these molecules (Table 1). The magnitudes of the metal–silicon WBIs are similar to those previously reported for [BP_2_^*t*Bu^Pz](H)_2_Co=Si=Co(H)_2_[BP_2_^*t*Bu^Pz] (1.47, 1.47)^17^ and [BP_3_^*i*Pr^](H)_2_Co=Si=Co(H)_2_(SiH_2_Ph)[(^*i*^Pr_2_PCH_2_)_2_BPh] (1.45, 1.44).^19^ In heterobimetallic silicide **7**, the differing Co···Si and Fe···Si WBIs potentially indicate a greater extent of Fe···Si multiple bonding. This effect may be related to a greater donation from iron, which can be rationalized by considering isolated [BP_3_^*i*Pr^]Co^I^ and [SiP_3_^*i*Pr^]Fe^0^ fragments. Also, the borate ligand of [BP_3_^*i*Pr^]Co^I^ introduces a formal positive charge at the metal center for the overall neutral fragment, perhaps contracting the valence metal-based orbitals. In contrast, the neutral [SiP_3_^*i*Pr^]Fe^0^ fragment formally possesses a zerovalent metal center, which is expected to more effectively mediate the cleavage of Si–H bonds.

Atoms in molecules (AIM)^34^ analyses of **3**, **5**, and **7** showed that the [H_2_Co=Si=MH_2_] (M = Fe, Co) cores in these silicides possess few or no bond paths/bond critical points connecting the μ-silicon centers and the flanking hydride ligands (Figure S21). One bond path connecting the hydride ligand oriented *cis* with respect to the N-donor atom of the [BP_2_^*t*Bu^Pz] ligand of **3** to the μ-silicon center is present, analogous to the situation found in [BP_2_^*t*Bu^Pz](H)_2_Co=Si=Co(H)_2_[BP_2_^*t*Bu^Pz].^17^ One such bond path is also present in **5**. These features may indicate a minor Si···H interaction;^19,35−38^ similarly, in the case of [BP_3_^*i*Pr^](H)_2_Co=Si=Co(H)_2_(SiH_2_Ph)[(^*i*^Pr_2_PCH_2_)_2_BPh],^19^ the value of ^1^*J*_Si–H_ (8 Hz) was found to be consistent with minimal Si–H bonding.

## 3 Conclusions

Base-stabilized silylene (SiH_2_) complex **1** has enabled facile, modular access to a series of symmetrical, unsymmetrical, and heterobimetallic silicides via Si···H bond activations. In certain cases, **1** allowed access to silicides that are not obtainable by direct L(H)_2_M=Si=M(H)_2_L formation from SiH_4_ and a (LM)_2_(μ-N_2_) precursor. For example, reaction of (Tp″Co)_2_(μ-N_2_) with SiH_4_ was found to generate mixtures containing (Tp″Co)_2_(μ-H)_1,2_ as opposed to a silicide product.^17^ This result implies that the presence of a bulky [BP_3_^*i*Pr^]Co unit stabilizes silicide structures that are inaccessible with other ligand combinations, as demonstrated by [BP_3_^*i*Pr^]Co/Tp″Co silicide **4**, which is isolable. Similarly, the symmetrical silicide **5** is inaccessible by direct treatment of Na(THF)_6_([BP_3_^*i*Pr^]CoI) or **6** with SiH_4_; in these cases, [BP_3_^*i*Pr^](H)_2_Co=Si=Co(H)_2_(SiH_3_)[(^*i*^Pr_2_PCH_2_)_2_BPh]^19^ or a mixture of **2** and **5** (both in ∼20% yield)^39^ is produced as the major silicon-containing product, respectively. Thus, **1** avoids degradation pathways involving the [BP_3_^*i*Pr^] ligand, such as –CH_2_P^*i*^Pr_2_ side-arm migration and coupling to generate [H_2_SiCH_2_P^*i*^Pr_2_] fragments, as seen in **2**. This feature is likely derived from the base stabilization in **1**, which tempers the high reactivity expected for a base-free terminal SiH_2_ complex.

The extension of silicide chemistry to heterobimetallic metal combinations, as in complex **7**, presents a new metal–silicon structural type and potentially new avenues for cooperative substrate activation and silicon atom functionalization that exploit diverse electronic properties of the metal centers. Significantly, precursor silylene complex **1** demonstrates a novel type of reactivity for transition-metal silylene complexes (i.e., metal-mediated Si–H bond activation of a coordinated silylene) and highlights the utility of [SiH_2_] synthons for accessing unusual metal–silicon bonding arrangements.

## Supporting Information Available

Experimental methods, detailed syntheses, details of VT-NMR spectroscopy, details of crystallography, details of calculations, NMR spectra, computed atomic coordinates (PDF)

## References

[ref1] Pachaly B.; Weis J. The Direct Process to Methylchlorosilanes: Reflections on Chemistry and Process Technology. In Organosilicon Chemistry III; John Wiley & Sons, Ltd, 1997; pp 478–483.

[ref2] Seyferth D. Dimethyldichlorosilane and the Direct Synthesis of Methylchlorosilanes. The Key to the Silicones Industry. Organometallics 2001, 20, 4978–4992. 10.1021/om0109051.

[ref3] Murarka S. P. Silicide Thin Films and Their Applications in Microelectronics. Intermetallics 1995, 3, 173–186. 10.1016/0966-9795(95)98929-3.

[ref4] Schmidt V.; Wittemann J. V.; Senz S.; Gösele U. Silicon Nanowires: A Review on Aspects of Their Growth and Their Electrical Properties. Adv. Mater. 2009, 21, 2681–2702. 10.1002/adma.200803754. 36751058

[ref5] Schmidt V.; Wittemann J. V.; Gösele U. Growth, Thermodynamics, and Electrical Properties of Silicon Nanowires. Chem. Rev. 2010, 110, 361–388. 10.1021/cr900141g. 20070117

[ref6] De Schutter B.; De Keyser K.; Lavoie C.; Detavernier C. Texture in Thin Film Silicides and Germanides: A Review. Appl. Phys. Rev. 2016, 3, 031302 10.1063/1.4960122.

[ref7] Ward W. Catalysis of the Rochow Direct Process. J. Catal. 1986, 100, 240–249. 10.1016/0021-9517(86)90089-8.

[ref8] Lorey L.; Roewer G. The Direct Synthesis of Methylchlorosilanes: New Aspects Concerning Its Mechanism. Silicon Chem. 2002, 1, 299–308. 10.1023/B:SILC.0000018400.24117.89.

[ref9] Xue G.; Green V. V. In Mechanistic Aspects of the Rochow Direct Process, Silicon for the Chemical and Solar Industry XIII; Kristiansand, Norway, 2016; pp 147–155.

[ref10] Sun D.-H.; Bent B. E.; Wright A. P.; Naasz B. M. Chemistry of the Direct Synthesis of Methylchlorosilanes from Methyl + Chlorine Monolayers on a Cu_3_Si Surface. Catal. Lett. 1997, 46, 127–132. 10.1023/A:1019025325414.

[ref11] Lewis K. M.; Rethwisch D. G. Catalyzed Direct Reactions of Silicon (Studies in Organic Chemistry); Elsevier: Amsterdam, 1993.

[ref12] Wanandi P. W.; Glaser P. B.; Tilley T. D. Reactivity of an Osmium Silylene Complex toward Chlorocarbons: Promotion of Metal Redox Chemistry by a Silylene Ligand and Relevance to the Mechanism of the Direct Process. J. Am. Chem. Soc. 2000, 122, 972–973. 10.1021/ja993089l.

[ref13] Waterman R.; Hayes P. G.; Tilley T. D. Synthetic Development and Chemical Reactivity of Transition-Metal Silylene Complexes. Acc. Chem. Res. 2007, 40, 712–719. 10.1021/ar700028b. 17477497

[ref14] Lipke M. C.; Liberman-Martin A. L.; Tilley T. D. Electrophilic Activation of Silicon-Hydrogen Bonds in Catalytic Hydrosilations. Angew. Chem., Int. Ed. 2017, 56, 2260–2294. 10.1002/anie.201605198. 27641142

[ref15] Hashimoto H.; Nagata K. Transition-Metal Complexes with Triple Bonds to Si, Ge, Sn, and Pb and Relevant Complexes. Chem. Lett. 2021, 50, 778–787. 10.1246/cl.200872.

[ref16] Ghana P.; Arz M. I.; Chakraborty U.; Schnakenburg G.; Filippou A. C. Linearly Two-Coordinated Silicon: Transition Metal Complexes with the Functional Groups M≡Si—M and M = Si = M. J. Am. Chem. Soc. 2018, 140, 7187–7198. 10.1021/jacs.8b02902. 29730935

[ref17] Handford R. C.; Nguyen T. T.; Teat S. J.; Britt R. D.; Tilley T. D. Direct Transformation of SiH_4_ to a Molecular L(H)_2_Co=Si=Co(H)_2_L Silicide Complex. J. Am. Chem. Soc. 2023, 145, 3031–3039. 10.1021/jacs.2c11569. 36696099

[ref18] Ghana P.; Rump J.; Schnakenburg G.; Arz M. I.; Filippou A. C. Planar Tetracoordinated Silicon (PtSi): Room-Temperature Stable Compounds Containing Anti-van’t Hoff/Le Bel Silicon. J. Am. Chem. Soc. 2021, 143, 420–432. 10.1021/jacs.0c11628. 33347313

[ref19] Handford R. C.; Smith P. W.; Tilley T. D. Activations of All Bonds to Silicon (Si–H, Si–C) in a Silane with Extrusion of [CoSiCo] Silicide Cores. J. Am. Chem. Soc. 2019, 141, 8769–8772. 10.1021/jacs.9b04265. 31117667

[ref20] Schneider H.; Schmidt D.; Eichhöfer A.; Radius M.; Weigend F.; Radius U. Synthesis and Reactivity of NHC-Stabilized Iron(II)–Mesityl Complexes. Eur. J. Inorg. Chem. 2017, 2017, 2600–2616. 10.1002/ejic.201700143.

[ref21] Straus D. A.; Zhang C.; Quimbita G. E.; Grumbine S. D.; Heyn R. H.; Tilley T. D.; Rheingold A. L.; Geib S. J. Silyl and Diphenylsilylene Derivatives of (η^5^-C_5_Me_5_)(PMe_3_)_2_Ru. Evidence for the Base-Free Silylene Complex [(η^5^-C_5_Me_5_)(PMe_3_)_2_Ru = SiPh_2_]^+^. J. Am. Chem. Soc. 1990, 112, 2673–2681. 10.1021/ja00163a030.

[ref22] Henkelman G.; Uberuaga B. P.; Jónsson H. A Climbing Image Nudged Elastic Band Method for Finding Saddle Points and Minimum Energy Paths. J. Chem. Phys. 2000, 113, 9901–9904. 10.1063/1.1329672.

[ref23] Neumeyer F.; Lipschutz M. I.; Tilley T. D. Group 8 Transition Metal Complexes of the Tripodal Triphosphino Ligands PhSi(CH_2_PR_2_)_3_ (R = Ph, *i*Pr): Group 8 Transition Metal Complexes of Triphosphino Ligands. Eur. J. Inorg. Chem. 2013, 2013, 6075–6078. 10.1002/ejic.201301074.

[ref24] Schild D. J.; Peters J. C. Light Enhanced Fe-Mediated Nitrogen Fixation: Mechanistic Insights Regarding H_2_ Elimination, HER, and NH_3_ Generation. ACS Catal. 2019, 9, 4286–4295. 10.1021/acscatal.9b00523. 31467770 PMC6715299

[ref25] Cavaillé A.; Joyeux B.; Saffon-Merceron N.; Nebra N.; Fustier-Boutignon M.; Mézailles N. Triphos–Fe Dinitrogen and Dinitrogen–Hydride Complexes: Relevance to Catalytic N _2_ Reductions. Chem. Commun. 2018, 54, 11953–11956. 10.1039/C8CC07466F. 30288508

[ref26] Stone N. J. Table of Nuclear Magnetic Dipole and Electric Quadrupole Moments. At. Data Nucl. Data Tables 2005, 90, 75–176. 10.1016/j.adt.2005.04.001.

[ref27] Ghana P. Synthesis, Characterization and Reactivity of Ylidyne and μ-Ylido Complexes Supported by Scorpionato Ligands. Springer Theses, Springer International Publishing: Cham, 2019.

[ref28] Atheaux I.; Donnadieu B.; Rodriguez V.; Sabo-Etienne S.; Chaudret B.; Hussein K.; Barthelat J.-C. A Unique Coordination of SiH_4_: Isolation, Characterization, and Theoretical Study of (PR_3_)_2_H_2_Ru(SiH_4_)RuH_2_(PR_3_)_2_. J. Am. Chem. Soc. 2000, 122, 5664–5665. 10.1021/ja000223p.

[ref29] Said R. B.; Hussein K.; Barthelat J.-C.; Atheaux I.; Sabo-Etienne S.; Grellier M.; Donnadieu B.; Chaudret B. Redistribution at Silicon by Ruthenium Complexes. Bonding Mode of the Bridging Silanes in Ru_2_H_4_(μ-η^2^:η^2^:η^2^:η^2^-SiH_4_)(PCy_3_)_4_ and Ru_2_H_2_(μ-η^2^:η^2^-H_2_Si(OMe)_2_)_3_(PCy_3_)_2_. Dalton Trans. 2003, 4139–4146. 10.1039/B304122K.

[ref30] Reed A. E.; Weinstock R. B.; Weinhold F. Natural Population Analysis. J. Chem. Phys. 1985, 83, 735–746. 10.1063/1.449486.

[ref31] Parkin G. Valence, Oxidation Number, and Formal Charge: Three Related but Fundamentally Different Concepts. J. Chem. Educ. 2006, 83, 791–799. 10.1021/ed083p791.

[ref32] Wolczanski P. T. Flipping the Oxidation State Formalism: Charge Distribution in Organometallic Complexes As Reported by Carbon Monoxide. Organometallics 2017, 36, 622–631. 10.1021/acs.organomet.6b00820.

[ref33] Wiberg K. B. Application of the Pople-Santry-Segal CNDO Method to the Cyclopropylcarbinyl and Cyclobutyl Cation and to Bicyclobutane. Tetrahedron 1968, 24, 1083–1096. 10.1016/0040-4020(68)88057-3.

[ref34] Bader R. F. W. Atoms in Molecules. Acc. Chem. Res. 1985, 18, 9–15. 10.1021/ar00109a003.

[ref35] Handford R. C.; Smith P. W.; Tilley T. D. Silylene Complexes of Late 3*d* Transition Metals Supported by *Tris*-Phosphinoborate Ligands. Organometallics 2018, 37, 4077–4085. 10.1021/acs.organomet.8b00635.

[ref36] Iluc V. M.; Hillhouse G. L. Arrested 1,2-Hydrogen Migration from Silicon to Nickel upon Oxidation of a Three-Coordinate Ni(I) Silyl Complex. J. Am. Chem. Soc. 2010, 132, 11890–11892. 10.1021/ja1052329. 20690602

[ref37] Price J. S.; Emslie D. J. H.; Britten J. F. Manganese Silylene Hydride Complexes: Synthesis and Reactivity with Ethylene to Afford Silene Hydride Complexes. Angew. Chem., Int. Ed. 2017, 56, 6223–6227. 10.1002/anie.201700863. 28295881

[ref38] Price J. S.; Emslie D. J. H. Interconversion and Reactivity of Manganese Silyl, Silylene, and Silene Complexes. Chem. Sci. 2019, 10, 10853–10869. 10.1039/C9SC04513A. 32206252 PMC7069235

[ref39] The reaction of **6** with SiH_4_ is described in the Supporting Information. See pages S6-S7 for additional information

