# Supplementary material for: A [CoSiH2] Silylene Synthon Provides Modular Access to Homo- and Heterobimetallic [Co=Si=M] (M = Co, Fe) Silicide Complexes
Source: J Am Chem Soc. 2023 Oct 31;145(45):24690–7. doi: 10.1021/jacs.3c07998 (PMC10655182; doi:10.1021/jacs.3c07998)
Supplement: Supplementary file 1 — ja3c07998_si_001.pdf [file ja3c07998_si_001.pdf]

SUPPORTING INFORMATION FOR

A [$CoSiH_2$] Silylene Synthon Provides Modular Access to Homo- and Hetero-Bimetallic [Co=Si=M] (M = Co, Fe) Silicide Complexes

Rex C. Handford,† T. Don Tilley†,*

†Department of Chemistry, University of California, Berkeley, Berkeley, California 94720, United States

*Email: tdtilley@berkeley.edu

TABLE OF CONTENTS:

Techniques and Methods	S1
Experimental Section	S2
Variable-Temperature NMR Spectroscopy	S9
X-Ray Crystallography	S11
Details of Computations	S19
UV-Visible Spectroscopy	S29
NMR Spectroscopy	S30
Appendix: Coordinates of Computed Structures	S53
References	S66

TECHNIQUES AND METHODS

General methods: All manipulations were carried out using standard Schlenk techniques or in inert atmosphere gloveboxes filled with dry dinitrogen. Solvents were stored over molecular sieves (3 Å) after collection from a JC Meyers Phoenix solvent purification system. Benzene-d_6 and THF-d_8 were degassed with three freeze-pump-thaw cycles and stored over activated molecular sieves (3 Å) for 48 h prior to use.

Reagents: Complexes- $[BP_3^{iPr}]CoI$,[1] $([BP_2^{tBu}Pz]Co)_2(\mu\text{-}N_2)$,[2] $(Tp''Co)_2(\mu\text{-}N_2)$,[2] $[SiP_3^{iPr}]FeCl$[3]- were prepared according to literature procedures. Triphenylborane (98%) was purchased from Strem Chemicals and used as received. 4-Dimethylaminopyridine (DMAP) was purchased from Aldrich and heated to 150 °C at 60 mTorr for 24 h prior to use. 1,2-Difluorobenzene was purchased from Oakwood Chemical and collected from a JC Meyers Phoenix solvent purification system. Silane (15% balance N_2) was purchased from Praxair Inc. and used as received.

NMR and IR spectroscopy: NMR spectra were recorded at the UC Berkeley College of Chemistry NMR facilities on Bruker Avance 400, 500, and 600 MHz spectrometers, and spectra were referenced to solvent residual signals (^1H, ^{13}C),[4] or to external references (^{31}P, ^{11}B, ^{29}Si). Unless otherwise indicated, all NMR spectra are reported at 292 K. Full-widths at half-maximum are denoted as $\omega_{1/2}$. Resonances obscured by the solvent residual signal(s) are not reported. Integrals and line widths at half maximum ($\omega_{1/2}$) for resonances in NMR spectra of paramagnetic compounds were calculated with the line fitting program in Mestrelab Mnova 14.[5] IR spectra were collected on a Thermo Scientific Nicolet iS10 FTIR spectrometer as KBr pellets, unless otherwise noted. Abbreviations for IR spectroscopy: br, broad; s, sharp; m, medium; w, weak.

UV-visible spectroscopy: UV-visible spectra were collected as toluene solutions in quartz cuvettes with a path length of 1 cm. Solutions were prepared and loaded into cuvettes sealed with Kontes PTFE stopcocks inside of a dinitrogen filled glovebox. Spectra were recorded on a Shimadzu UV-2600i spectrophotometer at 292 K.

Elemental analysis: Microanalyses were conducted by Dr. Elena Kreimer at the UC Berkeley College of Chemistry Microanalytical Facility.

Warning: Silane is a pyrophoric gas which reacts vigorously upon contact with air even at concentrations of 15%. The utmost care should be taken to ensure that gas regulators, tubing, and glassware are under an inert atmosphere prior to and after the use of silane. A flame-resistant lab coat, Neoprene gloves, and face shield are recommended.

A method for quenching SiH_4 contained in a Schlenk line is detailed as follows: prior to addition of SiH_4 to a reaction vessel, a pipet-tipped hose from the manifold is immersed in a 500 mL beaker filled with water, and the hose is then purged with N_2. The hose and pipet are then closed off from the manifold. Following all manipulations involving SiH_4, a positive pressure of N_2 is introduced to the Schlenk line, and the atmosphere within the manifold is flushed through the hose and into the water-filled beaker. The SiH_4 atmosphere will gently ignite once the bubbles emerging from the tip of the pipette reach the surface of the water, until all remaining SiH_4 has been removed from the manifold. However, this method is not effective at removing SiH_4 contained in the tubing connecting the manifold to the reaction vessel. Disconnecting the tubing from the vessel usually results in a brief flash and popping sound.

EXPERIMENTAL SECTION

Improved synthesis of [BP$_3^{iPr}$]Co(DMAP):

In a glovebox, [BP$_3^{iPr}$]CoI (0.5143 g; 0.7707 mmol) and a stirbar were loaded to a 20 mL vial and THF (5 mL) was added to the green solids. The mixture was stirred for 10 min until all solids were consumed, forming a clear dark green solution. A THF (2 mL) solution of DMAP (0.0942 g; 0.0771 mmol) was added to the reaction mixture; no color change was observed. A THF (2 mL) suspension of KC_8 was added dropwise over 2 min to the vigorously stirred reaction mixture, resulting in a rapid color change to dark brown. The reaction mixture was stirred for 2 h, and then filtered through a Celite plug (0.5 × 3 cm) supported by glass wool to a 20 mL vial. The volatile components were removed *in vacuo* from the clear, dark brown filtrate to produce dark brown-green solids. The solids were extracted in toluene (5 × 2 mL) and filtered through Celite (0.5 × 1 cm) supported by glass wool, removing a rust colored solid and producing a clear brown filtrate. The filtrate was concentrated to a final volume of 5 mL and maintained at –35 °C for 18 h to induce deposition of large green prisms of [BP$_3^{iPr}$]Co(DMAP). The analytically pure [BP$_3^{iPr}$]Co(DMAP) produced this way was isolated by decanting the supernatant, washing with room temperature (22 °C) pentane (3 × 2 mL), and exposing to vacuum. Yield: 0.4458 g (87%), over two crops. The purity of the starting material was checked by ^{1}H NMR spectroscopy and compared to an authentic sample.[1]

Preparation of [BP$_3^{iPr}$](H)$_2$CoSiH$_2$(DMAP) (1):

In a glovebox, [BP$_3^{iPr}$]Co(DMAP) (0.3604 g; 0.5440 mmol) and a stirbar were loaded to a 100 mL glass vessel (the volume of the vessel was determined prior to the reaction by measuring the volume of acetone required to fill it to the level of the PTFE tap). Toluene (13.7 mL) was added to the vessel, generating a dark brown colored solution (the volume of the headspace (86.3 mL) corresponds to the volume of approximately one equiv of SiH_4 (15% balance N_2) at 1 atm and 292 K). The vessel was sealed with a Kontes PTFE plug and connected to a Schlenk line. The reaction mixture was stirred vigorously until all solids were consumed. The reaction mixture was subjected to three freeze-pump-thaw cycles. Finally, the vessel was thawed in a water bath, and the solution was vigorously stirred. Upon introduction of SiH_4, a gradual color change to clear orange was observed over 30 min. After this time, the volatile components were removed *in vacuo* to produce a bright yellow-orange solid. The vessel was returned to the glovebox, and the solids were extracted in 1,2-difluorobenzene (5 × 2 mL). The clear dark orange extracts were filtered through a Celite plug (0.5 × 3 cm) supported by glass wool to a 20 mL vial. The filtrate was chilled to –35 °C. The cold solution was layered with pentane (10 mL) and maintained at –35 °C for 18 h to induce deposition of bright yellow masses of microcrystalline **1**. The analytically pure **1** produced this way was isolated by decanting the supernatant, washing with cold (–35 °C) pentane (3 × 2 mL), and exposing to vacuum. Yield: 0.3048 g (81%), over two crops. Crystals of **1** suitable for single-crystal X-ray diffraction analysis were grown by vapor diffusion of pentane into a dilute 1,2-difluorobenzene solution at –35 °C for 4 d.

Characterization data for 1: ^{1}H NMR (500 MHz, benzene-*d*$_6$): δ –15.44 (s, 2H, Co—*H*), 1.09 (br m, 6H, C*H*$_2$P^{i}Pr$_2$), 1.39 (m, 18H, ^{i}Pr C*H*$_3$), 1.50 (br m, 18H, ^{i}Pr C*H*$_3$), 1.76 (s, 6H, DMAP N(C*H*$_3$)$_2$), 1.97 (br m, 6H, ^{i}Pr C*H*), 5.48 (d, $^3J_{HH}$ = 7.5 Hz, 2H, DMAP 3-*H*), 6.36 (*pseudo*-q, J = 7.5 Hz, $^1J_{SiH}$ = 159 Hz, 2H, Si—*H*), 7.38 (tt, $^3J_{HH}$ = 7.2 Hz, $^4J_{HH}$ = 1.2 Hz, 1H, Ar *para-H*), 7.66 (t, $^3J_{HH}$ = 7.4 Hz, 2H, Ar *meta-H*), 8.21 (br m, 2H, Ar *ortho-H*), 8.37 (d, $^3J_{HH}$ = 7.5 Hz, 2H, DMAP 2-*H*). **^{31}P{^{1}H} NMR** (243 MHz, benzene-*d*$_6$): δ 62.6 (s). **^{11}B{^{1}H} NMR** (193 MHz, benzene-*d*$_6$): δ –13.3 (s). **^{13}C{^{1}H} NMR** (151 MHz, benzene-*d*$_6$): δ 17.12 (m, *C*H$_2$P^{i}Pr$_2$), 19.87 (s, ^{i}Pr *C*H$_3$), 21.25 (s, ^{i}Pr *C*H$_3$), 33.47 (s, ^{i}Pr *C*H), 38.19 (s, N(*C*H$_3$)$_2$), 105.94 (s, Ar-*C*), 123.28 (s, Ar-*C*), 127.41 (s, Ar-*C*), 132.54 (s, Ar-*C*), 145.71 (s, Ar-*C*), 155.21 (s, Ar-*C*). **^{29}Si—^{1}H HMBC NMR** (119 MHz, 600 MHz, benzene-*d*$_6$): δ 33. **IR** (cm^{-1}): 2027 (s, $\nu_{Si–H}$), 1885 (s, $\nu_{Co–H}$). **Anal. Calcd** for $C_{34}H_{67}BCoN_2P_3Si$: C, 58.79; H, 9.72; N, 4.03. Found: C, 58.91; H, 10.02; N, 3.68.

***In-situ* generation of Ph$_3$B-DMAP:**

In a glovebox, a benzene-*d*$_6$ (0.2 mL) solution of Ph$_3$B (0.0060 g; 0.025 mmol) was added to a benzene-*d*$_6$ (0.2 mL) solution of DMAP (0.0030 g; 0.025 mmol), generating a clear, colorless solution. The reaction mixture was transferred between the vials used in sample preparation to ensure complete mixing of the reagents. The reaction mixture was then transferred to a J. Young NMR tube. Benzene-*d*$_6$ (3 × 0.1 mL) was used to rinse the vials, and the washes were then combined with the reaction mixture. ^{1}H NMR spectroscopic analysis of the reaction mixture indicated complete conversion to the adduct, Ph$_3$B-DMAP.

Characterization data for Ph$_3$B-DMAP: ^{1}**H NMR** (500 MHz, benzene-d_6): δ 1.85 (s, 6H, DMAP N(CH_3)$_2$), 5.30 (d, $^3J_{HH}$ = 7.5 Hz, 2H, DMAP 3-*H*), 7.28 (t, $^3J_{HH}$ = 7.1 Hz, 3H, BPh$_3$ *para-H*), 7.41 (t, $^3J_{HH}$ = 7.5 Hz, 6H, BPh$_3$ *meta-H*), 7.74 (d, $^3J_{HH}$ = 7.4 Hz, 6H, BPh$_3$ *ortho-H*), 7.94 (d, $^3J_{HH}$ = 7.5 Hz, 2H, DMAP 2-*H*).

Preparation of [PhB(CH$_2$P^{i}Pr$_2$)$_2$](H)$_2$Co[κ^2-*Si,P*-SiH$_2$CH$_2$P^{i}Pr$_2$] (2):

In a glovebox, **1** (0.0432 g; 0.0622 mmol) was dissolved in toluene (8 mL), and the resulting bright orange suspension was stirred for 30 min to produce a clear orange solution. To this solution was added a toluene (1 mL) solution of Ph$_3$B (0.0152 g; 0.0628 mmol) dropwise; no color change was observed. After 30 min, the volatile components of the reaction mixture were removed *in vacuo*, producing orange solids. Pentane (1 mL) was incorporated into the solids, and the volatile components were again removed *in vacuo*. The solids were extracted in pentane (5 × 0.5 mL) and filtered through a glass wool plug to a 4 mL vial, trapping a flocculent colorless solid, which was identified as Ph$_3$B-DMAP by ^{1}H NMR spectroscopy. Yield: 0.0197 g; 89%. The clear orange extracts from this filtration were concentrated to 0.75 mL and maintained at –35 °C for 18 h to induce deposition of orange blocks of **2** suitable for single-crystal X-ray diffraction analysis. The analytically pure **2** produced this way was isolated by decanting the supernatant, washing with cold (–35 °C) pentane (3 × 0.5 mL), and exposing to vacuum. Yield: 0.0244 g; 67% (over two crops).

Characterization data for 2: ^{1}**H NMR** (500 MHz, benzene-d_6): δ –11.18 (br s, 2H, Co—*H*), 1.06 (dd, $^3J_{HP}$ = 14.1 Hz, $^3J_{HH}$ = 7.0 Hz, 6H, ^{i}Pr CH_3), 1.17-1.25 (overlapping m, 18H, ^{i}Pr CH_3), 1.29 (dd, $^3J_{HP}$ = 13.1 Hz, $^3J_{HH}$ = 6.9 Hz, 12H, ^{i}Pr CH_3), 1.48 (m, 2H, ^{i}Pr$_2$PCH_2SiH$_2$), 1.62 (pseudo-*d*, *J* = 10.0 Hz, 4H, PhB(CH_2P^{i}Pr$_2$)$_2$), 1.93 (pseudo-oct, *J* = 6.7 Hz, 2H, ^{i}Pr$_2$PCH$_2$SiH$_2$ ^{i}Pr C*H*), 2.25 (m, 4H, PhB(CH$_2$P^{i}Pr$_2$)$_2$ ^{i}Pr C*H*), 4.09 (br m, $^1J_{SiH}$ = 173.7 Hz, 2H, ^{i}Pr$_2$PCH$_2$SiH_2), 7.22 (t, $^3J_{HH}$ = 7.4 Hz, 1H, Ar *para-H*), 7.41 (t, $^3J_{HH}$ = 7.4 Hz, 2H, Ar *meta-H*), 7.66 (d, $^3J_{HH}$ = 7.4 Hz, 2H, Ar *ortho-H*). ^{31}**P{^{1}H} NMR** (203 MHz, benzene-d_6): δ 33.0 (br s, PhB(CH$_2$*P*^{i}Pr$_2$)$_2$, 35.7 (br s, ^{i}Pr$_2$*P*CH$_2$SiH$_2$). ^{13}**C{^{1}H} NMR** (151 MHz, benzene-d_6): δ 14.63 (m, PhB(*C*H$_2$P^{i}Pr$_2$)$_2$), 18.47 (s, ^{i}Pr *C*H$_3$), 18.87 (s, ^{i}Pr *C*H$_3$), 19.70 (s, ^{i}Pr *C*H$_3$), 19.80 (s, ^{i}Pr *C*H$_3$), 23.61 (br s, ^{i}Pr$_2$P*C*H$_2$SiH$_2$), 28.41 (s, ^{i}Pr C*H*), 31.17 (d, $^1J_{CP}$ = 9.5 Hz, ^{i}Pr C*H*), 125.57 (s, Ar-*C*), 132.25 (s, Ar-*C*), 135.58 (s, Ar-*C*), 156.08 (s, Ar-*C*). ^{29}**Si—^{1}H HMBC NMR** (119 MHz, 600 MHz, benzene-d_6): δ –46.8. **IR** (cm^{-1}): 2052 (br; s). **Anal. Calcd** for $C_{27}H_{57}BCoSiP_3$: C, 56.65; H, 10.04. Found: C, 57.12; H, 10.39.

Preparation of [BP$_3$^{iPr}](H)$_2$Co=Si=Co(H)$_2$[BP$_2$^{tBu}Pz] (3):

Method A: In a glovebox, **1** (0.0155 g; 0.0223 mmol) was dissolved in toluene (3 mL), and the resulting bright orange suspension was stirred for 30 min to produce a clear orange solution. To this solution was added a clear orange-brown toluene (1 mL) solution of ([BP$_2$^{tBu}Pz]Co)$_2$(μ-N$_2$) (0.0122 g; 0.0112 mmol), resulting in a color change to dark blue. The reaction mixture was stirred for 5 min, and a toluene (0.5 mL) solution of Ph$_3$B (0.0054 g; 0.0223 mmol) was added. No further color change was observed. After 30 min, the volatile components of the reaction mixture were removed *in vacuo*, producing colorless and dark blue solids. The solids were extracted in THF (3 × 0.25 mL) and filtered through a glass wool plug to a 4 mL vial. The clear dark blue extracts were chilled to –35 °C and layered with pentane (3 mL). Diffusion of pentane into the THF solution at –35 °C over 18 h induced deposition of large clear prisms of crystalline Ph$_3$B-DMAP. The dark blue supernatant was decanted and filtered through a glass wool plug. The colorless solids were washed with cold (–35 °C) ether (3 × 0.5 mL), and the pale blue washes were collected with the supernatant following filtration through a glass wool plug. The crystals of Ph$_3$B-DMAP were collected and their identity confirmed by ^{1}H NMR spectroscopy. Yield: 0.0077 g; 95%. The volatile components were removed from the filtered supernatant *in vacuo*. The dark blue solids were extracted in THF (3 × 0.5 mL) and filtered through a glass wool plug to a 4 mL vial. The filtrate was concentrated to a final volume of 0.5 mL and chilled to–35 °C. The cold solution was layered with (Me$_3$Si)$_2$O (3.5 mL), and the mixture was maintained at –35 °C for 48 h to induce deposition of dark blue crystalline prisms of **3** suitable for single-crystal X-ray diffraction analysis. The analytically pure **3** produced this way was isolated by decanting the supernatant, washing with cold (–35 °C) THF (3 × 0.5 mL), and exposing to vacuum. Yield: 0.0182 g; 74%.

Method B: In a glovebox, a vial was charged with a magnetic stirbar, **1** (0.0144 g; 0.0207 mmol), and [BP$_2$^{tBu}Pz]Co(DMAP) (0.0136 g; 0.0208 mmol). With gentle stirring of the solids, toluene (2 mL) was added, resulting in a immediate color change to dark brown. The reaction mixture was stirred for 15 min, resulting in a color change to dark blue. A toluene (2 mL) solution of Ph$_3$B (0.0101 g; 0.0417 mmol; two equiv) was added to the reaction mixture; no further color change was observed.

The volatile components of the reaction mixture were removed *in vacuo*, giving dark blue solids. The remainder of the workup follows that described in Method A. Yield of Ph_3B-DMAP: 0.0115 g; 76%. Yield of **3**: 0.0163 g; 71%.

Characterization data for 3: ^{1}**H NMR** (500 MHz, benzene-d_6): δ –24.58 (v br s, $\omega_{1/2}$ = 1253 Hz, 1H, [BP_2^{tBu}Pz](H)$_2$Co), –13.02 (s, $\omega_{1/2}$ = 9.9 Hz, [BP_3^{iPr}](H)$_2$Co), –9.91 (v br s, $\omega_{1/2}$ = 1281 Hz, 1H, [BP_2^{tBu}Pz](H)$_2$Co), 0.82 (br m, 6H, [BP_3^{iPr}] CH_2), 0.95 (d, $^{3}J_{HP}$ = 11.3 Hz. 18H, [BP_2^{tBu}Pz] ^{t}Bu CH_3), 1.03 (dd, $^{3}J_{HP}$ = 14.8 Hz, $^{3}J_{HH}$ = 10.7 Hz, 2H, [BP_2^{tBu}Pz] CH_2), 1.20 (dd, $^{3}J_{HP}$ = 11.1 Hz, $^{3}J_{HH}$ = 7.3 Hz, 18H, [BP_3^{iPr}] ^{i}Pr CH_3), 1.27 (m, 2H, [BP_2^{tBu}Pz] CH_2), 1.35 (d, $^{3}J_{HP}$ = 11.8 Hz, 18H, [BP_2^{tBu}Pz] ^{t}Bu CH_3), 1.36 (m, 18H, [BP_3^{iPr}] ^{i}Pr CH_3), 1.70 (m, 6H, [BP_3^{iPr}] ^{i}Pr CH), 5.81 (t, $^{3}J_{HH}$ = 2.1 Hz, 1H, Pz 4-H), 7.21 (d, $^{3}J_{HH}$ = 2.4 Hz, 1H, Pz 3-H), 7.36 (t, $^{3}J_{HH}$ = 7.3 Hz, 1H, Ar *para-H*), 7.38 (t, $^{3}J_{HH}$ = 7.5 Hz, 1H, Ar *para-H*), 7.54 (t, $^{3}J_{HH}$ = 7.4 Hz, 2H, Ar *meta-H*), 7.61 (t, $^{3}J_{HH}$ = 7.4 Hz, 2H, Ar *meta-H*), 7.97 (d, $^{3}J_{HH}$ = 7.3 Hz, 2H, Ar *ortho-H*), 7.97 (d, $^{3}J_{HH}$ = 1.9 Hz, Pz 5-H), 8.10 (d, $^{3}J_{HH}$ = 7.2 Hz, 2H, Ar *ortho-H*). ^{31}**P{^{1}H} NMR** (203 MHz, benzene-d_6): δ 65.6 (br s, [BP_3^{iPr}]), 78.8 (s, [BP_2^{tBu}Pz]). ^{11}**B{^{1}H} NMR** (193 MHz, benzene-d_6): δ –13.3 (s, [BP_3^{iPr}]), –12.6 (s, [BP_2^{tBu}Pz]). ^{13}**C{^{1}H} NMR** (151 MHz, benzene-d_6): δ 14.33 (br m, BCH_2), 15.88 (br m, BCH_2), 19.28 (s, CH$_3$), 21.43 (s, CH$_3$), 29.86 (s, CH$_3$), 31.86 (m, [BP_3^{iPr}] ^{i}Pr CH), 32.71 (s, CH$_3$), 32.87 (m, [BP_2^{tBu}Pz] C(CH$_3$)$_3$), 36.27 (m, [BP_2^{tBu}Pz] C(CH$_3$)$_3$), 107.06 (s, Ar-C), 124.02 (s, Ar-C), 126.09 (s, Ar-C), 127.66 (s, Ar-C), 128.47 (s, Ar-C), 132.01 (s, Ar-C), 133.76 (s, Ar-C), 136.86 (s, Ar-C), 148.23 (s, Ar-C). ^{29}**Si DEPT NMR** (119 MHz, benzene-d_6): δ 305. **IR** (cm^{-1}): 1870 (w), 1774 (w). **Anal. Calcd** for $C_{54}H_{105}B_2Co_2N_2P_5Si$: C, 58.70; H, 9.58; N, 2.54. Found: C, 59.06; H, 9.52; N, 2.58.

NMR spectroscopic analysis of the reaction between 1 and 0.5 equiv of ([BP_2^{tBu}Pz]Co)$_2$(μ-N_2):

In a glovebox, finely crushed **1** (0.0060 g; 0.0086 mmol) was dissolved in benzene-d_6 (0.2 mL) to form a bright orange solution. To this solution was added a benzene-d_6 (0.2 mL) solution of ([BP_2^{tBu}Pz]Co)$_2$(μ-N_2) (0.0047 g; 0.0043 mmol), resulting in an immediate color change to dark blue. The reaction mixture was transferred between the vials used in sample preparation to ensure complete mixing of the reagents. The reaction mixture was then filtered through a glass wool plug to a J. Young NMR tube. Benzene-d_6 (3 × 0.1 mL) was used to rinse the vials and glass wool plug, and the washes were then combined with the reaction mixture. An internal standard of $(Me_3Si)_2O$ (1.0 μL) was added to the J. Young NMR tube. Multinuclear (^{1}H, ^{31}P{^{1}H}) NMR spectroscopy indicated the full consumption of the starting materials, with **3** (93%) and DMAP (88%) being generated, in addition to small quantities of [BP_2^{tBu}Pz]Co(DMAP) (3%). The J. Young NMR tube was returned to the glovebox, and a benzene-d_6 (0.2 mL) solution of Ph_3B (0.0021 g; 0.0087 mmol) was added to the reaction mixture. No color change was observed. Residual Ph_3B was transferred to the J. Young NMR tube with benzene-d_6 (3 × 0.05 mL). ^{1}H NMR spectroscopic analysis of the reaction mixture showed that the quantity of **3** was unchanged. However, [BP_2^{tBu}Pz]Co(DMAP)[2] was no longer detectable, and Ph_3B-DMAP (97%) was present in near quantitative yield. This result suggests that prior to the addition of Ph_3B, a small fraction of DMAP present in solution is sequestered by other species (e.g. organometallic byproducts). When Ph_3B is introduced, DMAP is quantitatively captured to generate the Ph_3B-DMAP adduct.

Preparation of [BP_3^{iPr}](H)$_2$Co=Si=Co(H)$_2$Tp" (4):

In a glovebox, **1** (0.0260 g; 0.0374 mmol) was dissolved in toluene (3 mL), and the resulting bright orange suspension was stirred for 30 min to produce a clear orange solution. To this solution was added a dark brown ether/toluene (1 mL / 2 mL) solution of (Tp"Co)$_2$(μ-N_2) (0.0202 g; 0.0188 mmol), resulting in a clear dark green-brown solution. The reaction mixture was stirred for 1 h, resulting in a color change to deep blue. A toluene (0.5 mL) solution of Ph_3B (0.0091 g; 0.038 mmol) was added. No further color change was observed. After 30 min, the volatile components of the reaction mixture were removed *in vacuo*, producing colorless and dark blue solids. The dark blue solids were extracted from the mixture with ether (5 × 2 mL), and the clear, dark blue extracts were filtered through a glass wool plug to a 20 mL vial and concentrated to a dark blue solid. The remaining colorless solids were identified by ^{1}H NMR spectroscopy as spectroscopically pure Ph_3B-DMAP. Yield: 0.0121 g; 89%. The blue solids were extracted in ether (3 × 0.5 mL) and filtered through a glass wool plug to a 4 mL vial. The filtrate was concentrated to a final volume of 1 mL and maintained at –35 °C to induce deposition of dark blue aggregates of **4**. The analytically pure **4** produced this way was isolated by decanting the supernatant, washing with cold (–35 °C) pentane (3 × 0.5 mL), and exposing to vacuum. Yield: 0.0321 g; 78%, over two crops.

Characterization data for 4: **^{1}H NMR** (500 MHz, benzene-d_6): δ –24.25 (s, 2H, Tp"(H)$_2$Co), –12.60 (s, 2H, [BP$_3^{iPr}$](H)$_2$Co), 0.85 (m, 6H, [BP$_3^{iPr}$] CH_2), 1.23 (overlapping m, 54H, Tp" ^{i}Pr CH_3 and [BP$_3^{iPr}$] ^{i}Pr CH_3), 1.43 (dd, $^3J_{HP}$ = 14.0 Hz, $^3J_{HH}$ = 7.4 Hz, 18H, [BP$_3^{iPr}$] ^{i}Pr CH_3), 1.76 (pseudo-oct, J = 7.1 Hz, 6H, [BP$_3^{iPr}$] ^{i}Pr CH), 3.57 (sept, $^3J_{HH}$ = 6.8 Hz, 3H, Tp" ^{i}Pr CH), 3.65 (sept, $^3J_{HH}$ = 7.0 Hz, 3H, Tp" ^{i}Pr CH), 5.01 (br s, 1H, Tp" B—H), 5.98 (s, 3H, Tp" Pz 4-H), 7.36 (t, $^3J_{HH}$ = 7.2 Hz, 1H, [BP$_3^{iPr}$] *para-H*), 7.62 (t, $^3J_{HH}$ = 7.5 Hz, 2H, [BP$_3^{iPr}$] *meta-H*), 7.98 (d, $^3J_{HH}$ = 7.4 Hz, 2H, [BP$_3^{iPr}$] *ortho-H*). **^{31}P{^{1}H} NMR** (203 MHz, benzene-d_6): δ 62.7 (br s, [BP$_3^{iPr}$]). **^{11}B{^{1}H} NMR** (193 MHz, benzene-d_6): δ –13.2 (s, [BP$_3^{iPr}$]), –9.6 (s, Tp"). **^{13}C{^{1}H} NMR** (151 MHz, benzene-d_6): δ 15.79 (br m, [BP$_3^{iPr}$] CH$_2$), 19.11 (d, $^2J_{CP}$ = 4.6 Hz, [BP$_3^{iPr}$] ^{i}Pr CH$_3$), 21.00 (s, ^{i}Pr CH$_3$), 23.74 (s, ^{i}Pr CH$_3$), 23.89 (s, ^{i}Pr CH$_3$), 26.80 (s, Tp" ^{i}Pr CH), 30.35 (s, Tp" ^{i}Pr CH), 31.96 (br d, $^1J_{CP}$ = 16.9 Hz, [BP$_3^{iPr}$] ^{i}Pr CH), 98.74 (s, Ar-C), 124.04 (s, Ar-C), 132.00 (s, Ar-C), 156.69 (s, Ar-C), 163.24 (s, Ar-C). **^{29}Si DEPT NMR** (119 MHz, benzene-d_6): δ 264. **IR** (cm^{-1}): 2540 (m, $\nu_{B\text{–}H}$), 2078 ($\nu_{Co\text{–}H}$), 1944 ($\nu_{Co\text{–}H}$). **Anal. Calcd** for $C_{54}H_{103}B_2Co_2N_6P_3Si$: C, 59.13; H, 9.46; N, 7.66. Found: C, 59.50; H, 9.55; N, 7.33.

NMR spectroscopic analysis of the reaction between 1 and 0.5 equiv of (Tp"Co)$_2$(μ-N$_2$):

In a glovebox, finely crushed **1** (0.0052 g; 0.0075 mmol) was dissolved in benzene-d_6 (0.2 mL) to form a bright orange solution. To this solution was added a benzene-d_6 (0.2 mL) solution of (Tp"Co)$_2$(μ-N$_2$) (0.0040 g; 0.0037 mmol), resulting in an immediate color change to dark greenish blue. The reaction mixture was transferred between the vials used in sample preparation to ensure complete mixing of the reagents. The reaction mixture was then filtered through a glass wool plug to a J. Young NMR tube. Benzene-d_6 (3 × 0.1 mL) was used to rinse the vials and glass wool plug, and then combined with the reaction mixture. An internal standard of (Me$_3$Si)$_2$O (1.0 μL) was added to the J. Young NMR tube. Over 15 min, the color of the reaction mixture changed to dark blue. Multinuclear (^{1}H, ^{31}P{^{1}H}) NMR spectroscopy indicated the full consumption of the starting materials, with **4** (85%) and DMAP (91%) being generated. The J. Young NMR tube was returned to the glovebox, and a benzene-d_6 (0.2 mL) solution of Ph$_3$B (0.0018 g; 0.0074 mmol) was added to the reaction mixture. No color change was observed. Residual Ph$_3$B was transferred to the J. Young NMR tube with benzene-d_6 (3 × 0.05 mL). ^{1}H NMR spectroscopic analysis of the reaction mixture showed that the quantity of **4** was unchanged, and Ph$_3$B-DMAP (82%) was present in high yield. This result suggests that prior to the addition of Ph$_3$B, a small fraction of DMAP present in solution is sequestered by other species (e.g. organometallic byproducts); when Ph$_3$B is introduced, DMAP is quantitatively captured to generate the Ph$_3$B-DMAP adduct.

Preparation of [BP$_3^{iPr}$](H)$_2$Co=Si=Co(H)$_2$[BP$_3^{iPr}$] (5):

In a glovebox, a dark brown THF (0.5 mL) solution of [BP$_3^{iPr}$]Co(DMAP) (0.0305 g; 0.0460 mmol) was added to a gently stirring clear orange THF (0.5 mL) solution of **1** (0.0320 g; 0.0461 mmol), producing a clear, dark brown reaction mixture. Multinuclear (^{1}H, ^{31}P{^{1}H}) NMR spectroscopic analysis of the mixture in the presence of an internal standard ((Me$_3$Si)$_2$O) indicated that the starting materials remained in solution with no conversion to new products. A THF (0.5 mL) solution of Ph$_3$B (0.0223 g; 0.0921 mmol; two equiv) was added to the stirred reaction mixture, resulting in an immediate color change to dark magenta-red. Multinuclear (^{1}H, ^{31}P{^{1}H}) NMR spectroscopic analysis of the mixture in the presence of an internal standard ((Me$_3$Si)$_2$O) indicated that **5** and Ph$_3$B-DMAP were formed as the exclusive products of the reaction in quantitative yield. The dark red reaction mixture was filtered through a glass wool plug to a 4 mL vial and concentrated to a final volume of 1.0 mL. Maintenance of the filtrate at –35 °C for 48 h induced deposition of dark red crystalline prisms of **5** suitable for single-crystal X-ray diffraction analysis. The analytically pure **5** produced this way was isolated by decanting the supernatant, washing with cold (–35 °C) THF (3 × 0.5 mL), and exposing to vacuum. Yield: 0.0384 g; 75%, over two crops.

Following isolation of **5**, the supernatant was concentrated to a volume of 1.0 mL and layered with pentane (3.0 mL). Diffusion of pentane into the THF solution at –35 °C over 18 h to induced deposition of large clear prisms of crystalline Ph$_3$B-DMAP. The dark orange supernatant was decanted and filtered through a glass wool plug. The colorless solids were washed with cold (–35 °C) ether (3 × 0.5 mL), and the washes were discarded. The crystals of Ph$_3$B-DMAP were collected and their identity confirmed by ^{1}H NMR spectroscopy. Yield: 0.0125 g; 74%.

Characterization data for 5: **^{1}H NMR** (600 MHz, benzene-d_6): δ –13.16 (s, 4H, Co—H), 0.81 (br s, 12H, CH_2P^{i}Pr$_2$), 1.20 (br dd, $^3J_{HP}$ = 10.5 Hz, $^3J_{HH}$ = 6.0 Hz, 36H, ^{i}Pr CH_3), 1.35 (br dd, $^3J_{HP}$ = 14.6 Hz, $^3J_{HH}$ = 7.2 Hz, 36H, ^{i}Pr CH_3), 1.71 (m, 12H, ^{i}Pr CH), 7.35 (t, $^3J_{HH}$ = 7.4 Hz, 2H, Ar *para-H*), 7.60 (t, $^3J_{HH}$ = 7.5 Hz, 4H, Ar *meta-H*), 7.95 (d, $^3J_{HH}$ = 7.3 Hz, 4H, Ar *ortho-H*). **^{31}P{^{1}H} NMR** (243 MHz,

benzene-d_6): δ 65.5 (s). **^{11}B{^{1}H} NMR** (193 MHz, benzene-d_6): δ –13.5 (s). **^{13}C{^{1}H} NMR** (151 MHz, benzene-d_6): δ 16.03 (m, $CH_2P^iPr_2$), 19.25 (s, ^{i}Pr CH_3), 21.31 (s, ^{i}Pr CH_3), 32.46 (m, ^{i}Pr C*H*), 124.04 (s, Ar-*C*), 127.65 (s, Ar-*C*), 132.00 (s, Ar-*C*). **^{29}Si DEPT NMR** (119 MHz, benzene-d_6): δ 333. **IR** (cm^{-1}): 1786 (s, $\nu_{Co–H}$). **Anal. Calcd** for $C_{54}H_{110}B_2Co_2P_6Si$: C, 58.28; H, 9.96. Found: C, 58.33; H, 10.04.

In-situ **preparation of ([BP$_3^{iPr}$]Co)$_2$(μ-N$_2$) (6):**

Method A. In a glovebox, blue-purple crystals Na(THF)$_6$([BP$_3^{iPr}$]CoI) (0.0109 g; 0.00975 mmol) were dissolved in benzene-d_6 (0.6 mL), resulting in an immediate color change to dark green. The reaction mixture was filtered through a glass wool plug to a J. Young NMR tube, removing a colorless solid (presumably NaI). The vial used in sample preparation and glass wool plug were rinsed with benzene-d_6 (3 × 0.05 mL); the washes were then filtered to the J. Young NMR tube. An internal standard of $(Me_3Si)_2O$ (1.0 µL) was added to the J. Young NMR tube. ^{1}H NMR spectroscopic analysis of the reaction mixture indicated clean formation of paramagnetic **6** (86% yield), determined *via* integration against the internal standard. Solutions of **6** decompose upon exposure to vacuum, as evidenced by an immediate color change to dark brown and the appearance of multiple products in the ^{1}H NMR spectrum. Additionally, **6** is thermally unstable in solution, discoloring to brown after 72 h in benzene-d_6 solution.

Crystals of **6** were grown by addition of $(Me_3Si)_2O$ (2.5 mL) to the reaction mixture, forming a homogeneous dark green solution. Maintenance of the solution at –35 °C over 18 h to induced deposition of dark green blocks of **6** suitable for single-crystal X-ray diffraction analysis. Crystalline **6** was isolated following removal of the supernatant and washed with cold (–35 °C) pentane (3 × 0.5 mL). In general, the yield of **6** following crystallization is low (19%), and crystals of **6** are sensitive to vacuum, as evidenced by the results of microanalysis.

Method B. In a glovebox, a benzene-d_6 (0.1 mL) solution of Ph_3B (0.0031 g; 0.013 mmol) was added to a clear, dark brown benzene-d_6 (0.2 mL) suspension of [BP$_3^{iPr}$]Co(DMAP) (0.0083 g; 0.013 mmol), resulting in an immediate color change to dark green. The reaction mixture was transferred between the vials used in sample preparation to ensure complete mixing of the reagents. The reaction mixture was then filtered through a glass wool plug to a J. Young NMR tube. Benzene-d_6 (3 × 0.1 mL) was used to rinse the vials and glass wool plug, and the washes were then combined with the reaction mixture. An internal standard of $(Me_3Si)_2O$ (1.0 µL) was added to the J. Young NMR tube. ^{1}H NMR spectroscopic analysis of the reaction mixture indicated clean formation of paramagnetic **6** (87% yield), and Ph_3B-DMAP (97% yield) determined *via* integration against the internal standard.

Characterization data for 6: ^{1}H NMR (400 MHz, benzene-d_6): δ –0.70 (br s, $\omega_{1/2}$ = 49 Hz, 18H, [BP$_3^{iPr}$] C*H*$_3$), 12.64 (br s, $\omega_{1/2}$ = 193 Hz, 6H), 13.82 (br s, $\omega_{1/2}$ = 28 Hz, 18H, BP$_3^{iPr}$ C*H*$_3$), 47.12 (6H). Magnetic moment (Evans' method; benzene-d_6, 400 MHz): 2.5 μ_B. Resonances corresponding to the phenyl group proton nuclei were not found. **Anal. Calcd** for $C_{54}H_{106}B_2Co_2N_2P_6$: C, 58.50; H, 9.64; N, 2.52. Found: C, 57.38; H, 8.90; N, 1.60.

Reaction of 6 with one equiv of SiH$_4$:

In a glovebox, **6** was prepared by abstraction of DMAP from [BP$_3^{iPr}$]Co(DMAP) (0.0083 g; 0.013 mmol) by Ph_3B (0.0031 g; 0.013 mmol) in the manner described above, in benzene-d_6 solution. The dark green reaction mixture was filtered through a glass wool plug to a J. Young NMR tube with an internal volume of 2.6 mL (the volume of the vessel was determined prior to the reaction by measuring the volume of acetone required to fill it to the level of the PTFE tap). The vials used sample preparation were rinsed with aliquots benzene-d_6 (3 × 0.01 mL) that were then added to the J. Young NMR tube *via* the glass wool plug. An internal standard of $(Me_3Si)_2O$ (1.0 µL) was added. Additional benzene-d_6 was added for a total liquid volume of 1.95 mL. A ^{1}H NMR spectrum of the reaction mixture was recorded, which showed clean formation of **6** and Ph_3B-DMAP. The J. Young NMR tube was connected to a Schlenk line, and its contents frozen at –78 °C. The tube was evacuated and backfilled with SiH_4 (15% balance N_2, 1 atm), corresponding to one equiv SiH_4 (based on **6**). The tube was sealed, and its contents were then thawed in a water bath. Upon shaking, a rapid color change to clear orange was observed. Over 3 min, the color of the reaction mixture changed to dark red, and finally to dark magenta. The ^{1}H and ^{31}P{^{1}H} NMR spectra of the reaction mixture indicated the full consumption of **6**, and formation of **5** (20% yield based on Co) and **2** (23% yield based on Co). The ^{1}H and ^{31}P{^{1}H} NMR spectra indicated the formation of several other unidentified diamagnetic products.

Reaction of 6 with two equiv of SiH_4:

In a glovebox, **6** was prepared by abstraction of DMAP from $[BP_3^{iPr}]$Co(DMAP) (0.0083 g; 0.013 mmol) by Ph_3B (0.0031 g; 0.013 mmol) in the manner described above, in benzene-*d*$_6$ solution. The dark green reaction mixture was filtered through a glass wool plug to a J. Young NMR tube with an internal volume of 2.6 mL (the volume of the vessel was determined prior to the reaction by measuring the volume of acetone required to fill it to the level of the PTFE tap). The vials used sample preparation were rinsed with aliquots benzene-*d*$_6$ (3 × 0.01 mL) that were then added to the J. Young NMR tube *via* the glass wool plug. An internal standard of $(Me_3Si)_2O$ (1.0 μL) was added. Additional benzene-*d*$_6$ was added for a total liquid volume of 1.2 mL. A ^1H NMR spectrum of the reaction mixture was recorded, which showed clean formation of **6** and Ph_3B-DMAP. The J. Young NMR tube was connected to a Schlenk line, and its contents frozen at –78 °C. The tube was evacuated and backfilled with SiH_4 (15% balance N_2, 1 atm), corresponding to two equiv SiH_4 (based on **6**). The tube was sealed, and its contents were then thawed in a water bath. Upon shaking, a rapid color change to dark reddish-brown was observed. The ^1H and $^{31}P\{^1H\}$ NMR spectra of the reaction mixture indicated formation of a mixture of intractable products. No detectable quantities of **5** or **2** were present in the reaction mixture.

Reaction of 6 with 1 and Ph_3B:

In a glovebox, **6** was prepared by abstraction of DMAP from $[BP_3^{iPr}]$Co(DMAP) (0.0054 g; 0.0082 mmol) by Ph_3B (0.0020 g; 0.0083 mmol) in the manner described above, in benzene-*d*$_6$ solution. The dark green reaction mixture was filtered through a glass wool plug to a J. Young NMR tube. The vials used sample preparation were rinsed with aliquots benzene-*d*$_6$ (3 × 0.01 mL) that were then added to the J. Young NMR tube *via* the glass wool plug. An internal standard of $(Me_3Si)_2O$ (1.0 μL) was added. A ^1H NMR spectrum of the reaction mixture was recorded, which showed clean formation of **6** and Ph_3B-DMAP. The tube was returned to a glovebox, and a benzene-*d*$_6$ (0.1 mL) solution of **1** (0.0056 g; 0.0081 mmol) was added to the reaction mixture, resulting in an instantaneous color change to dark red. The vial containing **1** was rinsed with aliquots benzene-*d*$_6$ (3 × 0.01 mL) that were then added to the J. Young NMR tube. Multinuclear NMR spectroscopic analysis (^1H and $^{31}P\{^1H\}$) NMR indicated full consumption of **6**, formation of $[BP_3^{iPr}]$Co(DMAP) (44%, based on **1**) and **5** (56%, based on **1**), and the presence of unconverted **1** (46% remaining). The tube was returned to a glovebox, and a benzene-*d*$_6$ (0.1 mL) solution of Ph_3B (0.0020 g; 0.0083 mmol) was added to the reaction mixture, resulting in a further color change to dark magenta-red. The vial containing Ph_3B was rinsed with aliquots benzene-*d*$_6$ (3 × 0.01 mL) that were then added to the J. Young NMR tube. Multinuclear NMR spectroscopic analysis (^1H and $^{31}P\{^1H\}$) NMR indicated that **5** (93%) and Ph_3B-DMAP (>99%, based on 0.0017 mmol of total Ph_3B added) were the exclusive products in the NMR spectra.

Preparation of $[BP_3^{iPr}](H)_2Co{=}Si{=}Fe(H)_2[SiP_3^{iPr}]$ (7):

In a glovebox, a THF (1 mL) suspension of KC_8 (0.0058 g; 0.043 mmol) was added dropwise to a stirred THF (3 mL) solution of $[SiP_3^{iPr}]$FeCl (0.0254 g; 0.0430 mmol) at –35 °C, resulting in a rapid color change from clear forest green to dark red. After 20 min, the reaction mixture was filtered through a tightly packed Celite plug (0.5 × 3 cm) supported by glass wool to a 20 mL vial containing a stirbar. A clear orange THF (3 mL) solution of **1** (0.0299 g; 0.0430 mmol) was added to the gently stirring reaction mixture; no color change was observed. The ^1H and $^{31}P\{^1H\}$ NMR spectra of the reaction mixture at this point indicated that there was no reaction of the starting materials. A THF (1 mL) solution of Ph_3B (0.0104 g; 0.0430 mmol) was added to the reaction mixture dropwise, resulting in a color change to dark reddish-purple. The reaction mixture was filtered through a glass wool plug to a 4 mL vial and concentrated to a final volume of 1.0 mL. Maintenance of the filtrate at –35 °C for 48 h induced deposition of dark red crystalline prisms of **7** suitable for single-crystal X-ray diffraction analysis. The analytically pure **7** produced this way was isolated by decanting the supernatant, washing with cold (–35 °C) THF (3 × 0.5 mL), and exposing to vacuum. Yield: 0.0215 g; 44%, over two crops.

Following isolation of **7**, the supernatant was concentrated to a volume of 1.0 mL and layered with pentane (3.0 mL). Diffusion of pentane into the THF solution at –35 °C over 18 h to induced deposition of large clear prisms of crystalline Ph_3B-DMAP. The dark orange supernatant was decanted and filtered through a glass wool plug. The colorless solids were washed with cold (–35 °C) ether (3 × 0.5 mL), and the washes were discarded. The crystals of Ph_3B-DMAP were collected and their identity confirmed by ^1H NMR spectroscopy. Yield: 0.0120 g; 77%.

Characterization data for 7: **^{1}H NMR** (600 MHz, benzene-d_6): –14.49 (q, $^2J_{HP}$ = 16.9 Hz, 2H, [SiP$_3^{iPr}$](H)$_2$Fe), –13.25 (s, 2H, [BP$_3^{iPr}$](H)$_2$Co), 0.90 (br s, 6H, [BP$_3^{iPr}$] CH_2), 1.02 (d, $^2J_{HP}$ = 8.3 Hz, 6H, [SiP$_3^{iPr}$] CH_2), 1.19 (dd, $^3J_{HP}$ = 10.5 Hz, $^3J_{HH}$ = 7.2 Hz, 18H, [SiP$_3^{iPr}$] ^{i}Pr CH_3), 1.33 (dd, $^3J_{HP}$ = 9.3 Hz, $^3J_{HH}$ = 6.8 Hz, 18H, [BP$_3^{iPr}$] ^{i}Pr CH_3), 1.46 (dd, $^3J_{HP}$ = 14.0 Hz, $^3J_{HH}$ = 6.7 Hz, 18H, [SiP$_3^{iPr}$] ^{i}Pr CH_3), 1.54 (dd, $^3J_{HP}$ = 12.9 Hz, $^3J_{HH}$ = 7.4 Hz, 18H, [BP$_3^{iPr}$] ^{i}Pr CH_3), 1.65 (br m, 6H, [SiP$_3^{iPr}$] ^{i}Pr CH), 1.85 (m, 6H, [BP$_3^{iPr}$] ^{i}Pr CH), 7.28 (overlapping m, 3H, Ar-H), 7.35 (t, $^3J_{HH}$ = 7.3 Hz, 1H, Ar *para-H*), 7.52 (dd, $^3J_{HH}$ = 7.8 Hz, $^4J_{HH}$ = 1.6 Hz, 2H, [SiP$_3^{iPr}$] *ortho-H*), 7.61 (t, $^3J_{HH}$ = 7.4 Hz, 2H, Ar *meta-H*), 8.06 (d, $^3J_{HH}$ = 7.0 Hz, 2H, [BP$_3^{iPr}$] *para-H*). **^{31}P{^{1}H} NMR** (203 MHz, benzene-d_6): δ 62.6 (s, [BP$_3^{iPr}$]), 71.4 (s, [SiP$_3^{iPr}$]). **^{11}B{^{1}H} NMR** (193 MHz, benzene-d_6): δ –13.6. **^{13}C{^{1}H} NMR** (151 MHz, benzene-d_6): δ 8.88 (d, $^1J_{CP}$ = 5.3 Hz, [SiP$_3^{iPr}$] CH$_2$), 16.81 (m, [BP$_3^{iPr}$] CH$_2$), 19.45 (d, $^2J_{CP}$ = 3.4 Hz, ^{i}Pr CH$_3$), 19.5 (s, ^{i}Pr CH$_3$), 20.98 (d, $^2J_{CP}$ = 3.6 Hz, ^{i}Pr CH$_3$), 21.70 (s, ^{i}Pr CH$_3$), 32.95 (m, ^{i}Pr CH), 34.81 (m, ^{i}Pr CH), 123.57 (s, Ar-C), 127.48 (s, Ar-C), 127.61 (s, Ar-C), 128.46 (s, Ar-C), 128.52 (s, Ar-C), 129.81 (s, Ar-C), 132.26 (s, Ar-C), 134.03 (s, Ar-C). **^{29}Si DEPT NMR** (119 MHz, THF-d_8): δ –5 ([SiP$_3^{iPr}$]), 358 (μ-Si). **IR** (cm^{-1}): 1820 (m, shoulder), 1771 (s). **Anal. Calcd** for $C_{54}H_{110}BCoFeP_6Si_2$: C, 57.55; H, 9.84. Found: C, 57.27; H, 10.05.

VARIABLE-TEMPERATURE NMR SPECTROSCOPY

Variable-temperature (VT) NMR spectroscopic studies of **1** were conducted using a 500 MHz Bruker Avance II NMR spectrometer. The temperature of the probe was calibrated by measuring the peak-to-peak separation in the ^{1}H NMR spectrum of a sample of neat ethylene glycol or methanol.[6] An 8.6 mM toluene-d_8 solution of **1** was prepared and used as the stock mixture for VT-NMR experiments. Three sample solutions were prepared: "A"- without added DMAP, "B"- with one equiv added DMAP, and "C"- with 10 equiv added DMAP. All samples were filtered through a glass wool plug prior to data collection. Sample tubes were injected into the NMR spectrometer after equilibration had occurred for each temperature step. Samples were allowed to equilibrate for 5 min in the NMR spectrometer prior to data collection at each temperature.

Exchange rate constants were calculated *via* the peak-to-peak separation[7] of the $N(CH_3)_2$ resonances of coordinated and free DMAP for sample B, assuming that the peak-to-peak separation at 194 K represented the stopped-exchange limit. An Eyring plot was generated for the temperature range from 215 K to 256 K. At temperatures above this point, a transition to a second linear region with a different slope was observed. This phenomenon is presumably coupled to the pseudo-rotation of the [BP_3^{iPr}] ligand: from 256 K to 237 K, the $^{31}P\{^1H\}$ NMR spectra of samples A and B resolve from a broad singlet to two distinct resonances in an approximately 1:2 ratio. A plot of ln(*k/T*) v. *T* for the entire temperature range from 215 K to 335 K for sample B is shown in Figure S1, illustrating the non-linear behavior. At temperatures above 335 K, rapid decomposition of sample B was observed, as evidenced by the ^{1}H NMR spectrum, which displays features resulting from multiple intractable species. The Eyring plot for sample B in the temperature range from 194 K to 256 K is shown in Figure S2.

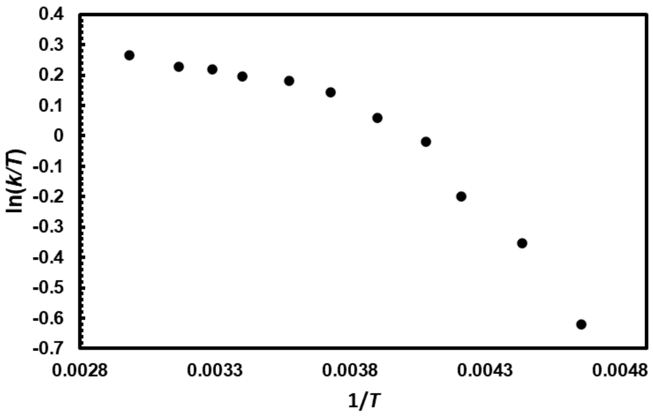

Figure S1. Plot of ln(*k/T*) vs. *T* for sample B (**1** with one equiv DMAP) from 215 K to 335 K.

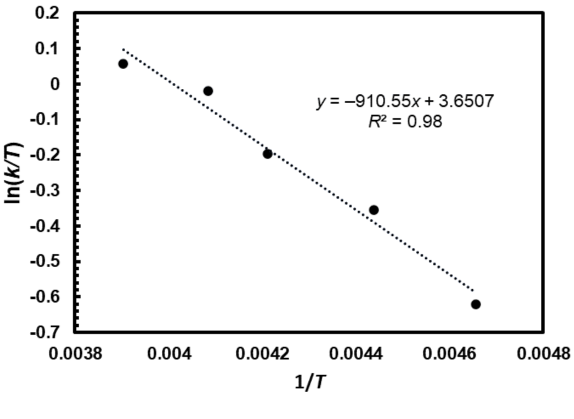

Figure S2. Eyring plot of ln(*k/T*) vs. *T* for sample B (**1** with one equiv DMAP) from 215 K to 256 K.

Table S1. Activation parameters for exchange of DMAP in **1**.

**Calculated thermodynamic parameters**		**Standard uncertainties**	
**Δ*H*‡ (kcal mol^{-1})**	1.8	***σ* (kcal mol^{-1})**	0.2
**Δ*S*‡ (cal mol^{-1}K^{-1})**	–40.0	***σ* (cal mol^{-1}K^{-1})**	0.7
**Δ*G*‡$_{298\ K}$ (kcal mol^{-1})**	13.7	***σ* (kcal mol^{-1})**	0.3

X-RAY CRYSTALLOGRAPHY

Crystalline samples were prepared in a glovebox by decanting residual supernatant and immersing the crystals under a protective layer of Paratone N oil. All samples were frozen in a container of dry ice prior to data collection. Data for **1**, **2, 3**, **5**, **6**, and **7** were collected at the Advanced Light Source beamline 12.2.1 using a Bruker D85 three-circle diffractometer equipped with a PHOTON II CCD area detector using synchrotron radiation (λ = 0.7288 Å) from a Si(111) double crystal Si(111) monochromator. Structures were solved by intrinsic phasing using the SHELXT[8] software package and refined using SHELXL[9] in the OLEX2 interface.[10] Disordered solvent molecules were generally treated with the aid of FragmentDB.[11]

Structure determination of 1.

Complex **1** crystallized with two molecules in the asymmetric unit. Of these, one molecule possesses near total disorder of the [BP_3^{iPr}] ligand. For the disordered [BP_3^{iPr}] ligand, phosphine ^{i}Pr groups are totally positionally disordered, possess disorder of only one methyl group, or are not discernably disordered. All methylene (CH_2) linkers of the diisopropylphosphinomethyl arms of the disordered [BP_3^{iPr}] ligand are disordered. The relative occupancies of the disordered ^{i}Pr groups were refined with free variables. The disordered methylene linkers were grouped into two parts according to the most chemically reasonable positions and their relative occupancies were refined with a free variable. RIGU, SIMU, DELU, and SADI restraints were used to maintain reasonable anisotropic displacement parameters (ADPs) and geometries for the disordered fragments. The EADP constraint was used to maintain reasonable ADPs for nearly overlapping pair of atoms. A second molecule of **1** exhibits no discernable disorder. Only the silicon-bound hydrogen atoms of the non-disordered molecule of **1** could be found in the difference map and refined isotropically. All other hydrogen atoms were placed in calculated positions and refined isotropically.

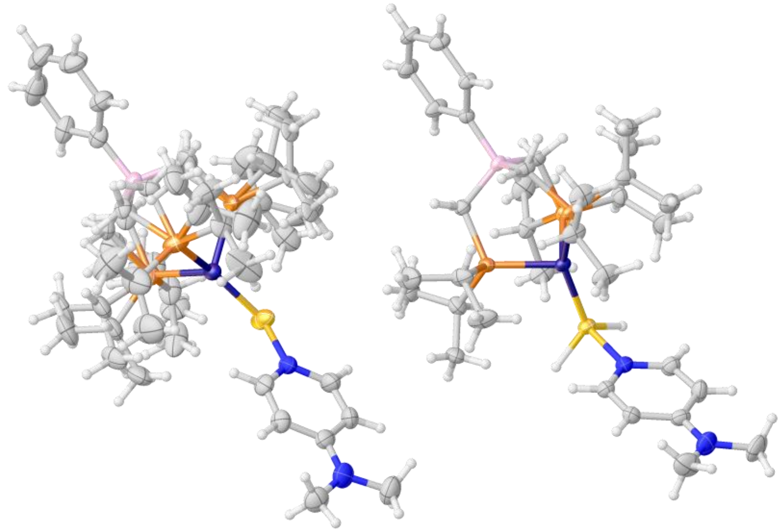

Figure S3. View of the asymmetric unit of **1**. Color scheme: C, light gray; H, white; P, orange; N, blue; B, pink; Co, dark blue.

Table S2. Crystal data and structure refinement for **1**.

Empirical formula	$C_{34}H_{63}BCoN_2P_3Si$	μ/mm^{-1}	0.670
Formula weight	690.60	F(000)	2976.0
Temperature/K	100	Crystal size/mm^3	0.15 × 0.11 × 0.075
Crystal system	monoclinic	Radiation	synchrotron (λ = 0.7288 Å)
Space group	*Cc*	2Θ range for data collection/°	4.4 to 56.672
a/Å	37.054(2)	Index ranges	$-48 \le h \le 48$, $-14 \le k \le 14$, $-26 \le l \le 26$
b/Å	10.8171(7)	Reflections collected	54803
c/Å	20.2804(11)	Independent reflections	17463 [R_{int} = 0.0640, R_{sigma} = 0.0631]
α/°	90	Data/restraints/parameters	17463/72/940
β/°	109.804(2)	Goodness-of-fit on F^2	1.056
γ/°	90	Final R indexes [I>=2σ (I)]	R_1 = 0.0475, wR_2 = 0.1245
Volume/Å^3	7648.0(8)	Final R indexes [all data]	R_1 = 0.0538, wR_2 = 0.1300
Z	8	Largest diff. peak/hole / e Å^{-3}	0.76/–0.47
ρ_{calc}g/cm^3	1.200		
Flack parameter	0.030(13)		

Structure determination of 2.

The solid-state molecular structure of **2** exhibits disorder of one ^{i}Pr group and two-site disorder of the silicon atom. In both cases, the relative occupancies of the disordered components was refined with free variables. Only one of the cobalt-bound hydride ligands (in a bridging position between Co and B) were located in the difference map and refined isotropically. All other hydrogen atoms, including the silicon-bound hydrogen atoms, were placed in calculated positions and refined isotropically.

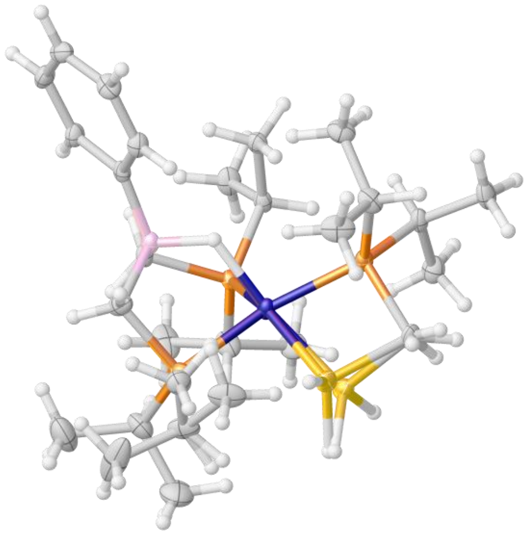

Figure S4. Solid-state molecular structure of **2**. Color scheme: C, light gray; H, white; P, orange; B, pink; Co, dark blue.

Table S3. Crystal data and structure refinement for **2**.

Empirical formula	$C_{27}H_{57}BCoP_3Si$	μ/mm^{-1}	0.785
Formula weight	572.45	F(000)	1236.0
Temperature/K	100	Crystal size/mm^3	0.04 × 0.025 × 0.025
Crystal system	monoclinic	Radiation	synchrotron (λ = 0.7288 Å)
Space group	$P2_1/c$	2Θ range for data collection/°	5.184 to 56.862
a/Å	17.9568(19)	Index ranges	$-23 \le h \le 23$, $-12 \le k \le 12$, $-23 \le l \le 23$
b/Å	9.9954(11)	Reflections collected	44885
c/Å	18.0175(19)	Independent reflections	7370 [R_{int} = 0.0860, R_{sigma} = 0.0613]
α/°	90	Data/restraints/parameters	7370/0/347
β/°	98.480(4)	Goodness-of-fit on F^2	1.056
γ/°	90	Final R indexes [I>=2σ (I)]	R_1 = 0.0435, wR_2 = 0.1064
Volume/Å^3	3198.5(6)	Final R indexes [all data]	R_1 = 0.0553, wR_2 = 0.1112
Z	4	Largest diff. peak/hole / e Å^{-3}	0.53/–0.50
ρ_{calc} g/cm^3	1.187		

Structure determination of 3.

The cobalt-bound hydride ligands of **3** were found in the difference map and refined isotropically. All other hydrogen atoms were placed in calculated positions and refined isotropically.

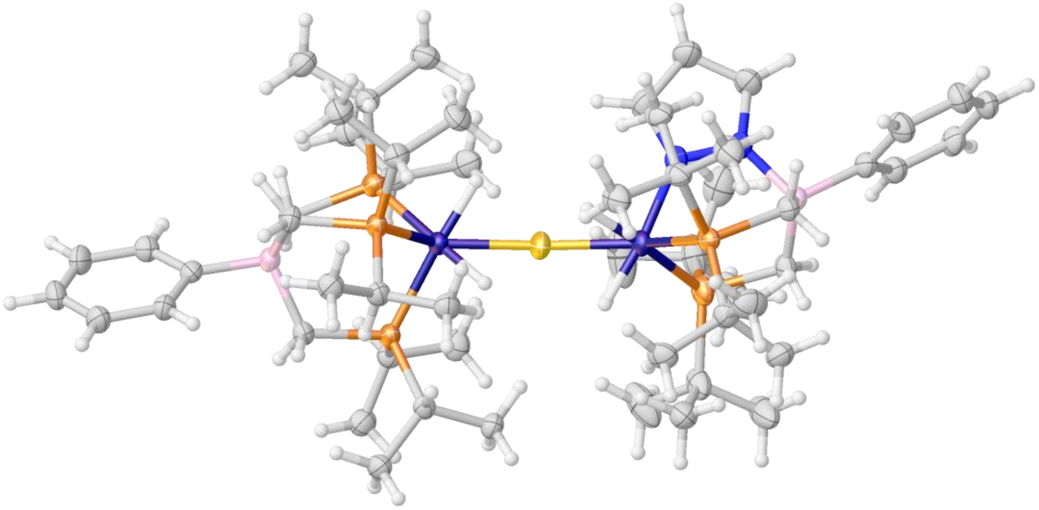

Figure S5. Solid-state molecular structure of **3**. Color scheme: C, light gray; H, white; P, orange; N, blue; B, pink; Co, dark blue.

Table S4. Crystal data and structure refinement for **3**.

Empirical formula	$C_{54}H_{105}B_2Co_2N_2P_5Si$	μ/mm^{-1}	0.812
Formula weight	1104.81	F(000)	1192.0
Temperature/K	100.15	Crystal size/mm^3	0.21 × 0.08 × 0.04
Crystal system	triclinic	Radiation	synchrotron (λ = 0.7288 Å)
Space group	*P*–1	2Θ range for data collection/°	2.658 to 55.332
a/Å	11.3760(8)	Index ranges	$-14 \le h \le 14$, $-21 \le k \le 21$, $-22 \le l \le 22$
b/Å	17.0283(11)	Reflections collected	41587
c/Å	17.8655(12)	Independent reflections	12511 [R_{int} = 0.0533, R_{sigma} = 0.0619]
α/°	108.343(3)	Data/restraints/parameters	12511/0/635
β/°	104.547(3)	Goodness-of-fit on F^2	1.064
γ/°	106.821(3)	Final R indexes [I>=2σ (I)]	R_1 = 0.0520, wR_2 = 0.1359
Volume/Å^3	2912.8(3)	Final R indexes [all data]	R_1 = 0.0735, wR_2 = 0.1455
Z	2	Largest diff. peak/hole / e Å^{-3}	0.85/–0.68
ρ_{calc} g/cm^3	1.260		

Structure determination of 5.

Molecules of **5** occupy a special position such that the asymmetric unit contains half a unit of $[BP_3^{iPr}](H)_2Co=Si=Co(H)_2[BP_3^{iPr}]$. The asymmetric unit contains two molecules of THF, which are each disordered across two positions. The relative occupancies of each pair of disordered components was refined with free variables. RIGU and SADI restraints were used to maintain reasonable anisotropic displacement parameters (ADPs) and geometries for the disordered fragments. The EADP constraint was used to maintain reasonable ADPs for nearly overlapping pair of atoms. The cobalt-bound hydride ligands of **5** were found in the difference map and refined isotropically. All other hydrogen atoms were placed in calculated positions and refined isotropically.

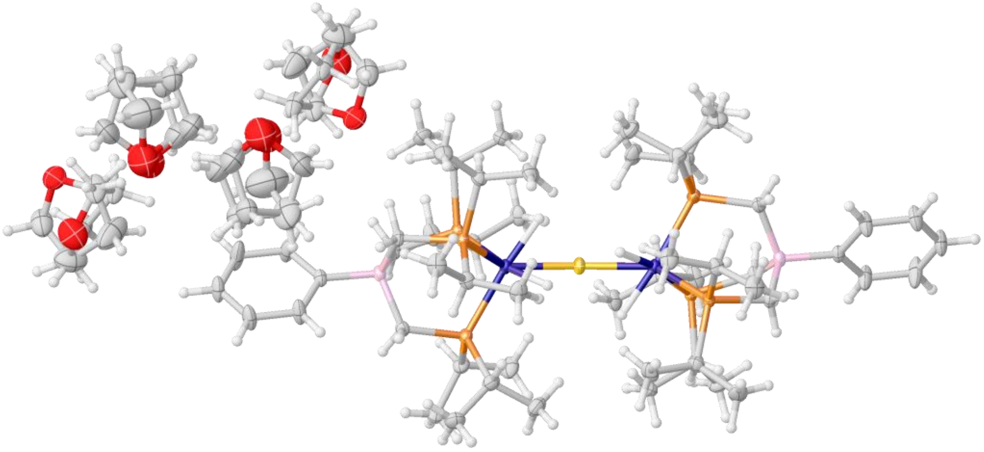

Figure S6. Solid-state molecular structure of **5**. Color scheme: C, light gray; H, white; P, orange; B, pink; Co, dark blue.

Table S5. Crystal data and structure refinement for **5**.

Empirical formula	$C_{66}H_{142}B_2Co_2O_3P_6Si$	μ/mm^{-1}	0.658
Formula weight	1337.18	F(000)	2920.0
Temperature/K	100.15	Crystal size/mm^3	0.22 × 0.21 × 0.17
Crystal system	monoclinic	Radiation	synchrotron (λ = 0.7288 Å)
Space group	*C*2/*c*	2Θ range for data collection/°	5.468 to 62.91
a/Å	26.967(3)	Index ranges	$-38 \leq h \leq 38$, $-22 \leq k \leq 22$, $-31 \leq l \leq 31$
b/Å	15.5581(14)	Reflections collected	64880
c/Å	22.321(3)	Independent reflections	11449 [R_{int} = 0.0676, R_{sigma} = 0.0494]
α/°	90	Data/restraints/parameters	11449/92/451
β/°	125.815(3)	Goodness-of-fit on F^2	1.031
γ/°	90	Final R indexes [I>=2σ (I)]	R_1 = 0.0577, wR_2 = 0.1674
Volume/Å^3	7594.1(15)	Final R indexes [all data]	R_1 = 0.0654, wR_2 = 0.1742
Z	4	Largest diff. peak/hole / e Å^{-3}	1.31/–1.26
ρ_{calc} g/cm^3	1.170		

Structure determination of 6.

Molecules of **6** occupy a special position such that the asymmetric unit contains half a unit of $([BP_3^{iPr}]Co)_2(\mu\text{-}N_2)$. Additionally, the solid-state molecular structure of **6** exhibits disorder of one ^{i}Pr group. The relative occupancies of the disordered components were refined with a free variables. All hydrogen atoms were placed in calculated positions and refined isotropically.

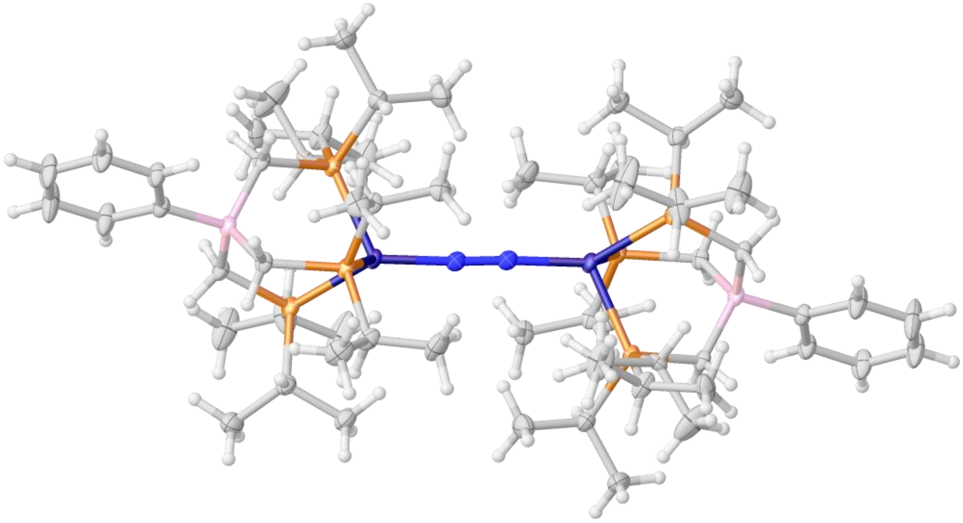

Figure S7. Solid-state molecular structure of **6**. Color scheme: C, light gray; H, white; P, orange; N, blue; B, pink; Co, dark blue.

Table S6. Crystal data and structure refinement for **6**.

Empirical formula	$C_{27}H_{53}BCoNP_3$	μ/mm^{-1}	0.787
Formula weight	554.35	F(000)	1196.0
Temperature/K	100	Crystal size/mm^3	0.08 × 0.05 × 0.04
Crystal system	monoclinic	Radiation	synchrotron (λ = 0.7288 Å)
Space group	$P2_1/n$	2Θ range for data collection/°	4.054 to 62.832
a/Å	13.8254(5)	Index ranges	$-19 \le h \le 19$, $-18 \le k \le 18$, $-23 \le l \le 24$
b/Å	13.0128(5)	Reflections collected	47608
c/Å	16.8771(6)	Independent reflections	9226 [R_{int} = 0.0741, R_{sigma} = 0.0511]
α/°	90	Data/restraints/parameters	9226/0/334
β/°	92.514(2)	Goodness-of-fit on F^2	1.063
γ/°	90	Final R indexes [I>=2σ (I)]	R_1 = 0.0424, wR_2 = 0.0946
Volume/Å^3	3033.39(19)	Final R indexes [all data]	R_1 = 0.0563, wR_2 = 0.1003
Z	4	Largest diff. peak/hole / e Å^{-3}	0.68/–0.66
ρ_{calc} g/cm^3	1.214		

Structure determination of 7.

Molecules of **7** occupy a special position such that the asymmetric unit contains half a unit of $(L(H)_2M)_2(\mu\text{-Si})$ (L = $[BP_3^{iPr}]$, $[SiP_3^{iPr}]$; M = Fe, Co). Because of the space group symmetry and because Fe^0 and Co^I have identical numbers of electrons, the metal sites cannot be distinguished *via* X-ray crystallography. Thus, the metal sites were split into two atoms (Fe and Co) and refined with fixed 0.5 occupancies and EADP constraints. The bridgehead sites of the similarly overlapped $[BP_3^{iPr}]$/$[SiP_3^{iPr}]$ were split into two atoms (B and Si) and refined with fixed 0.5 occupancies and EADP constraints. The asymmetric unit contains two molecules of THF, which are each disordered across two positions. The relative occupancies of each pair of disordered components was refined with free variables. RIGU and SADI restraints were used to maintain reasonable anisotropic displacement parameters (ADPs) and geometries for the disordered fragments. The EADP constraint was used to maintain reasonable ADPs for nearly overlapping pair of atoms. The metal-bound hydride ligands of **7** were found in the difference map and refined isotropically. All other hydrogen atoms were placed in calculated positions and refined isotropically.

Additional structural refinement notes: It is noted that use of a free variable for refinement of the occupancies of the two parts (Co/B and Fe/Si) leads to final occupancies of 0.51 (Co/B) and 0.49 (Fe/Si). If the bridgehead site is instead modeled as a fully occupied boron atom, a non-positive definite atom results; conversely, modelling this site as a fully occupied silicon atom results in an anomalously large U_{eq}.

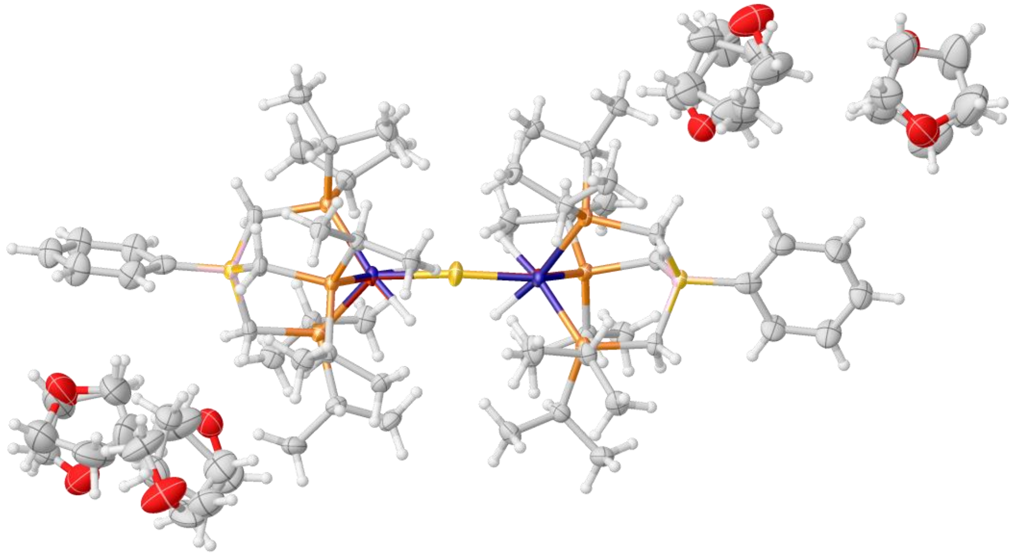

Figure S8. Solid-state molecular structure of **7**. Color scheme: C, light gray; H, white; P, orange; B, pink; Co, dark blue; Iron, orange; O, red.

Table S7. Crystal data and structure refinement for **7**.

Empirical formula	$C_{70}H_{142}BCoFeO_4P_6Si_2$	μ/mm^{-1}	0.643
Formula weight	1415.42	F(000)	3080.0
Temperature/K	100.15	Crystal size/mm^3	0.21 × 0.16 × 0.08
Crystal system	monoclinic	Radiation	synchrotron (λ = 0.7288 Å)
Space group	*C*2/*c*	2Θ range for data collection/°	3.284 to 60.998
a/Å	26.994(3)	Index ranges	–37 ≤ *h* ≤ 37, –21 ≤ *k* ≤ 21, –30 ≤ *l* ≤ 31
b/Å	15.6236(16)	Reflections collected	65259
c/Å	22.459(2)	Independent reflections	10816 [R_{int} = 0.0706, R_{sigma} = 0.0463]
α/°	90	Data/restraints/parameters	10816/215/472
β/°	125.873(3)	Goodness-of-fit on F^2	1.059
γ/°	90	Final R indexes [I>=2σ (I)]	R_1 = 0.0490, wR_2 = 0.1313
Volume/Å^3	7675.4(14)	Final R indexes [all data]	R_1 = 0.0569, wR_2 = 0.1370
Z	4	Largest diff. peak/hole / e Å^{-3}	1.11/–1.00
ρ_{calc}g/cm^3	1.225		

DETAILS OF COMPUTATIONS

General methods: Density functional theory (DFT) calculations were performed in ORCA 5.4[12] and molecular orbitals (MOs) were rendered using ChemCraft.[13] Wiberg bond order (WBI)[14] and atoms in molecules (AIM)[15] analyses were performed using MultiWfn.[16] All geometry optimizations were performed at the ωB97X-D3[17] level of theory; bond lengths were set to standard distances using the "clean" feature within Chemcraft 1.8. The def2-TZVP[18] basis set was used for Co, P, and Si atoms, and the def2-SVP[18] basis set was used for C, H, B, and N atoms in geometry optimizations and frequency calculations. Unless otherwise noted, geometry optimizations used input coordinates adapted from the corresponding single-crystal solid-state molecular structure. The def2-TZVP basis set was used for all atoms in single-point energy calculations. The analytical Hessians of all molecules studied were calculated to verify the optimized structures as local minima on the potential energy surface; small imaginary frequencies (<25 cm^{-1}) are assumed to be the result of the RIJCOSX approximation used in all computations. Natural charges[19] were calculated using the Natural Bond Orbital program (NBO, version 6).[20]

Mechanistic studies: The associative exchange of DMAP from **1** was investigated with a modestly truncated model complex, $[BP_3^{Me}](H)_2CoSiH_2(DMAP)$ ($[BP_3^{Me}] = PhB(CH_2PMe_2)_3^-$; **1***). The input geometry of the *bis*-DMAP intermediate, $[BP_3^{Me}](H)_2CoSiH_2(DMAP)_2$ (**Int***) was adapted from the minimized geometry of **1***: a second molecule of DMAP was brought to a close distance (2.18 Å) of the silicon atom in **1***, opposite to the bound DMAP ligand, and the SiH_2 unit was made to be planar.

The optimized geometries of the **1***+DMAP encounter species were obtained by adapting the optimized geometry of **Int***. Two such encounter species were modeled (Scheme 1), denoted as **1***+**DMAP** and **1*'**+**DMAP**, accounting for the structurally unique products formed when each DMAP is dissociated from **Int**. Transition-states for the pathways **1***+**DMAP→Int** (**TS**) and **1*'+DMAP→Int'** (**TS'**) were located with a nudged-elastic band[21] TS (NEB-TS) search. The approximate geometries of **TS** and **TS'** obtained from the NEB-TS searches were used as inputs for optimizations with analytical Hessians. Once converged, the analytical Hessians of **TS** and **TS'** was calculated again, verifying the presence of an imaginary frequency corresponding to DMAP dissociation. DFT-computed single-point energies were obtained for all species at the ωB97X-D3/def2-TZVP//CPCM[22](toluene) level of theory (Table S8). The reaction coordinate is summarized in Figure 9. Solvated free energies were corrected by subtracting 1.9 kcal mol^{-1} from the free energy of each molecule.[23]

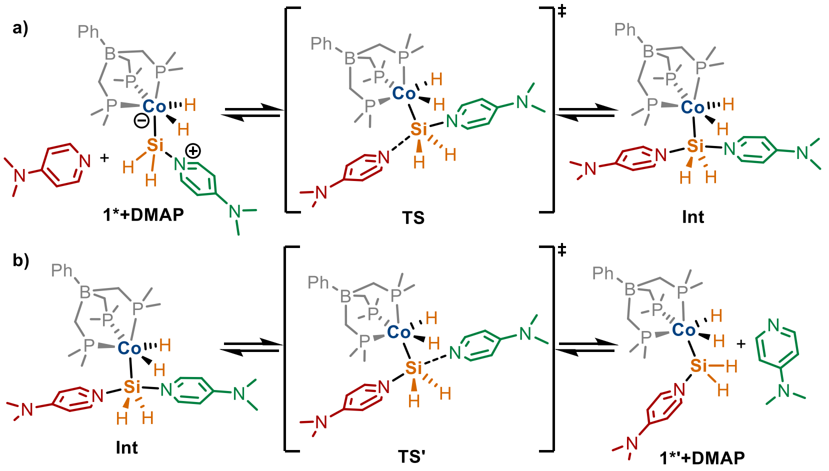

Scheme 1. a) Formation of hypercoordinate adduct **Int** from **1*** + DMAP (**1*** corresponds to the geometry obtained by optimization of the truncated crystallographic molecular structure). b) Dissociation of DMAP from **Int** to generate **1*'**.

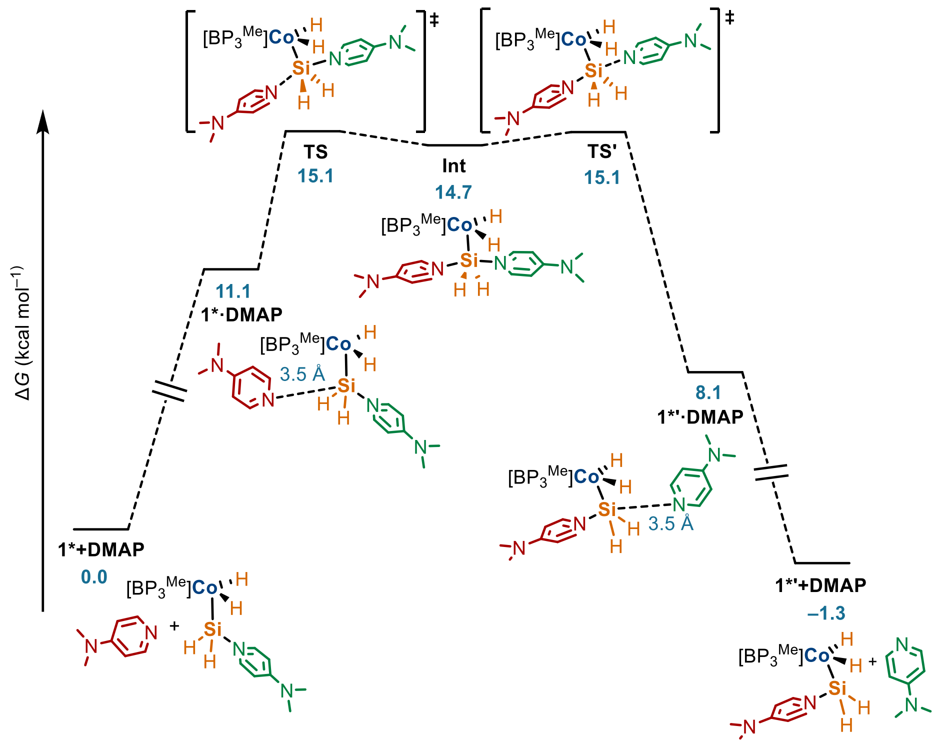

Figure 9. Free-energy reaction coordinate diagram for DMAP exchange in **1***.

Table S8. Tabulated energies and calculated thermochemical quantities for DMAP exchange in **1***.

Species	$E_{el,solv}$ (kcal mol^{-1})[a]	$G–E_{el,solv}$ (kcal mol^{-1})[b]	$G^0 = E_{el,solv} + G$ (kcal mol^{-1})[c]	Δ*G* (kcal mol^{-1})	*H* (kcal mol^{-1})	Δ*H* (kcal mol^{-1})	*S* (cal mol^{-1} K^{-1})	Δ*S* (cal mol^{-1} K^{-1})
**Int**	–2558664.84	443.23	–2558220.96	14.7	–2557224.22	–3.0	256.34	–41.4
**TS**	–2558664.87	443.77	–2558220.59	15.2	–2557224.79	–3.5	251.68	–46.0
**1*·DMAP**	–2558668.64	443.47	–2558224.60	11.2	–2557226.81	–5.6	249.97	–47.8
**1*+DMAP**	–2558664.75	428.43	–2558235.71	0.0	–2557221.26	0.0	297.72	0.0
**1***	–2318776.31	347.00	–2318428.87	—	–2317700.65	—	207.01	—
**TS'**	–2558664.62	443.51	–2558220.59	15.2	–2557224.33	4.8	252.62	–45.1
**1*'·DMAP**	–2558672.06	443.86	–2558227.60	8.1	–2557229.18	–7.9	251.17	–46.5
**1*'+DMAP**	–2558665.75	428.14	–2558237.00	–1.3	–2557221.63	–0.4	301.44	3.7
**1*'**	–2318777.31	346.71	–2318430.16	—	–2317701.02	—	210.74	—
**DMAP**	–239888.44	81.43	–239806.84	—	–239520.61	—	90.70	—

[a] Solvated electronic energy. [b]Thermal correction to $E_{el,\text{solv}}$. [c]Free energy.

Relaxed scan: The energy profile of the conversion of **1*** to **1*'** *via* rotation of the Co—Si bond was modeled with a relaxed scan by varying the P3**—**Co1**—**Si5**—**N9 dihedral angle of **1*** from 135° to 0° (corresponding to the structure of **1*'**) in 10 steps, at the ωB97X-D3/def2-SVP(C,H,B,N),def2-TZVP(Co,Si,P)//CPCM(toluene) level of theory. The rotamer **1*'** is marginally more stable (by 1.1 kcal mol^{-1}) than **1***. Scan energies are summarized in Table S9.

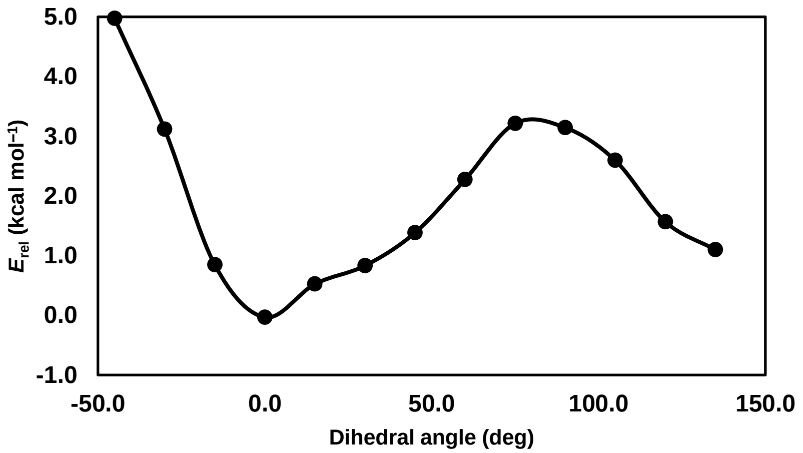

Figure 10. Relaxed scan plot of relative energy v. P3—Co1—Si5—N9 dihedral angle for **1***.

Table S9. Tabulated energies for Co—Si bond rotation from **1*** to produce **1*'**.

**P3—Co1—Si5—N9 Dihedral angle** (deg)	E_{rel} (kcal mol^{-1})
135.0 (**1***)	1.1
120.0	1.6
105.0	2.6
90.0	3.1
75.0	3.2
60.0	2.3
45.0	1.4
30.0	0.8
15.0	0.5
0.0 (**1*'**)	0.0
–15.0	0.8
–30.0	3.1
–45.0	5.0

Table S10. Tabulated WBIs for **1***.

Atom	Atom 2	WBI	Notes
Co1	Si5	1.30	
Si5	H6	0.88	H6 = Si-bound H
Si5	H7	0.89	H7 = Si-bound H
Co1	H72	0.62	H72 = Co-bound H
Co1	H73	0.61	H73= Co-bound H
Si5	H72	0.22	
Si5	H73	0.23	

Table S11. Tabulated natural charges for **1***.

Atom	Natural charge *q*	Notes
Co1	–0.30	
Si5	+0.52	
H6	–0.08	H6 = Si-bound H
H7	–0.09	H7 = Si-bound H
H72	–0.12	H72 = Co-bound H
H73	–0.09	H73= Co-bound H

Table S12. Tabulated WBIs for **3***.

Atom 1	Atom 2	WBI	Notes
Co1	Si8	1.44	Co1 = [BP_3^{iPr}]-side
Co2	Si8	1.48	Co2 = [BP_2^{tBu}Pz]-side
Si8	H169	0.40	H169 = Co1-bound H
Si8	H171	0.42	H171 = Co1-bound H
Si8	H168	0.40	H168 = Co2-bound H
Si8	H170	0.45	H170 = Co2-bound H
Co1	H169	0.50	
Co1	H171	0.47	
Co2	H168	0.53	
Co2	H170	0.47	

Table S13. Tabulated natural charges for **3***.

Atom	Natural charge *q*	Notes
Co1	–0.30	
Co2	0.00	
Si8	+0.52	
H168	–0.08	H168 = Co2-bound H
H169	–0.09	H169 = Co1-bound H
H170	–0.12	H170 = Co2-bound H
H171	–0.09	H171 = Co1-bound H

Table S14. Tabulated WBIs for **5*.**

Atom 1	Atom 2	WBI	Notes
Co1	Si5	1.44	
Co91	Si5	1.44	
Si5	H87	0.42	H87 = Co1-bound H
Si5	H88	0.39	H88 = Co1-bound H
Si5	H89	0.42	H89 = Co91 bound H
Si5	H90	0.40	H90 = Co91 bound H
Co1	H87	0.48	
Co1	H88	0.50	
Co91	H89	0.48	
Co91	H90	0.50	

Table S15. Tabulated natural charges for **5***.

Atom	Natural charge *q*	Notes
Co1	–0.31	
Co91	–0.31	
Si5	0.50	
H87	–0.10	H87 = Co1-bound H
H88	–0.09	H88 = Co1-bound H
H89	–0.10	H89 = Co91 bound H
H90	–0.09	H90 = Co91 bound H

Table S16. Tabulated WBIs for **7***.

Atom 1	Atom 2	WBI	Notes
Co1	Si171	1.40	
Fe2	Si171	1.56	
Si171	H174	0.38	H174 = Co1-bound H
Si171	H175	0.38	H175 = Co1-bound H
Si171	H172	0.40	H172 = Fe2-bound H
Si171	H173	0.41	H173 = Fe2-bound H
Co1	H174	0.51	
Co1	H175	0.52	
Fe2	H172	0.49	
Fe2	H173	0.48	

Table S17. Tabulated natural charges for **7***.

Atom	Natural charge *q*	Notes
Co1	–0.34	
Fe2	–0.88	
Si171	0.53	
H172	–0.09	H172 = Fe2-bound H
H173	–-0.08	H173 = Fe2-bound H
H174	–0.09	H174 = Co1-bound H
H175	–0.10	H175 = Co1-bound H

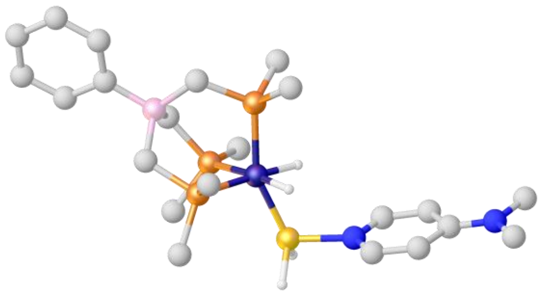

Figure S11. Optimized structure of **1***. Most hydrogen atoms have been omitted for clarity. Color scheme: C, light gray; H, white; P, orange; N, blue; B, pink; Co, dark blue; Si, yellow.

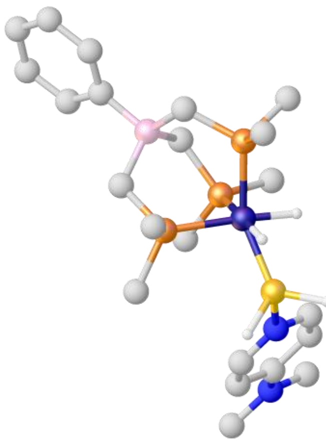

Figure S12. Optimized structure of **1*’**. Most hydrogen atoms have been omitted for clarity. Color scheme: C, light gray; H, white; P, orange; N, blue; B, pink; Co, dark blue; Si, yellow.

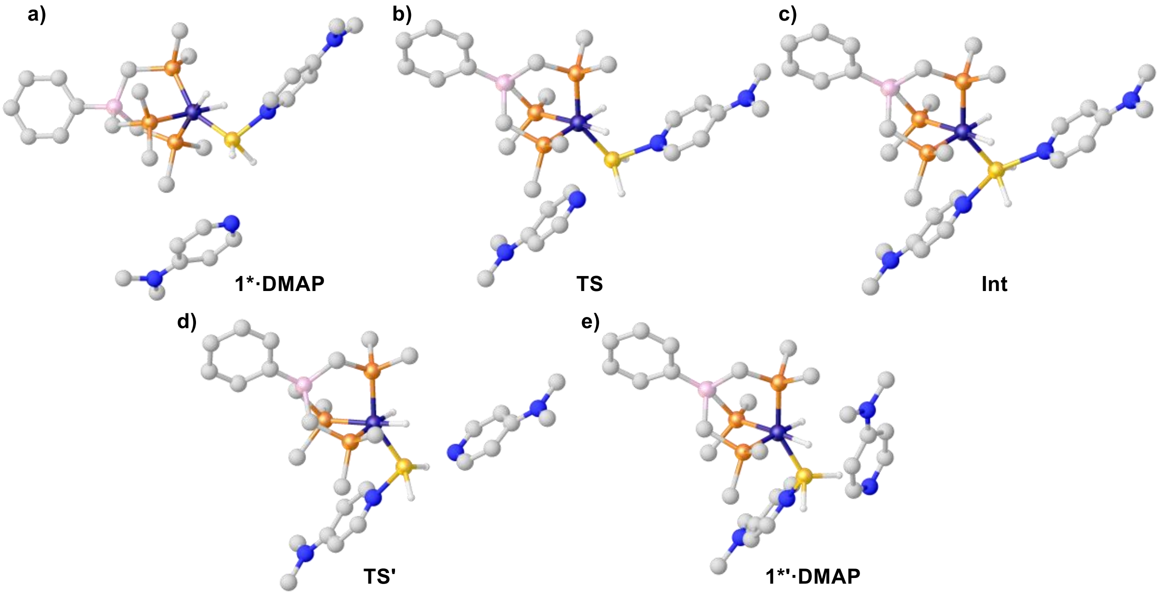

Figure S13. Optimized structures of a) **1*·DMAP**, b) **TS**, c) **Int**, d) **TS’**, and e) **1*’·DMAP**. Most hydrogen atoms have been omitted for clarity. Color scheme: C, light gray; H, white; P, orange; N, blue; B, pink; Co, dark blue; Si, yellow.

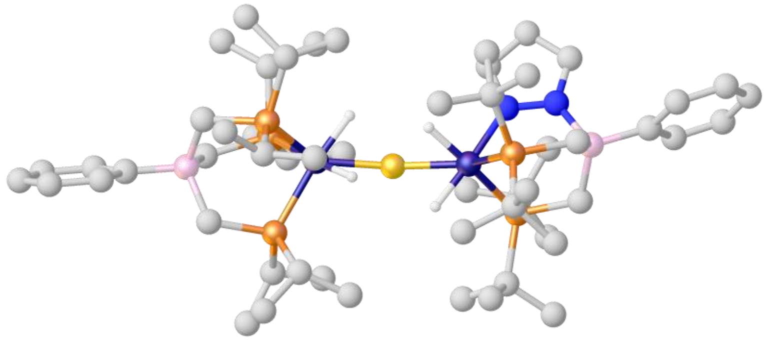

Figure S14. Optimized structure of **3***. Most hydrogen atoms have been omitted for clarity. Color scheme: C, light gray; H, white; P, orange; N, blue; B, pink; Co, dark blue; Si, yellow.

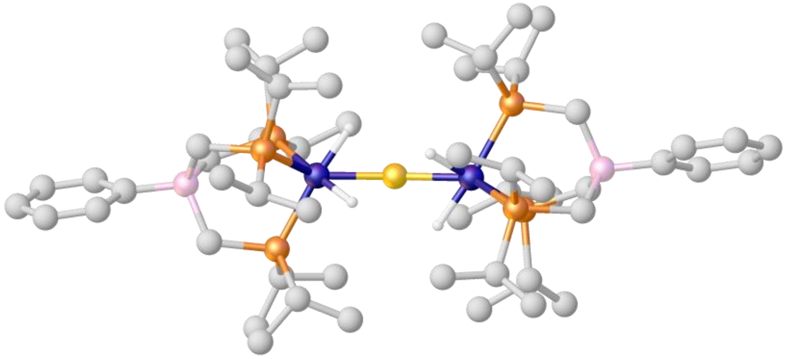

Figure S15. Optimized structure of **5***. Most hydrogen atoms have been omitted for clarity. Color scheme: C, light gray; H, white; P, orange; B, pink; Co, dark blue; Si, yellow.

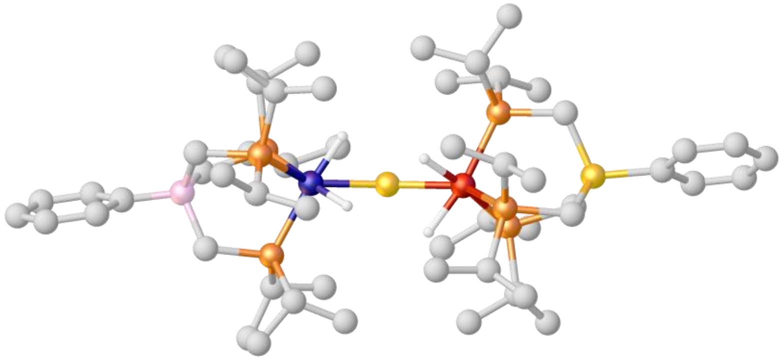

Figure S16. Optimized structure of **7***. Most hydrogen atoms have been omitted for clarity. Color scheme: C, light gray; H, white; P, orange; B, pink; Co, dark blue; Fe, red; Si, yellow.

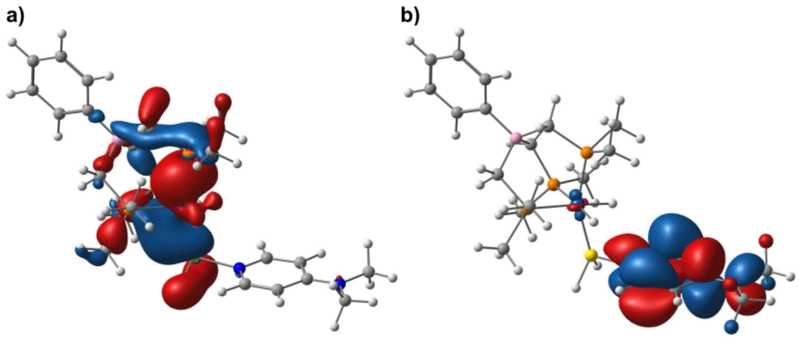

Figure S17. Canonical molecular orbitals of **1***. a) HOMO and b) LUMO depicted at an isovalue of 0.03 *e* Å^{-3}. Most hydrogen atoms have been omitted for clarity. Color scheme: C, light gray; H, white; P, orange; N, blue; B, pink; Co, dark blue; Si, yellow.

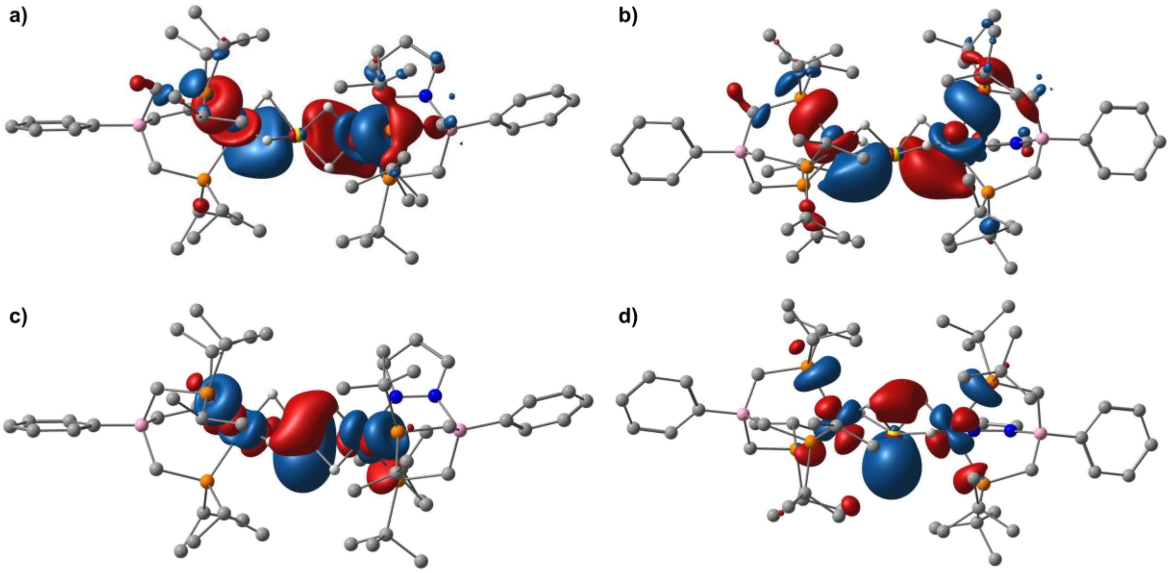

Figure S18. Canonical molecular orbitals of **3***. a) HOMO; b) HOMO, alternate view; c) LUMO; d) LUMO, alternate view, depicted at an isovalue of 0.03 *e* Å^{-3}. Most hydrogen atoms have been omitted for clarity. Color scheme: C, light gray; H, white; P, orange; N, blue; B, pink; Co, dark blue; Si, yellow. The molecular orientation in panes a) and c) is identical to that shown in Figure S14.

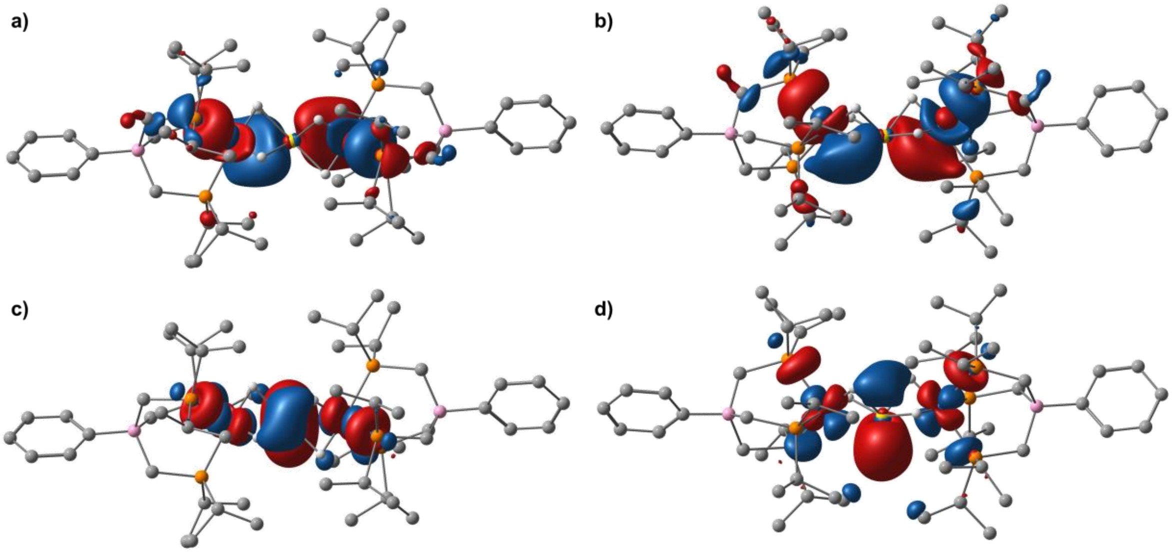

Figure S19. Canonical molecular orbitals of **5***. a) HOMO; b) HOMO, alternate view; c) LUMO; d) LUMO, alternate view, depicted at an isovalue of 0.03 *e* Å^{-3}. Most hydrogen atoms have been omitted for clarity. Color scheme: C, light gray; H, white; P, orange; B, pink; Co, dark blue; Si, yellow. The molecular orientation in panes a) and c) is identical to that shown in Figure S15.

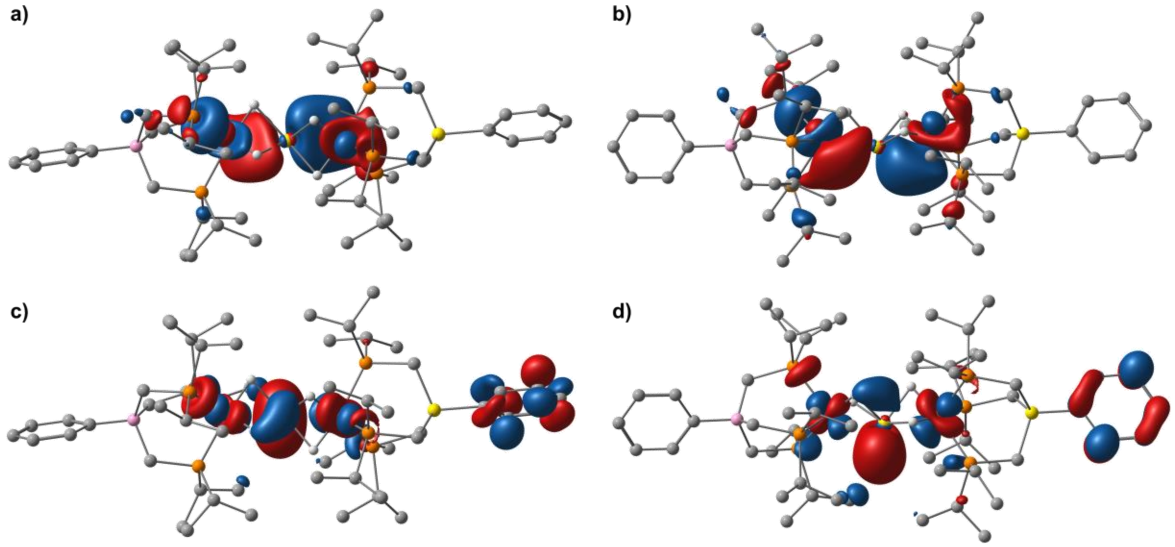

Figure S20. Canonical molecular orbitals of **7***. a) HOMO; b) HOMO, alternate view; c) LUMO+1; d) LUMO+1, alternate view, depicted at an isovalue of 0.03 *e* Å^{-3}. Note: the LUMO of **7*** is of predominantly phenyl antibonding character. Most hydrogen atoms have been omitted for clarity. Color scheme: C, light gray; H, white; P, orange; B, pink; Co, dark blue; Fe, red; Si, yellow. The molecular orientation in panes a) and c) is identical to that shown in Figure S16.

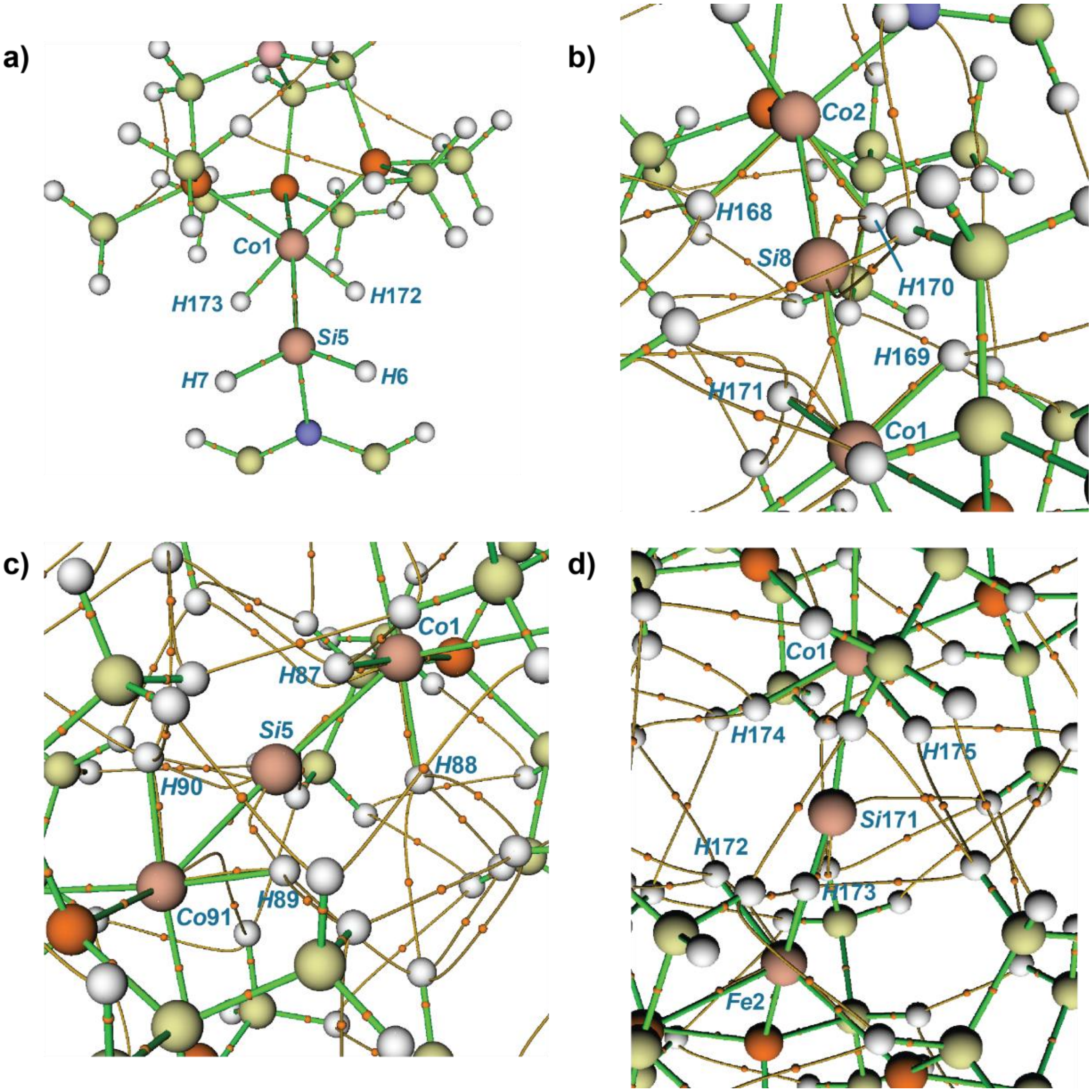

Figure S21. Visualization of the AIM bond paths (depicted as orange curves) within {$M_{1,2}Si$} cores of a) **1**, b) **3**, c) **5**, and d) **7**. Bond critical points are denoted by orange spheres.

UV-VISIBLE SPECTROSCOPY

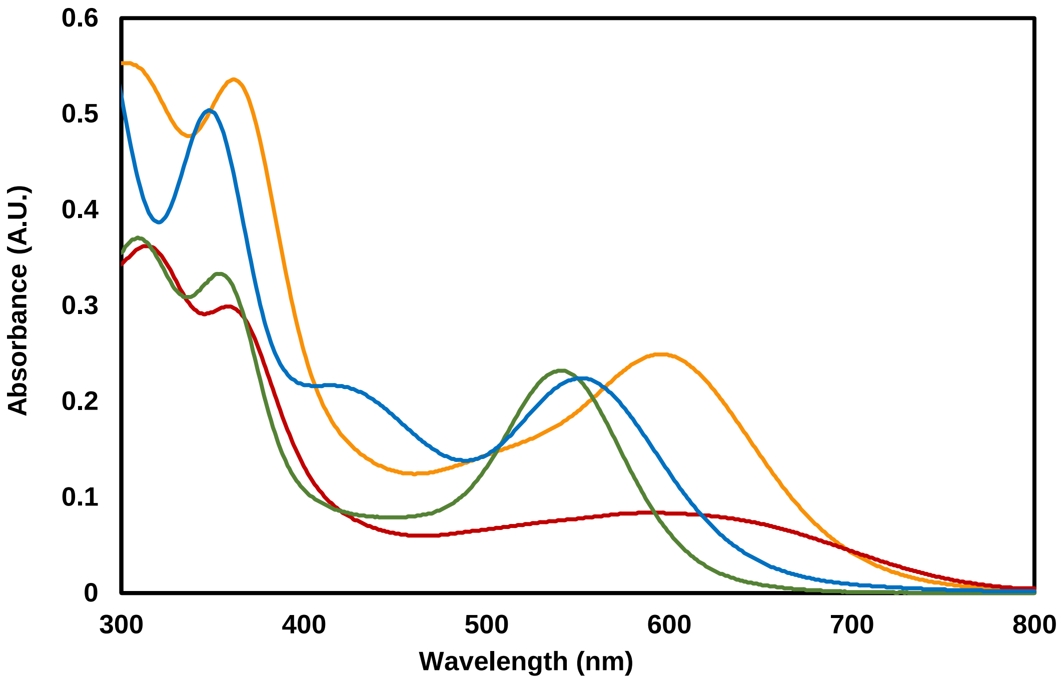

Figure S22. UV-visible spectra of **3** (orange trace, —), **4** (red trace, —), **5** (green trace, —), and **7** (blue trace, —) recorded in toluene solution.

Table 18. Tabulated absorption maxima and extinction coefficients for **3**, **4**, **5**, and **7** for spectra recorded in toluene solution.

**3**		**4**		**5**		**7**	
c (nM)	0.02715	*c* (nM)	0.02735	*c* (nM)	0.02696	*c* (nM)	0.02662
λ_{max} (nm)	ε (M^{-1} cm^{-1})	λ_{max} (nm)	ε (M^{-1} cm^{-1})	λ_{max} (nm)	ε (M^{-1} cm^{-1})	λ_{max} (nm)	ε (M^{-1} cm^{-1})
595	9171	590	3071	541	8606	553	8415
507	5488	359	10933	354	12353	415	14840
362	19741	314	13237	309	13763	348	18935
304	20367						

NMR SPECTROSCOPY

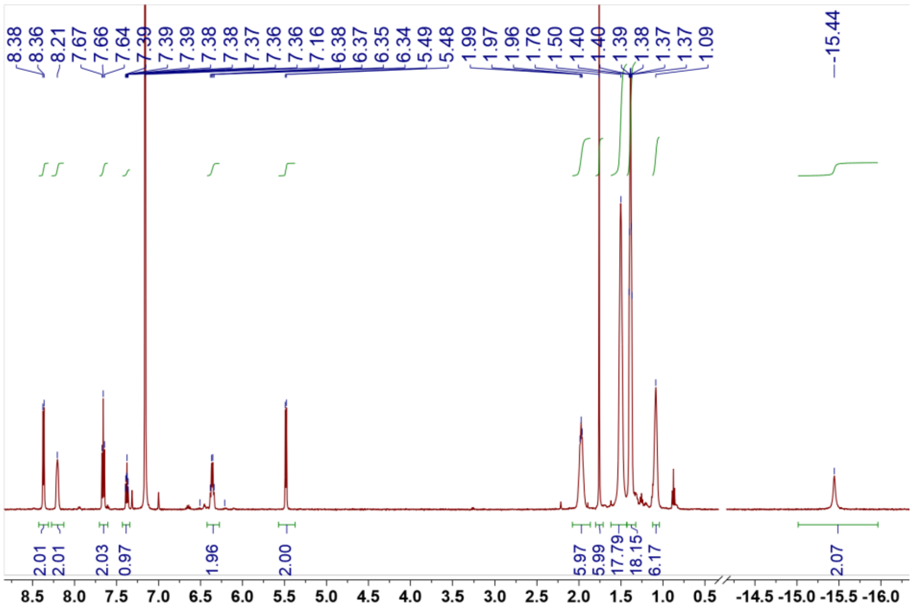

Figure S23. ^{1}H NMR spectrum (500 MHz, benzene-d_6) of **1**.

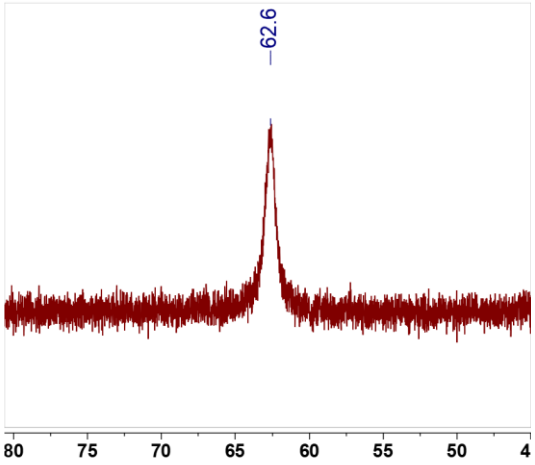

Figure S24. $^{31}P\{^{1}H\}$ NMR spectrum (243 MHz, benzene-d_6) of **1**.

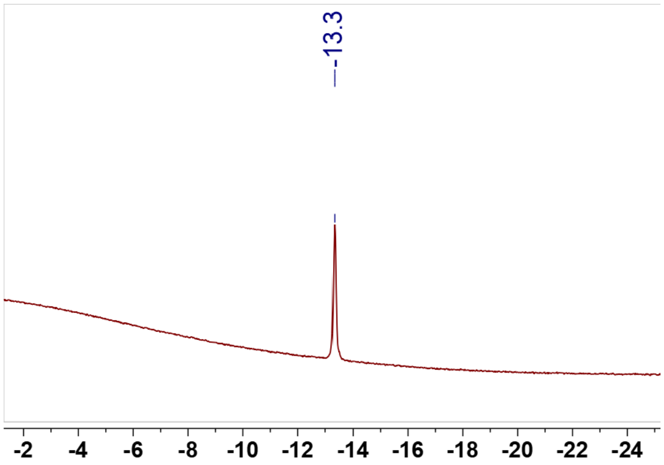

Figure S25. $^{11}B\{^{1}H\}$ NMR spectrum (193 MHz, benzene-d_6) of **1**.

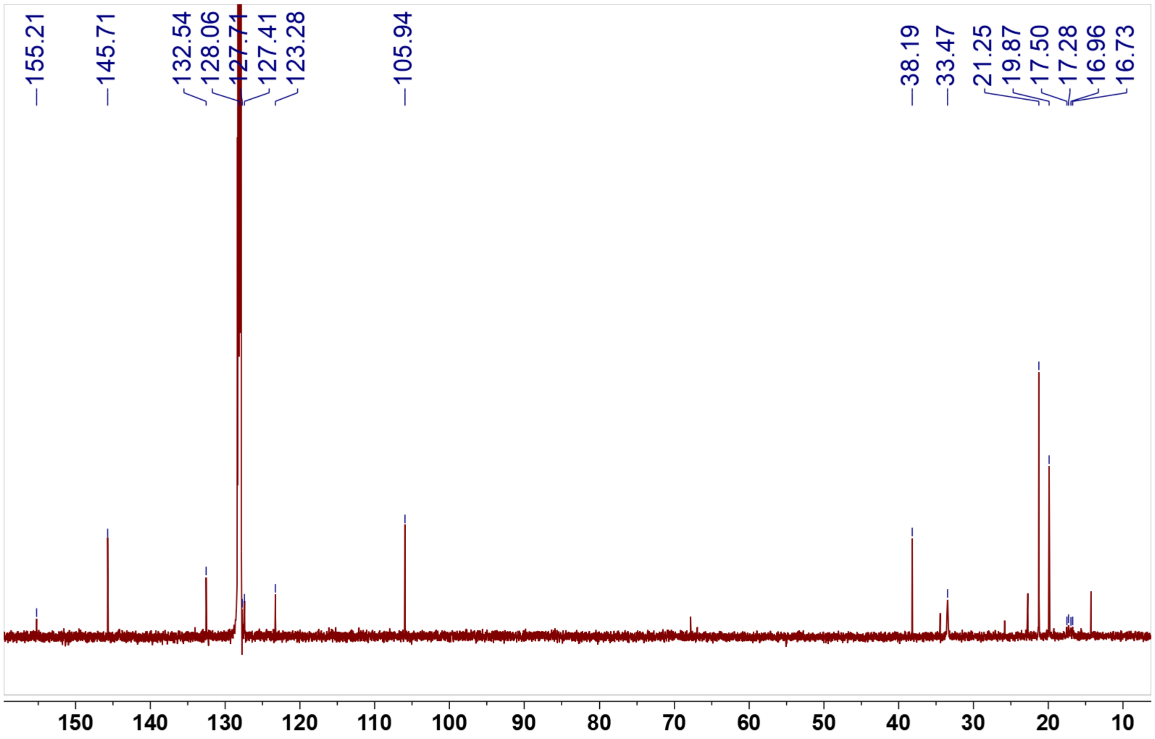

Figure S26. $^{13}C\{^{1}H\}$ NMR spectrum (151 MHz, benzene-d_6) of **1**.

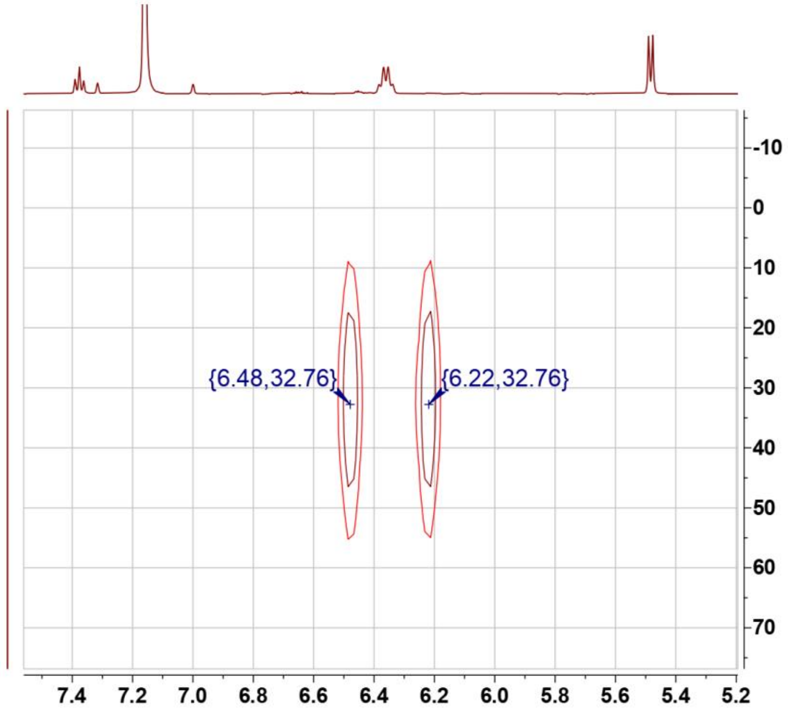

Figure S27. ^{29}Si—^{1}H HMBC NMR spectrum (119 MHz, 600 MHz, benzene-d_6) of **1**.

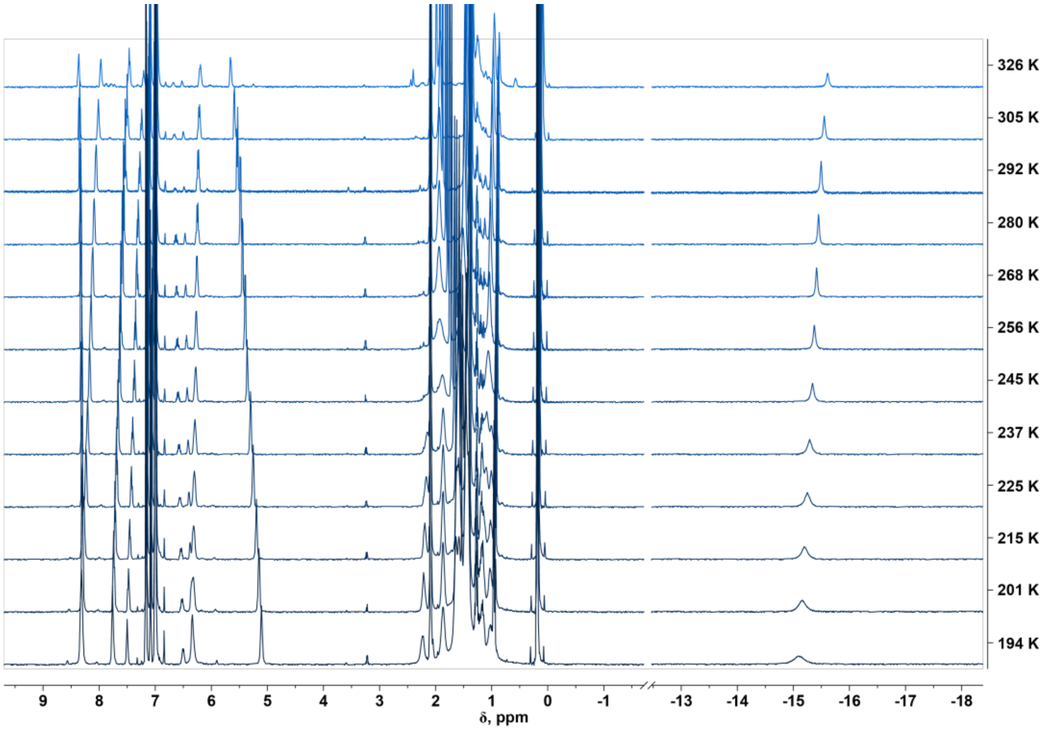

Figure S28. Variable-temperature ^{1}H NMR spectra (500 MHz, toluene-d_8) of **1**.

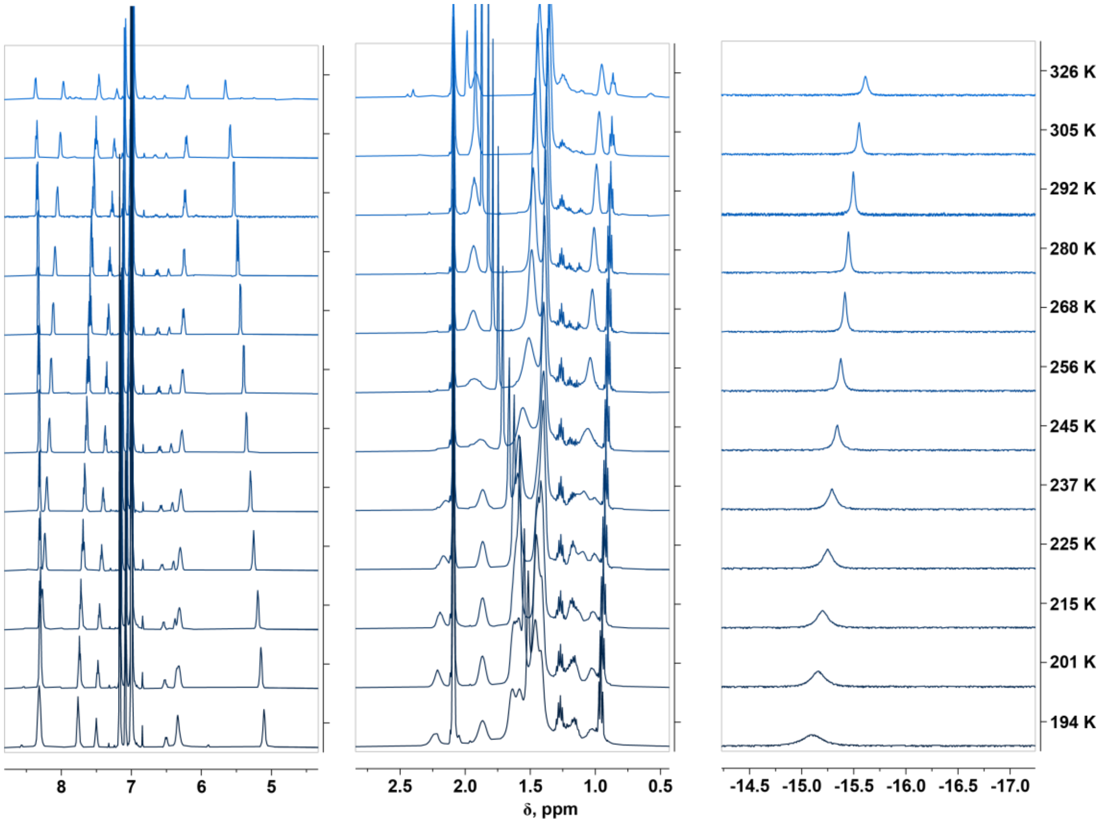

Figure S29. Variable-temperature ^{1}H NMR spectra (500 MHz, toluene-d_8) of **1**, showing magnifications of several spectral regions. Note that each region is independently scaled to provide greatest clarity of the spectroscopic features.

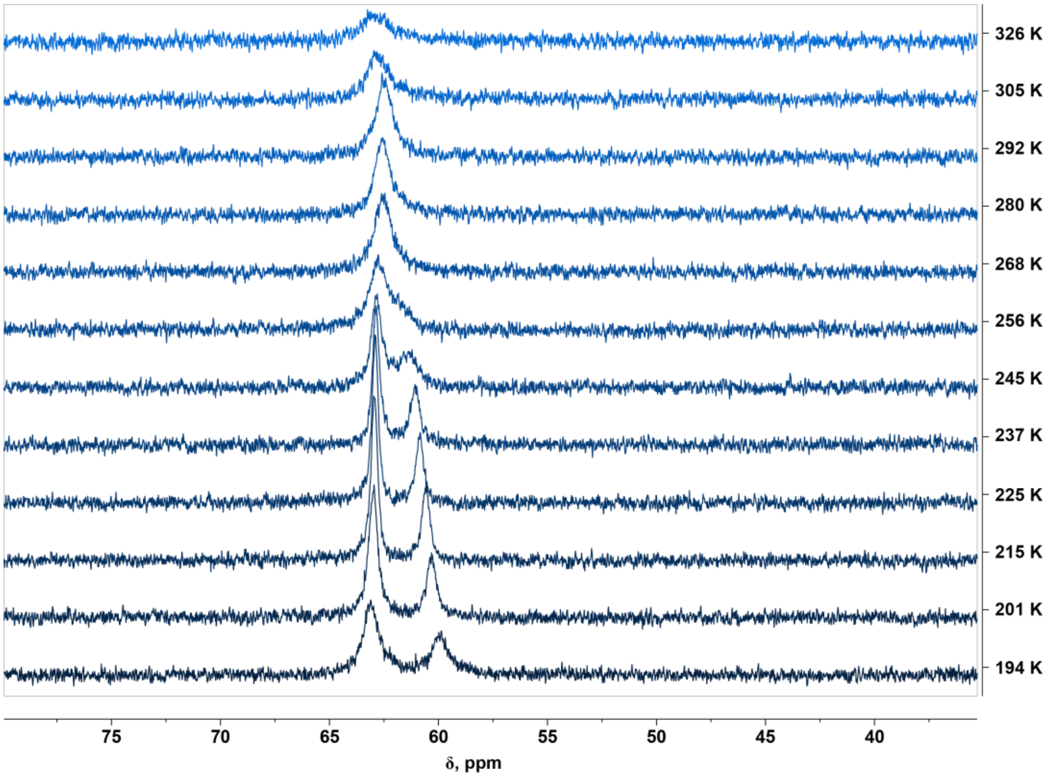

Figure S30. Variable-temperature $^{31}P\{^{1}H\}$ NMR spectra (202 MHz, toluene-d_8) of **1**.

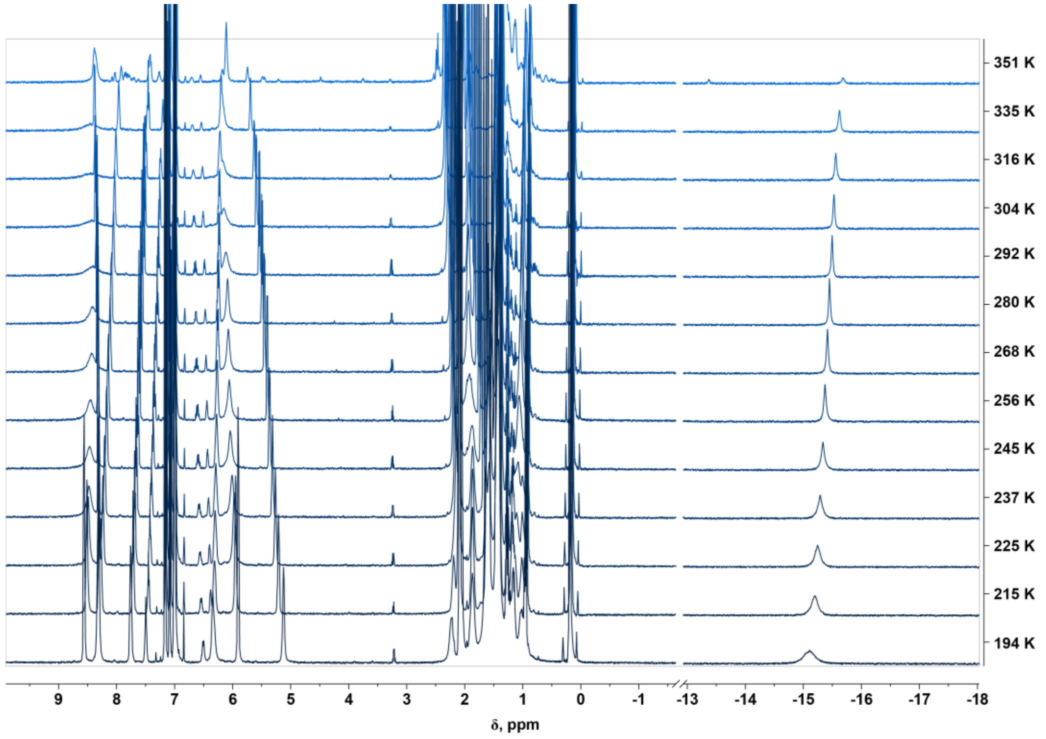

Figure S31. Variable-temperature ^{1}H NMR spectra (500 MHz, toluene-d_8) of **1** in the presence of one equiv of added DMAP. Decomposition of **1** is apparent at 351 K.

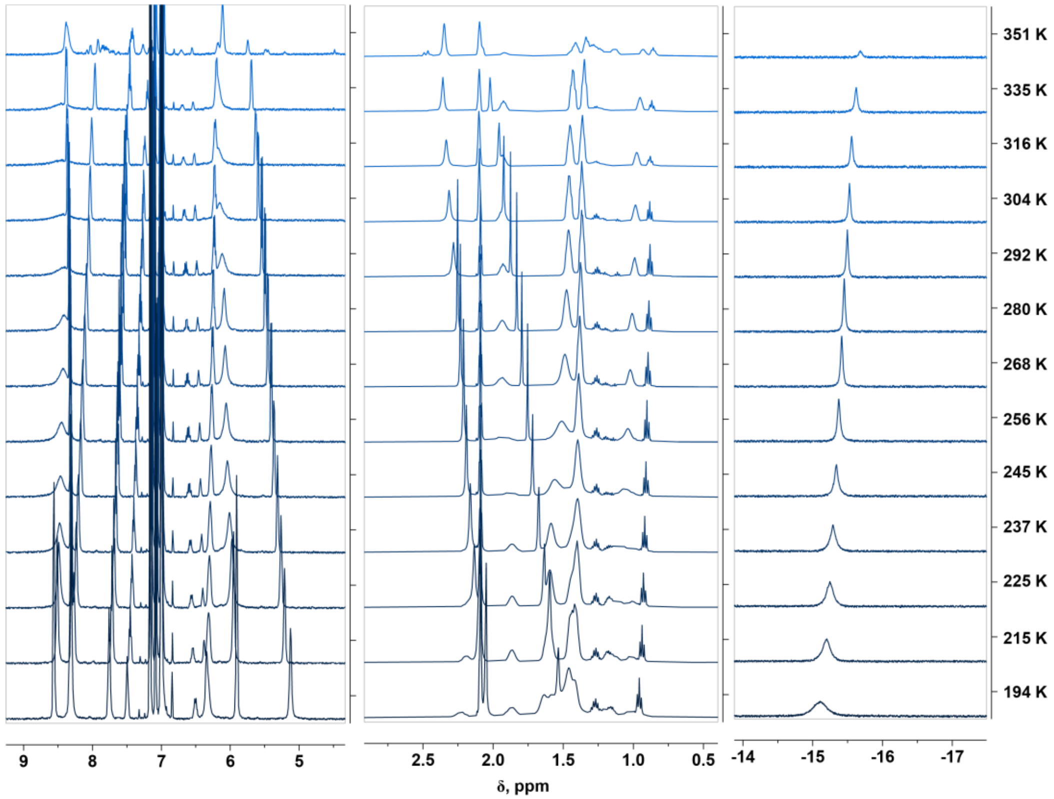

Figure S32. Variable-temperature ^{1}H NMR spectra (500 MHz, toluene-*d*8) of **1** in the presence of one equiv of added DMAP, showing magnifications of several spectral regions. Note that each region is independently scaled to provide greatest clarity of the spectroscopic features.

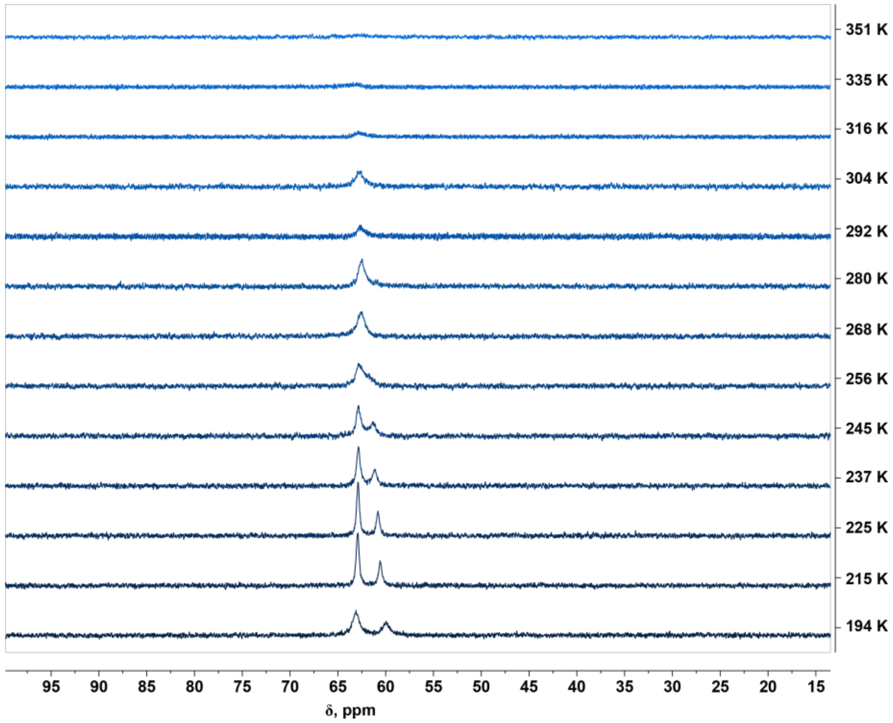

Figure S33. Variable-temperature $^{31}P\{^{1}H\}$ NMR spectra (202 MHz, toluene-*d*8) of **1** in the presence of one equiv of added DMAP.

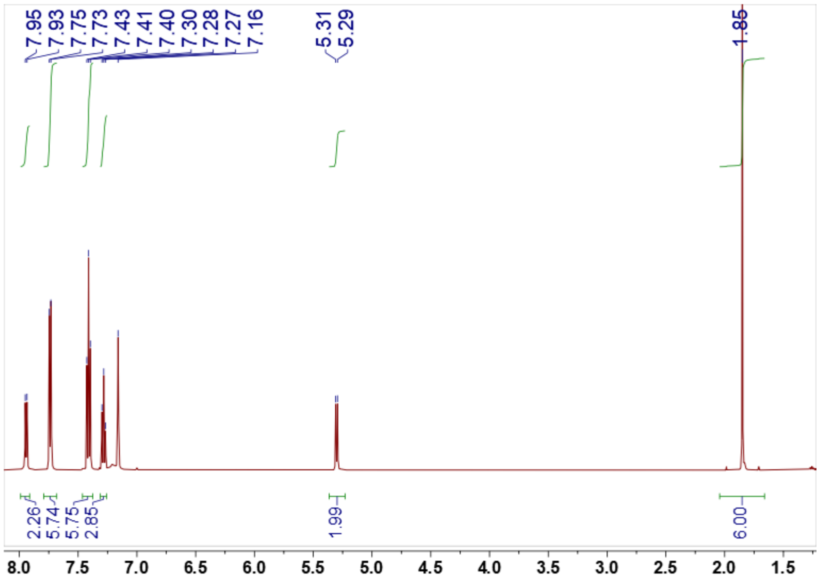

Figure S34. ^{1}H NMR spectrum (500 MHz, benzene-d_6) of Ph_3B-DMAP.

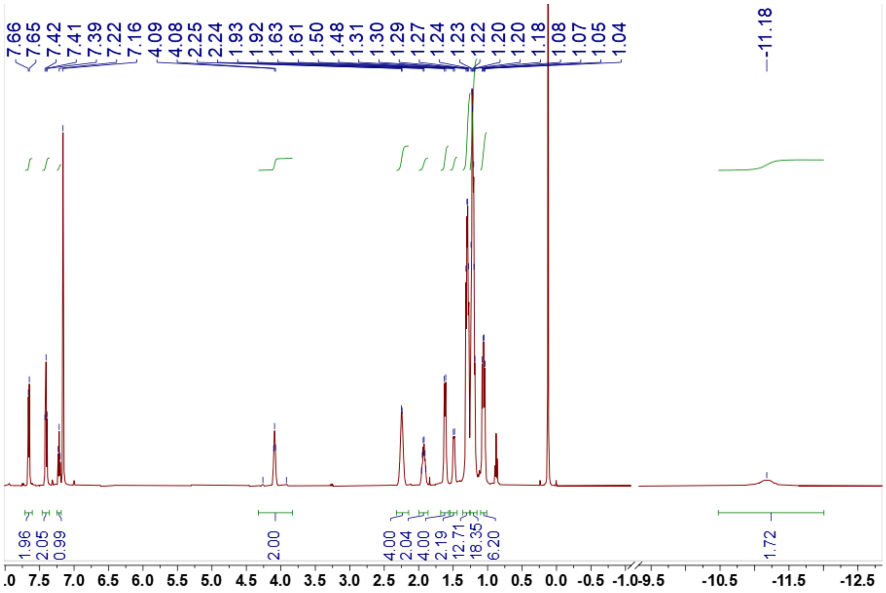

Figure S35. ^{1}H NMR spectrum (500 MHz, benzene-d_6) of **2**.

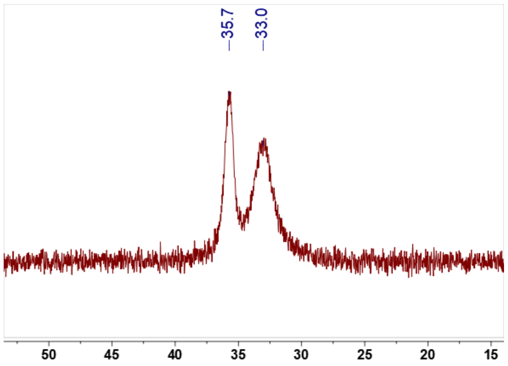

Figure S36. ^{31}P{^{1}H} NMR spectrum (203 MHz, benzene-d_6) of **2**.

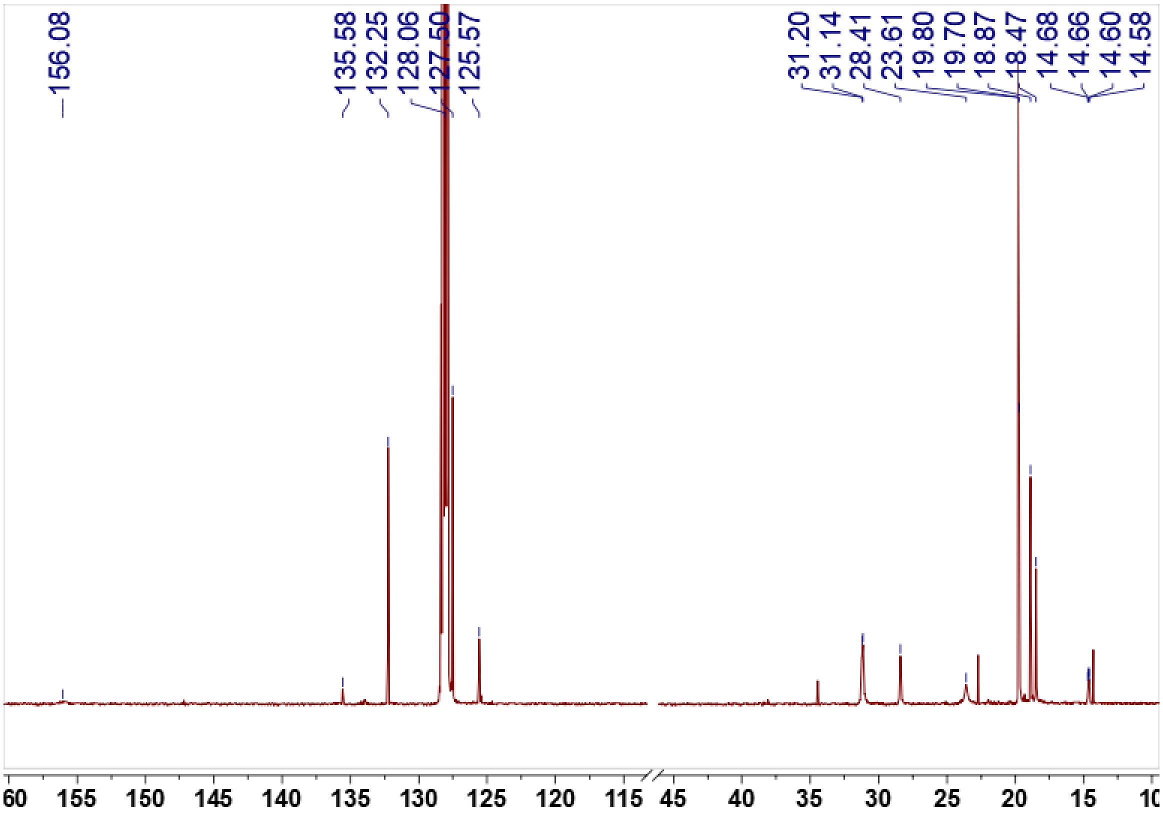

Figure S37. $^{13}C\{^1H\}$ NMR spectrum (151 MHz, benzene-d_6) of **2**. The chemical shifts of resonances due to adventitious *n*-pentane[4] are not labeled.

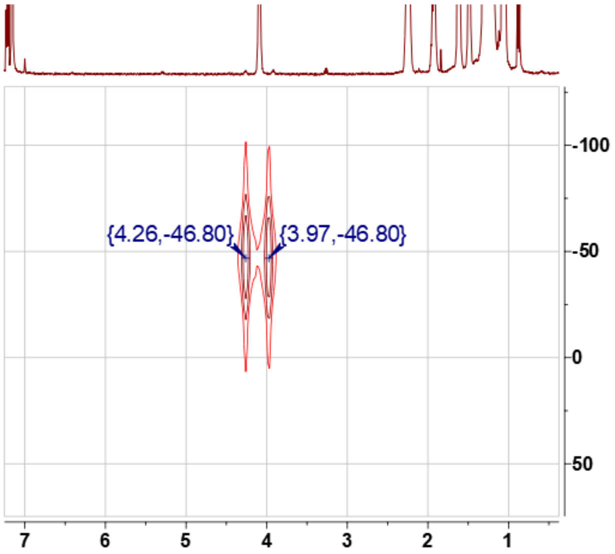

Figure S38. ^{29}Si—^1H HMBC NMR spectrum (119 MHz, 600 MHz, benzene-d_6) of **2**.

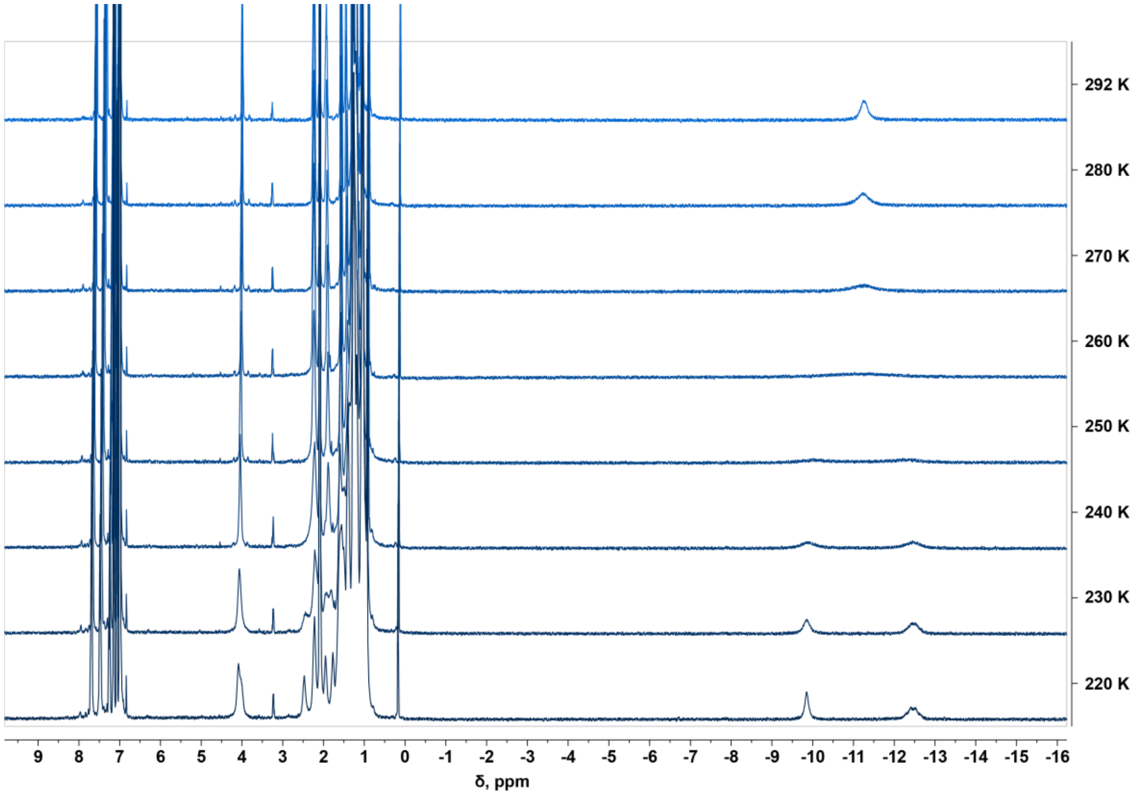

Figure S39. Variable-temperature ^{1}H NMR spectra (500 MHz, toluene-d_8) of **2**.

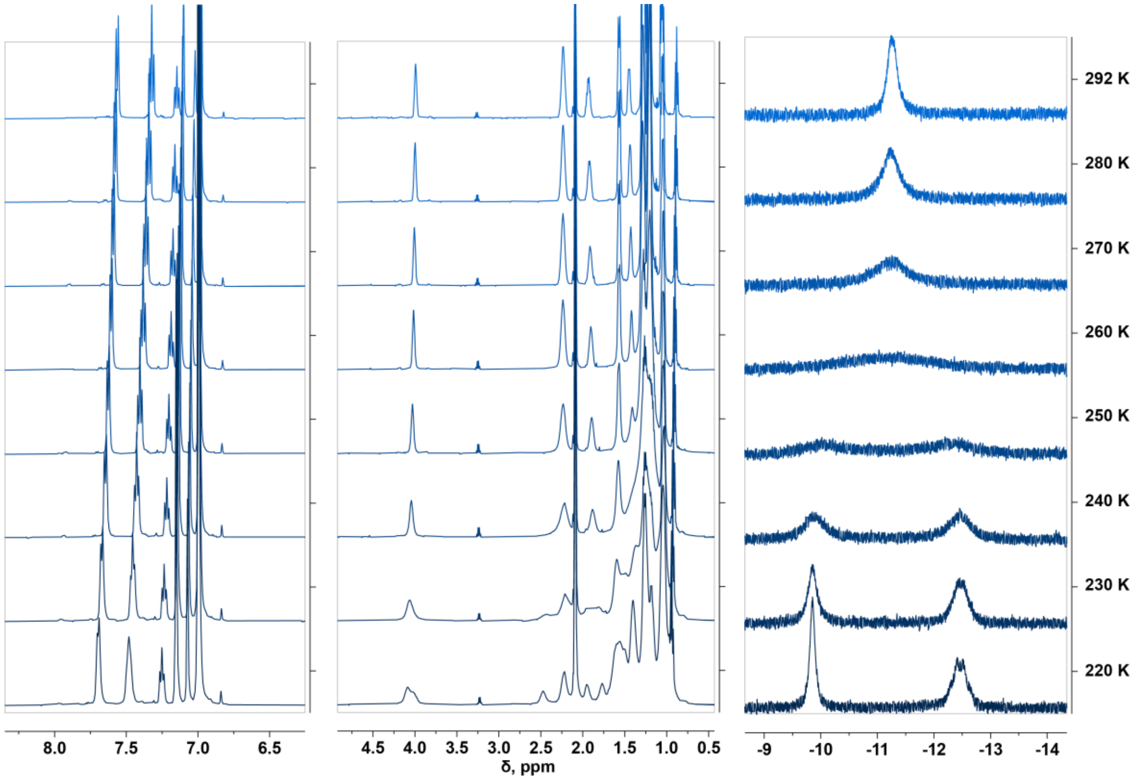

Figure S40. Variable-temperature ^{1}H NMR spectra (500 MHz, toluene-d_8) of **2**, showing magnifications of several spectral regions. Note that each region is independently scaled to provide greatest clarity of the spectroscopic features.

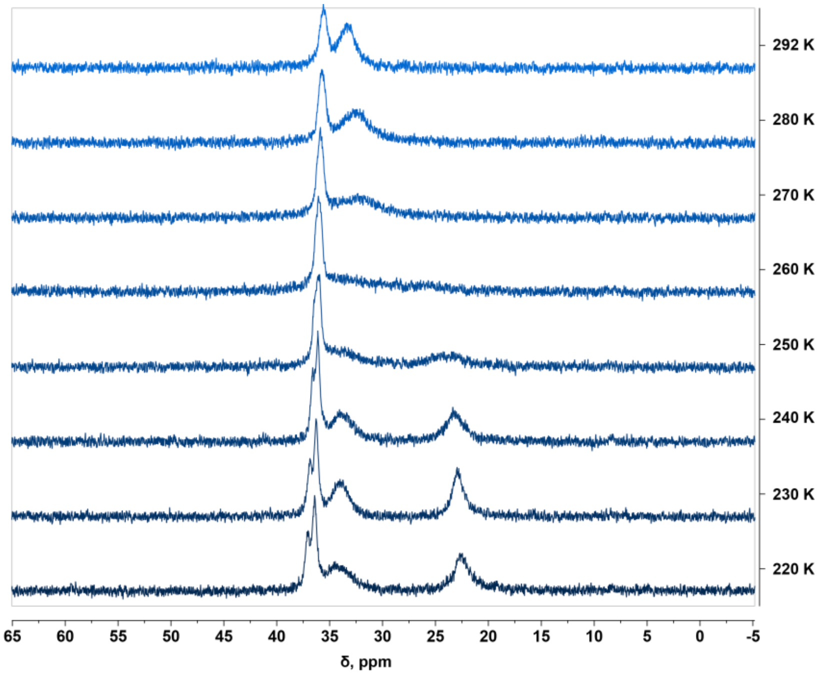

Figure S41. Variable-temperature $^{31}P\{^1H\}$ NMR spectra (202 MHz, toluene-d_8) of **2**.

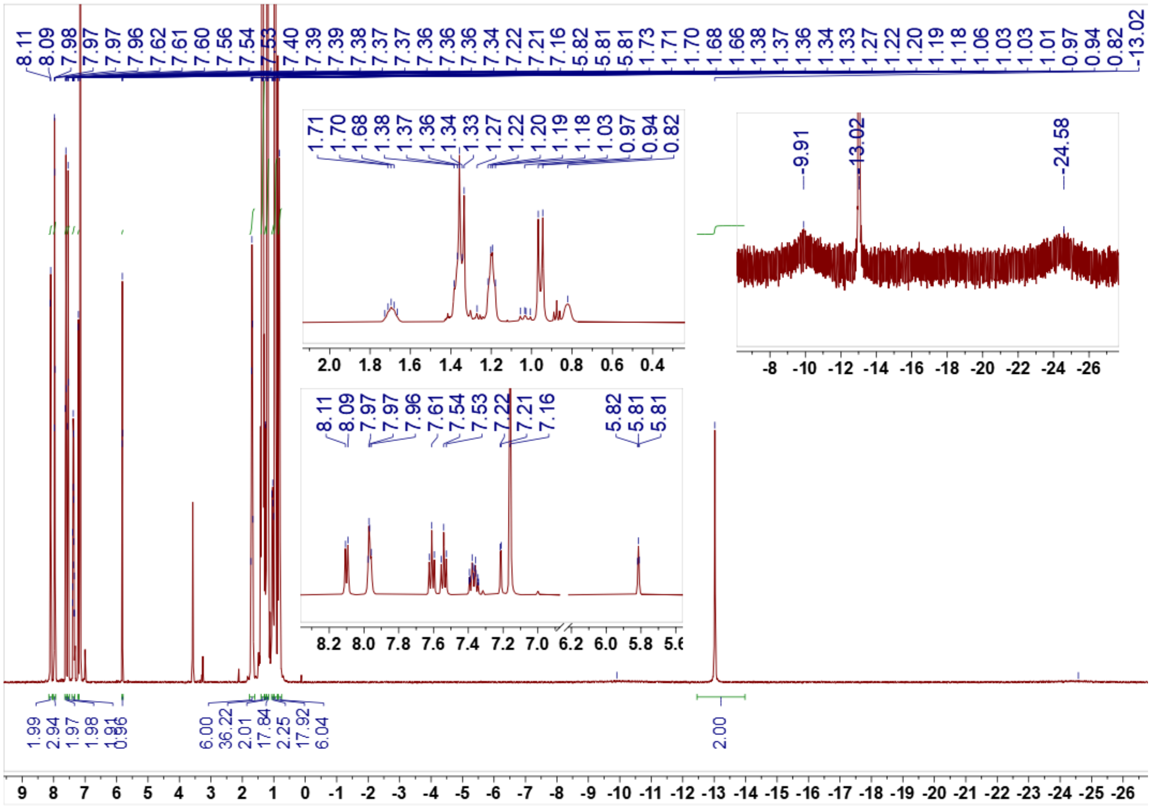

Figure S42. ^1H NMR spectrum (500 MHz, benzene-d_6) of **3**. Magnifications of several spectral regions are displayed as insets.

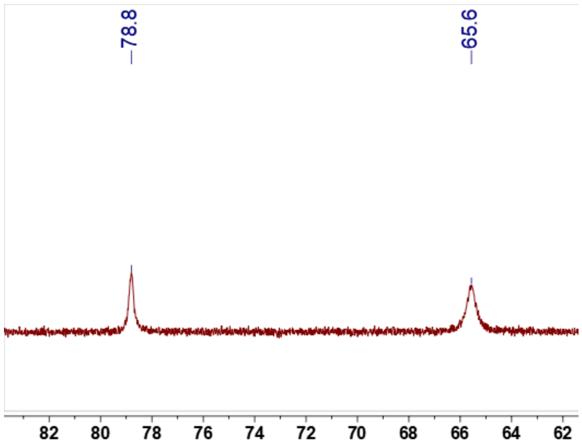

Figure S43. $^{31}P\{^1H\}$ NMR spectrum (203 MHz, benzene-d_6) of **3**.

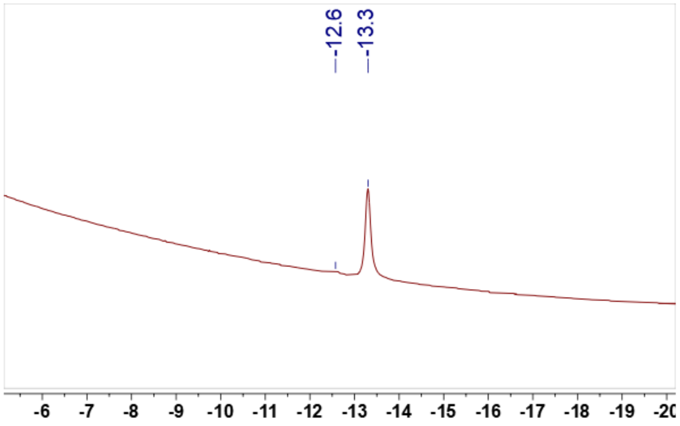

Figure S44. $^{11}B\{^1H\}$ NMR spectrum (193 MHz, benzene-d_6) of **3**.

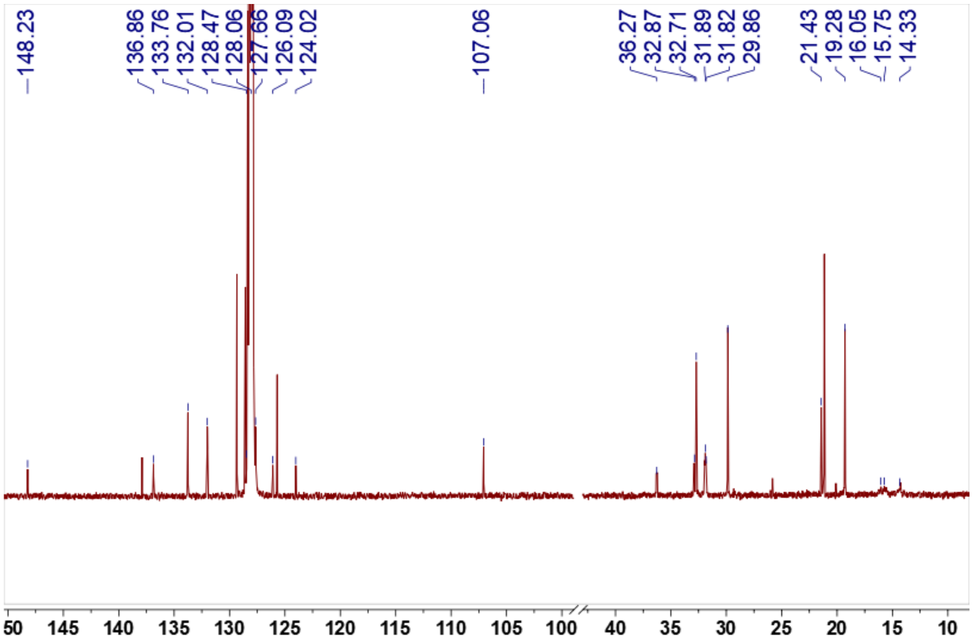

Figure S45. $^{13}C\{^1H\}$ NMR spectrum (151 MHz, benzene-d_6) of **3**. The chemical shifts of resonances due to adventitious toluene[4] are not labeled.

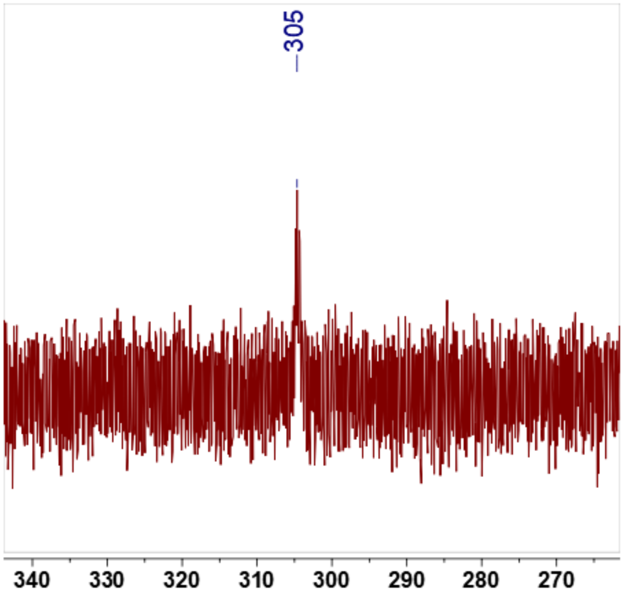

Figure S46. $^{29}Si\{^{1}H\}$ DEPT NMR spectrum (119 MHz, benzene-d_6) of **3**.

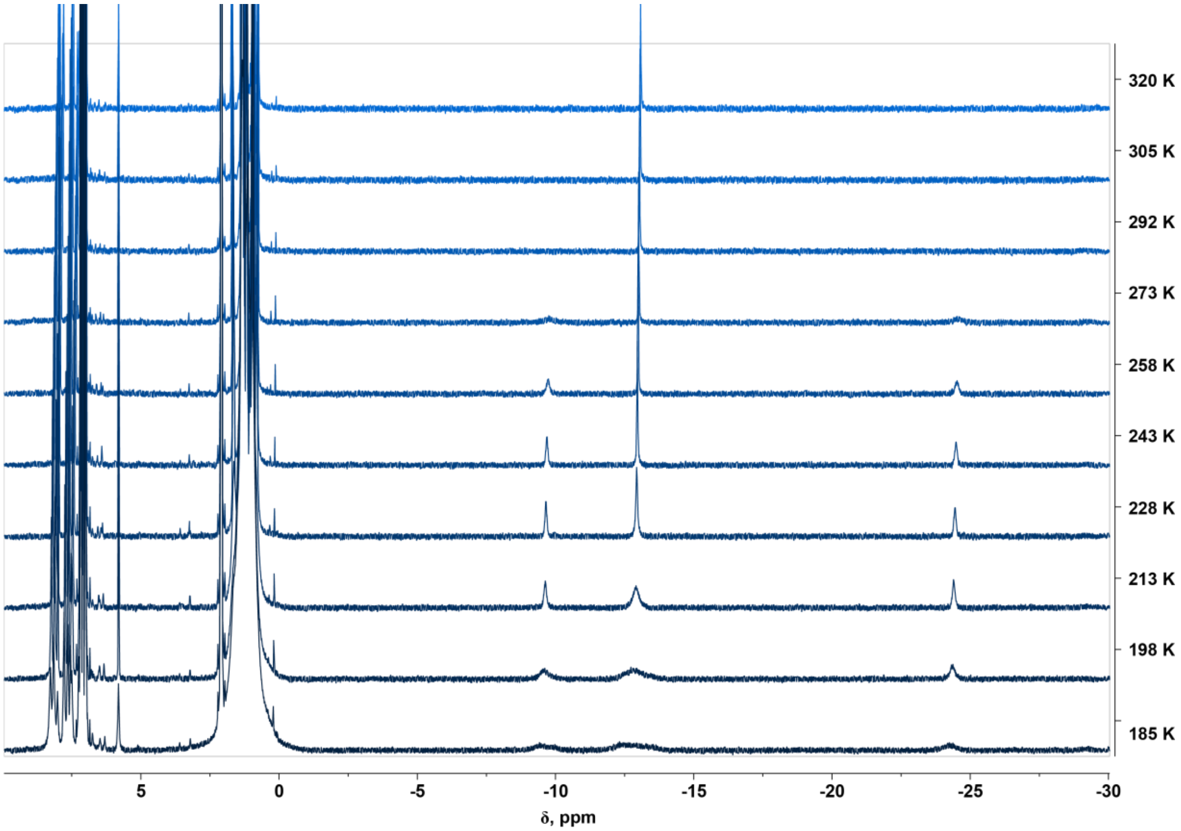

Figure S47. Variable-temperature ^{1}H NMR spectra (500 MHz, toluene-d_8) of **3**.

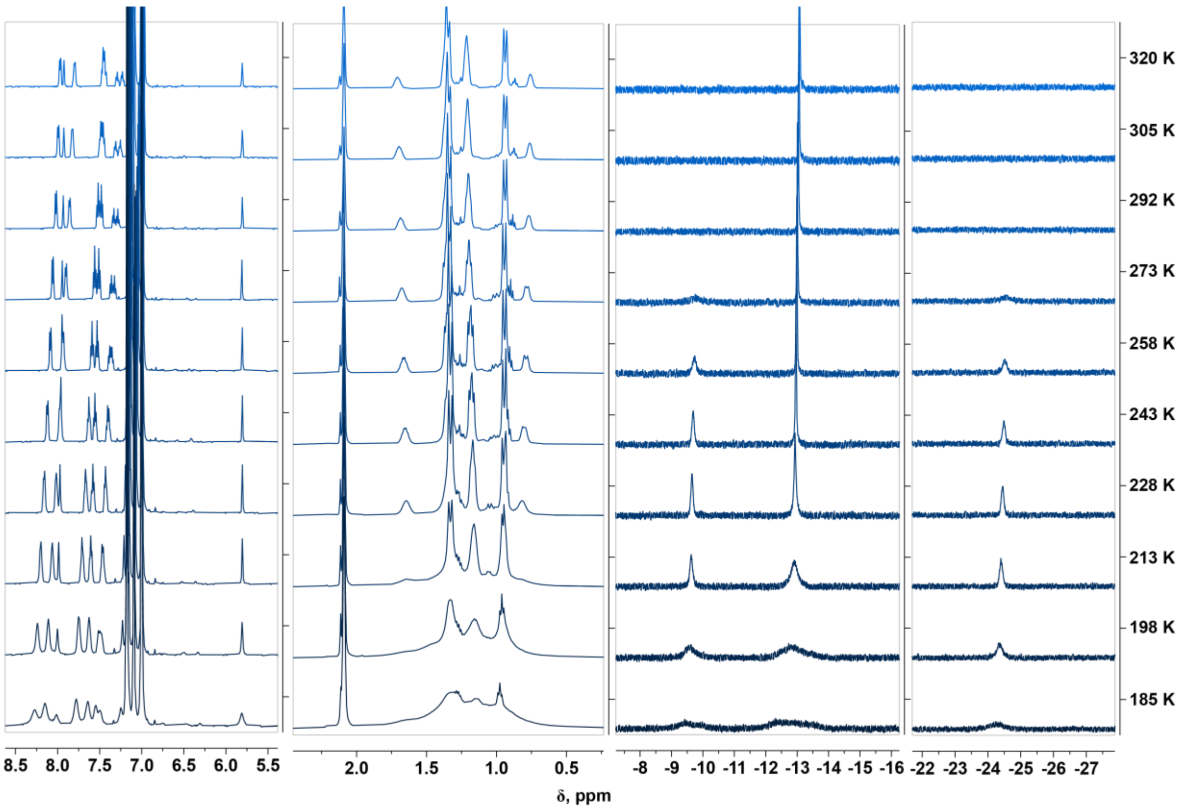

Figure S48. Variable-temperature ^{1}H NMR spectra (500 MHz, toluene-d_8) of **3**, showing magnifications of several spectral regions. Note that each region is independently scaled to provide greatest clarity of the spectroscopic features.

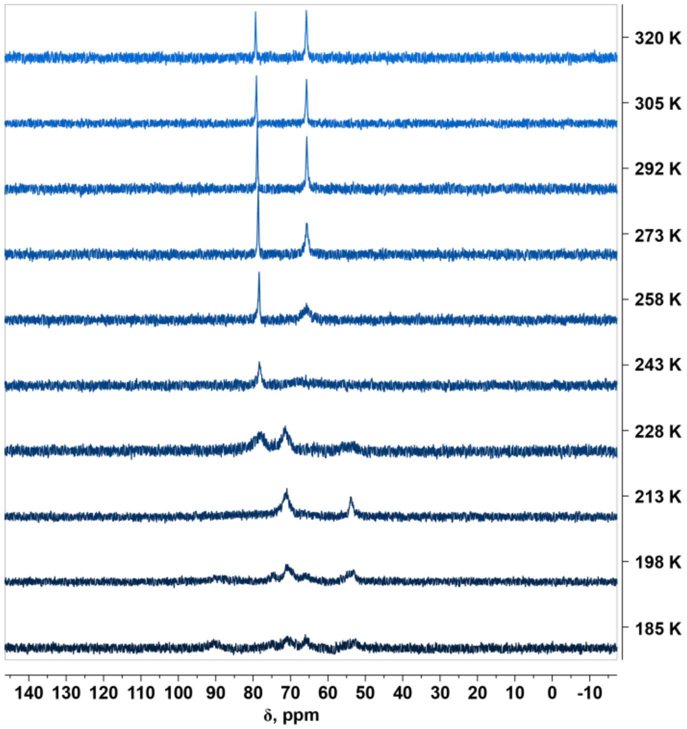

Figure S49. Variable-temperature ^{31}P{^{1}H} NMR spectra (202 MHz, toluene-d_8) of **3**.

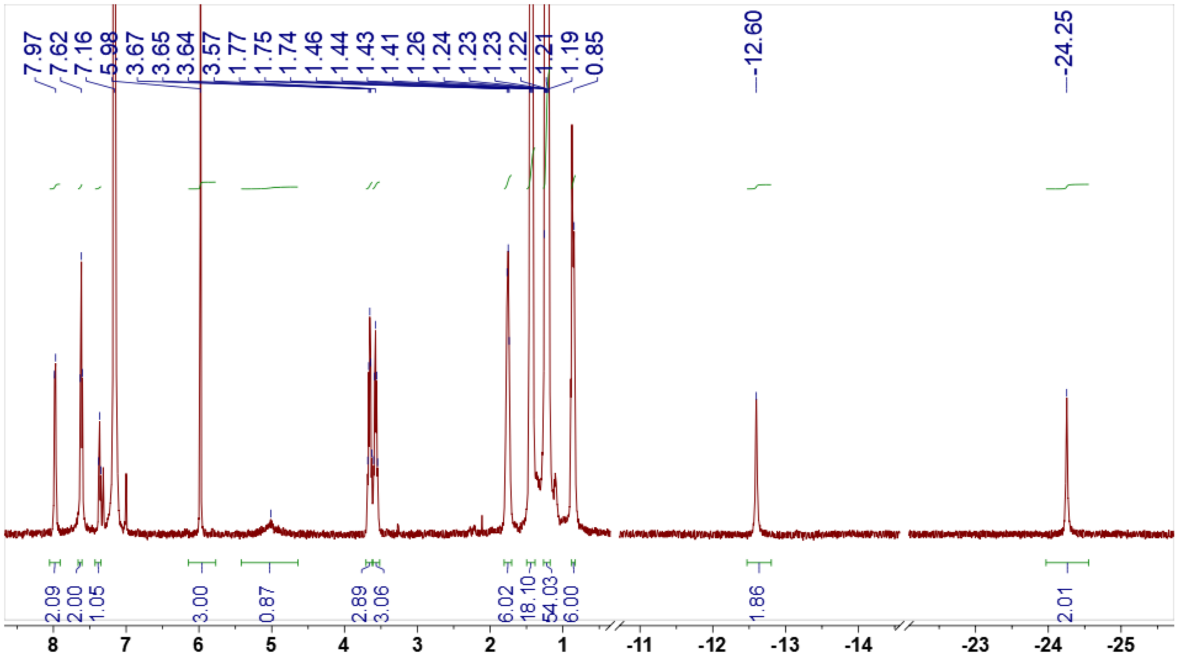

Figure S50. ${}^{1}H$ NMR spectrum (500 MHz, benzene-d_6) of **4**.

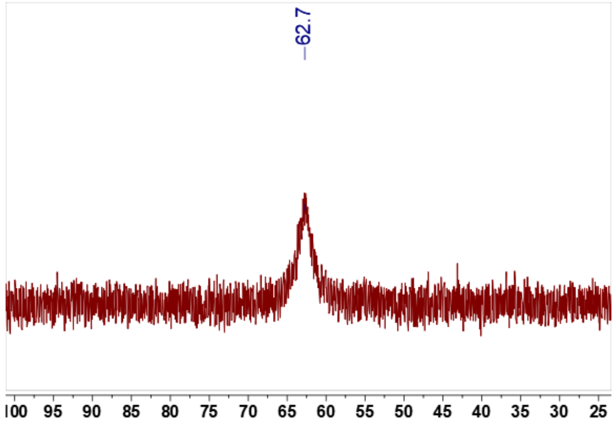

Figure S51. ${}^{31}P\{{}^{1}H\}$ NMR spectrum (203 MHz, benzene-d_6) of **4**.

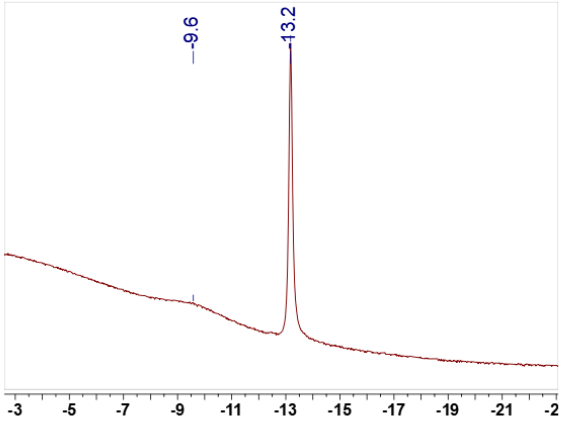

Figure S52. ${}^{11}B\{{}^{1}H\}$ NMR spectrum (193 MHz, benzene-d_6) of **4**.

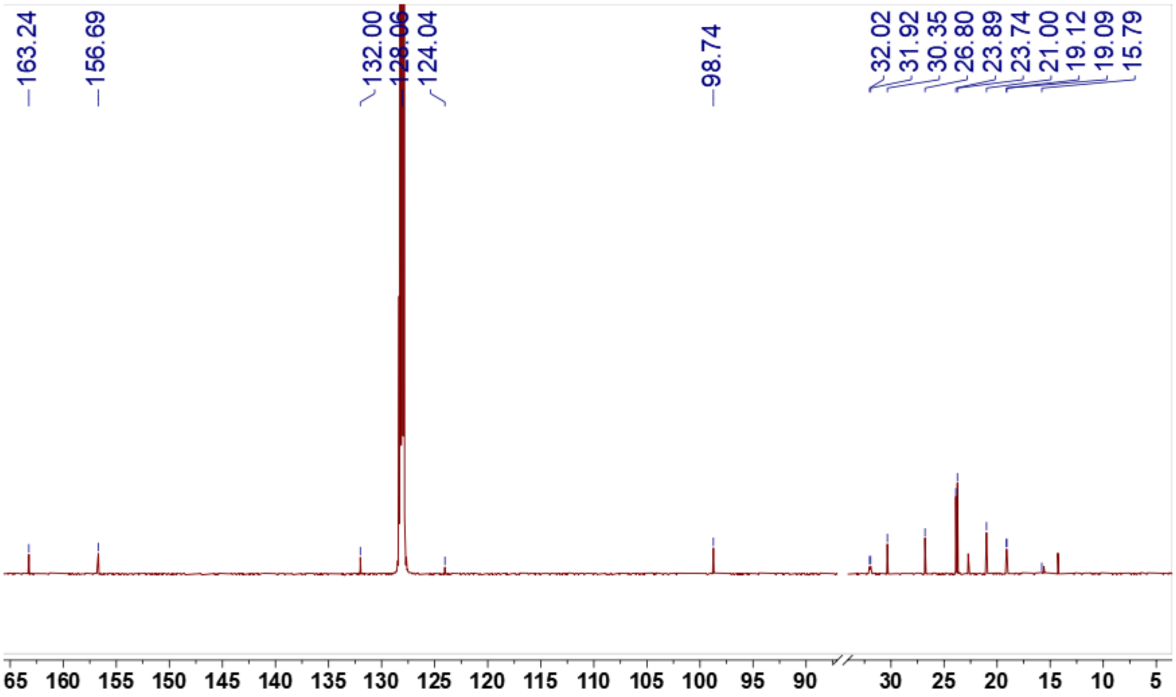

Figure S53. $^{13}C\{^{1}H\}$ NMR spectrum (151 MHz, benzene-d_6) of **4**.

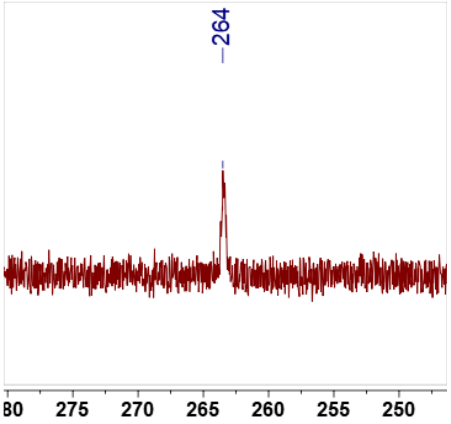

Figure S54. $^{29}Si\{^{1}H\}$ DEPT NMR spectrum (119 MHz, benzene-d_6) of **4**.

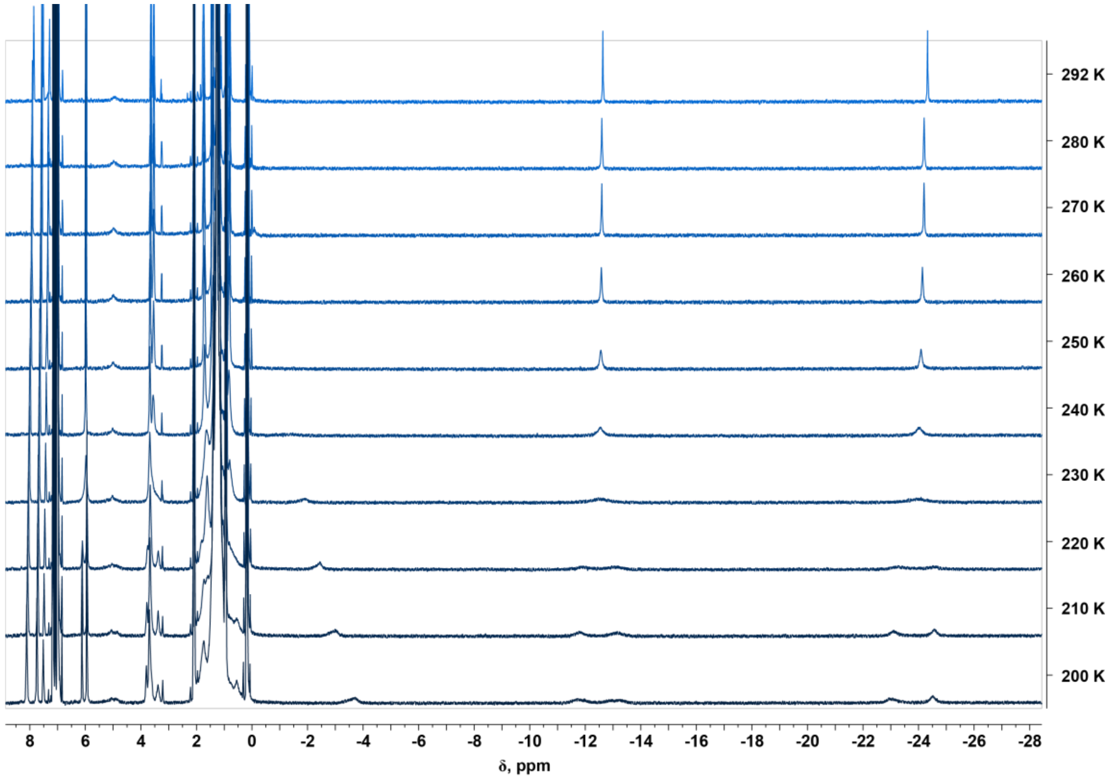

Figure S55. Variable-temperature ^{1}H NMR spectra (500 MHz, toluene-d_8) of **4**

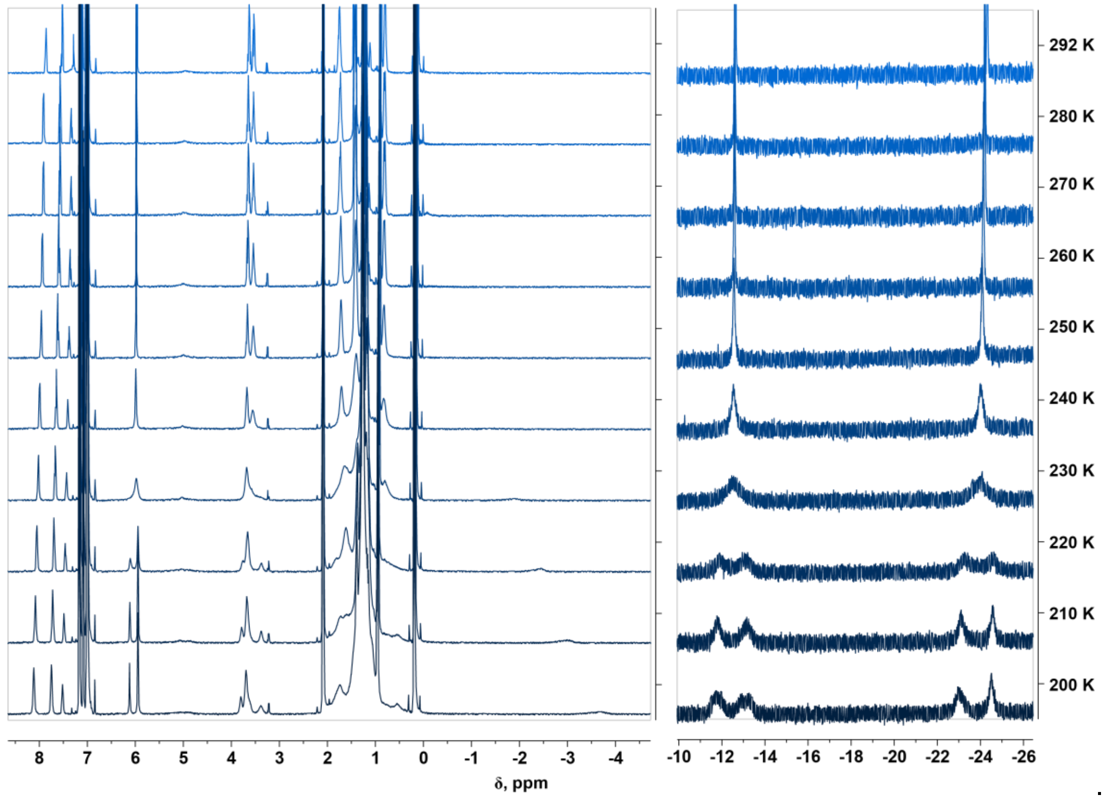

Figure S56. Variable-temperature ^{1}H NMR spectra (500 MHz, toluene-d_8) of **4**, showing magnifications of several spectral regions. Note that each region is independently scaled to provide greatest clarity of the spectroscopic features.

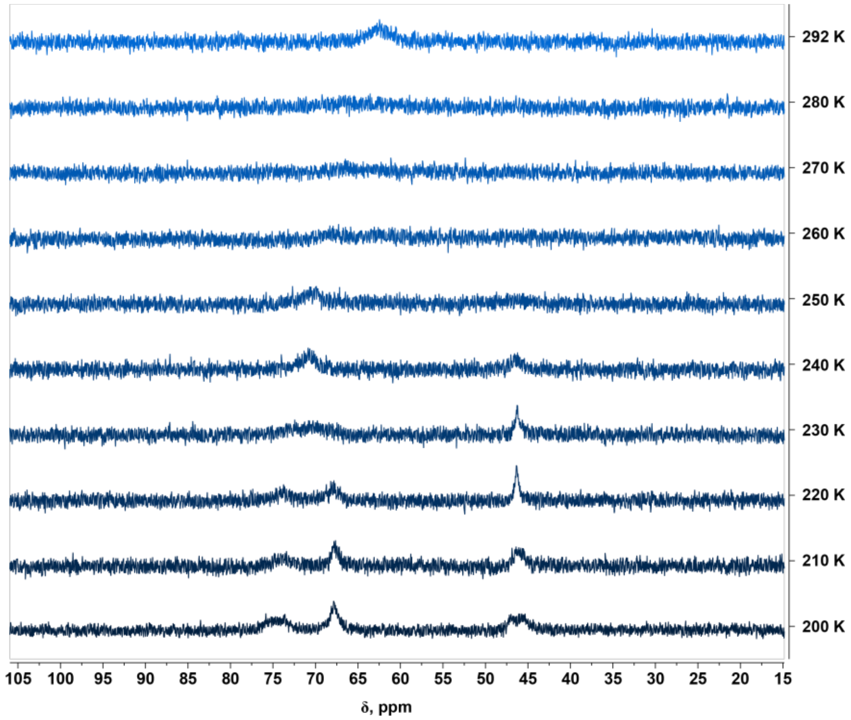

Figure S57. Variable-temperature $^{31}P\{^{1}H\}$ NMR spectra (202 MHz, toluene-d_8) of **4**.

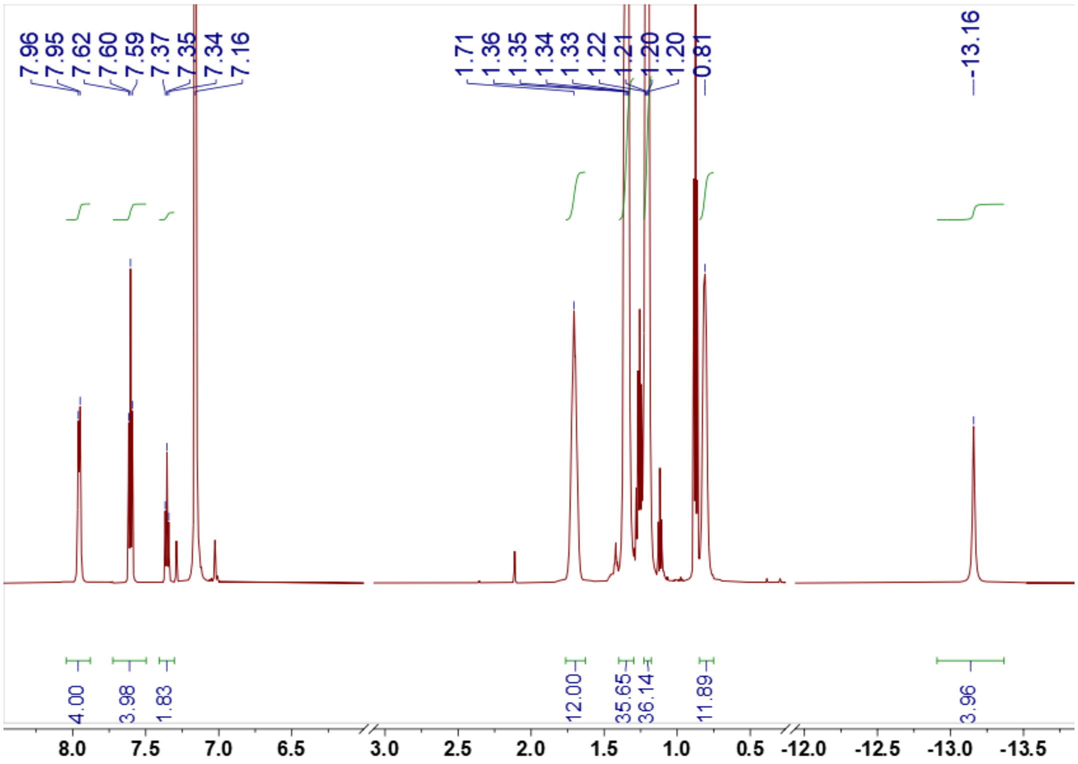

Figure S58. ^{1}H NMR spectrum (600 MHz, benzene-d_6) of **5**.

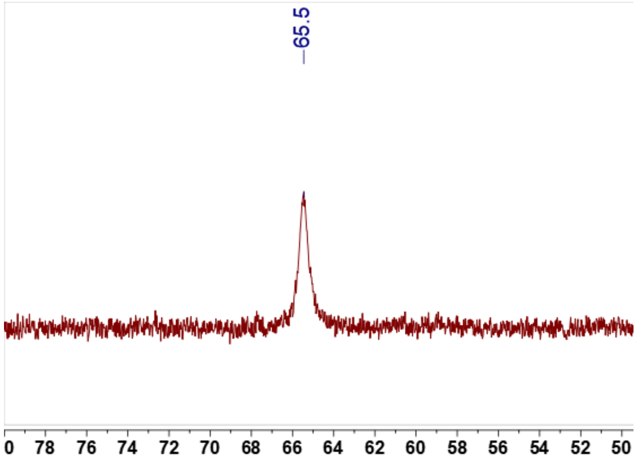

Figure S59. $^{31}P\{^{1}H\}$ NMR spectrum (243 MHz, benzene-d_6) of **5**.

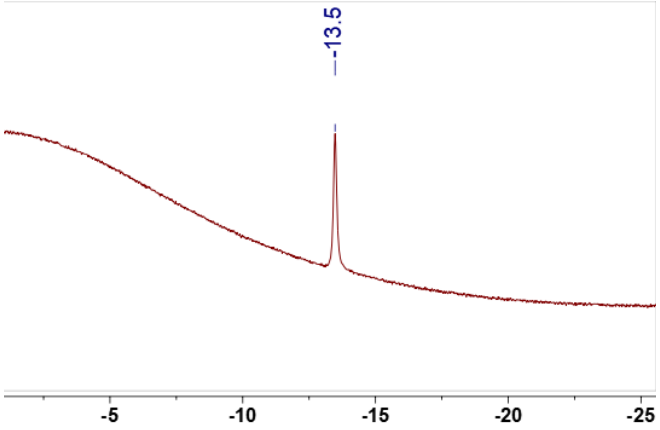

Figure S60. $^{11}B\{^{1}H\}$ NMR spectrum (193 MHz, benzene-d_6) of **5**.

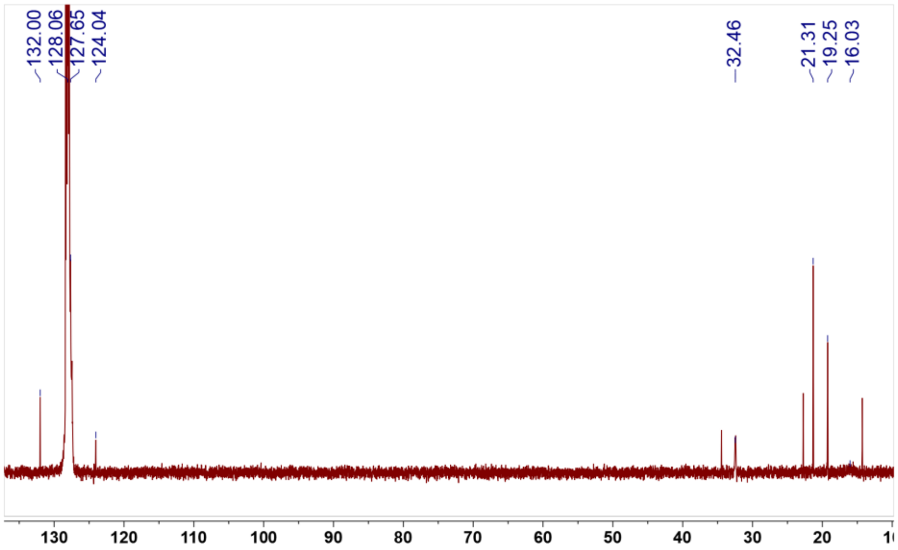

Figure S61. $^{13}C\{^{1}H\}$ NMR spectrum (151 MHz, benzene-d_6) of **5**.

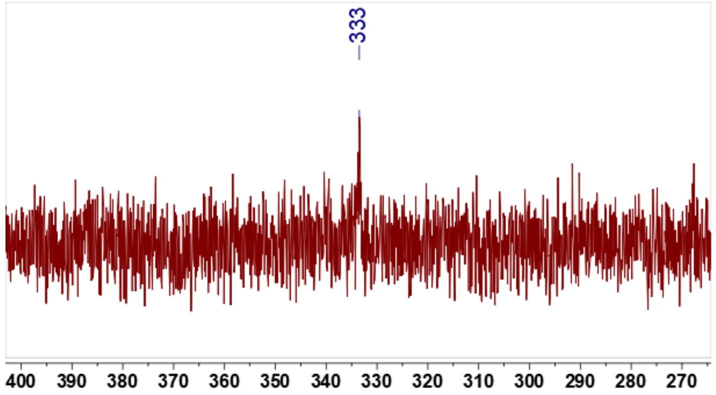

Figure S62. $^{29}Si\{^{1}H\}$ DEPT NMR spectrum (119 MHz, benzene-d_6) of **5**.

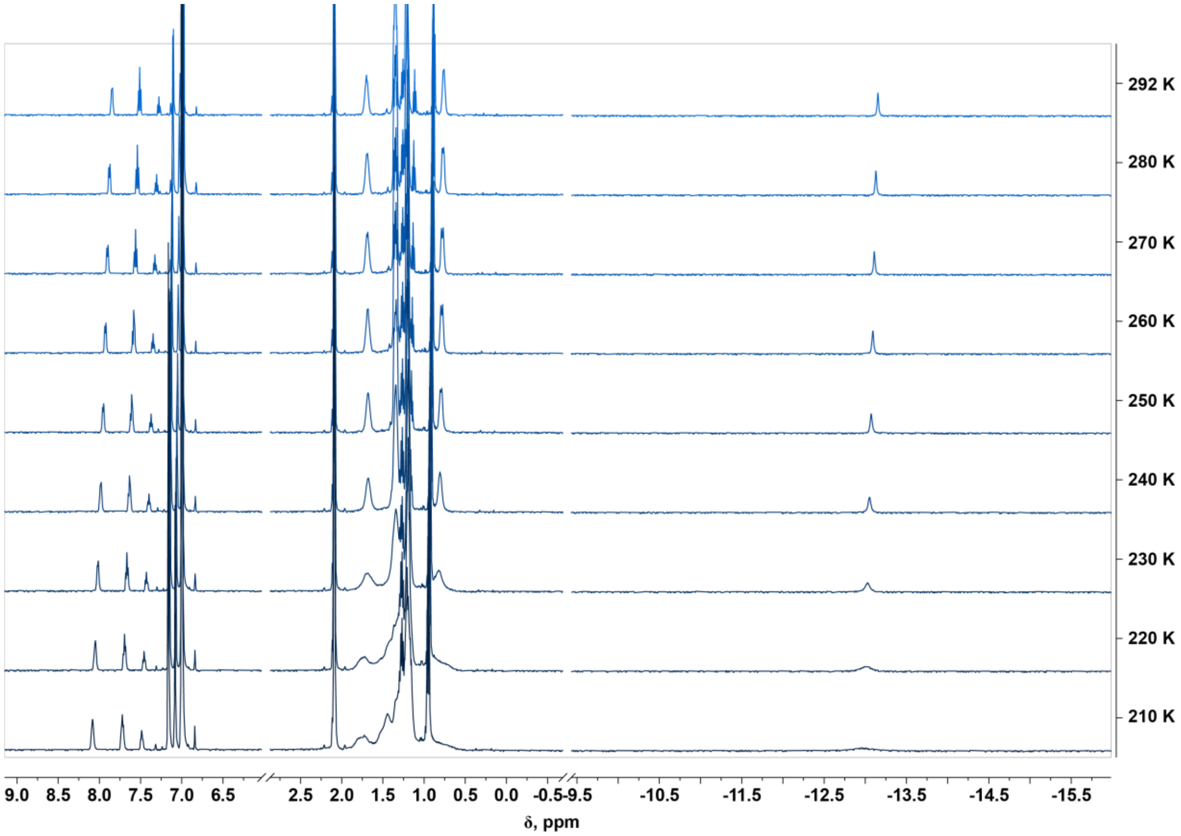

Figure S63. Variable-temperature ^{1}H NMR spectra (500 MHz, toluene-d_8) of **5**

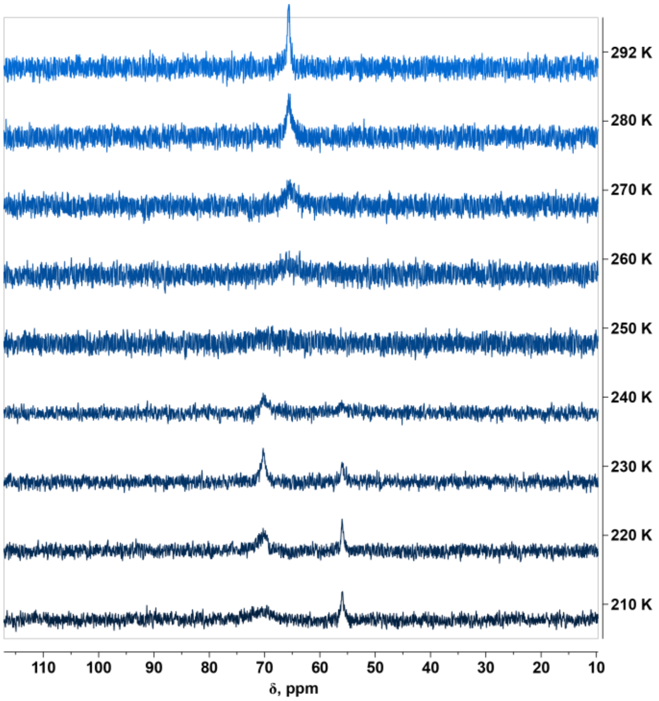

Figure S64. Variable-temperature $^{31}P\{^{1}H\}$ NMR spectra (202 MHz, toluene-d_8) of **5**.

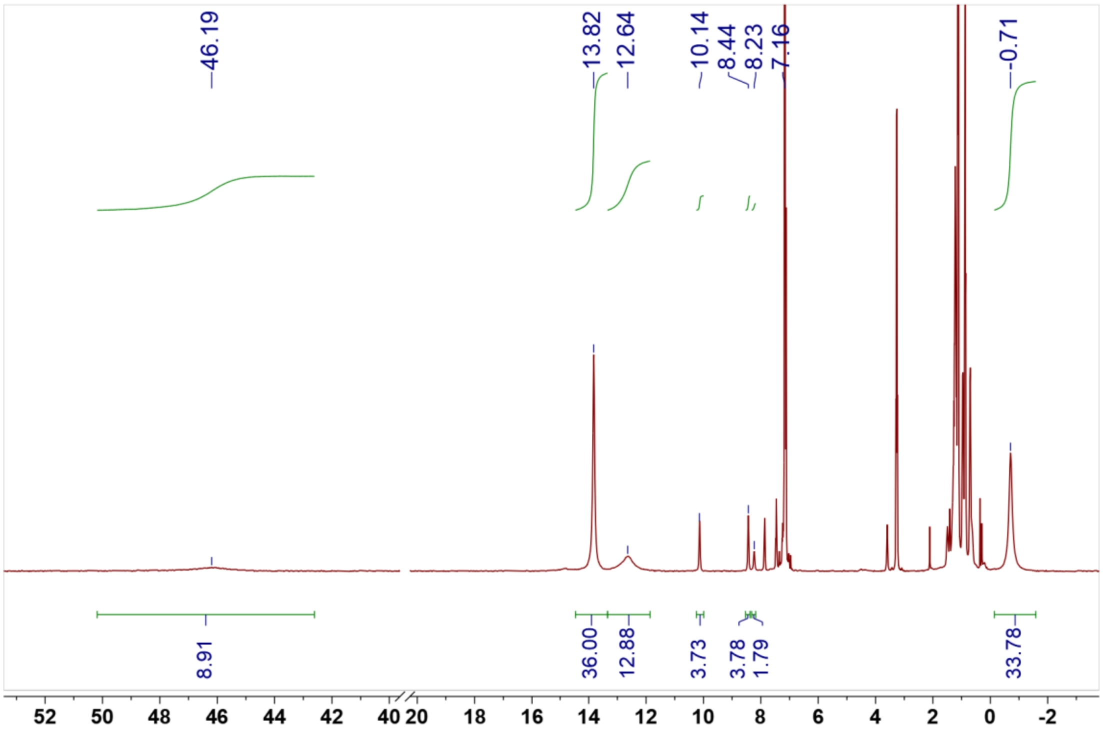

Figure S65. ^{1}H NMR spectrum (400 MHz, benzene-d_6) of **6**. The chemical shifts of resonances due to adventitious *n*-pentane[4] are not labeled. Two resonances in the region of 7.2–8.0 ppm have not been definitively assigned.

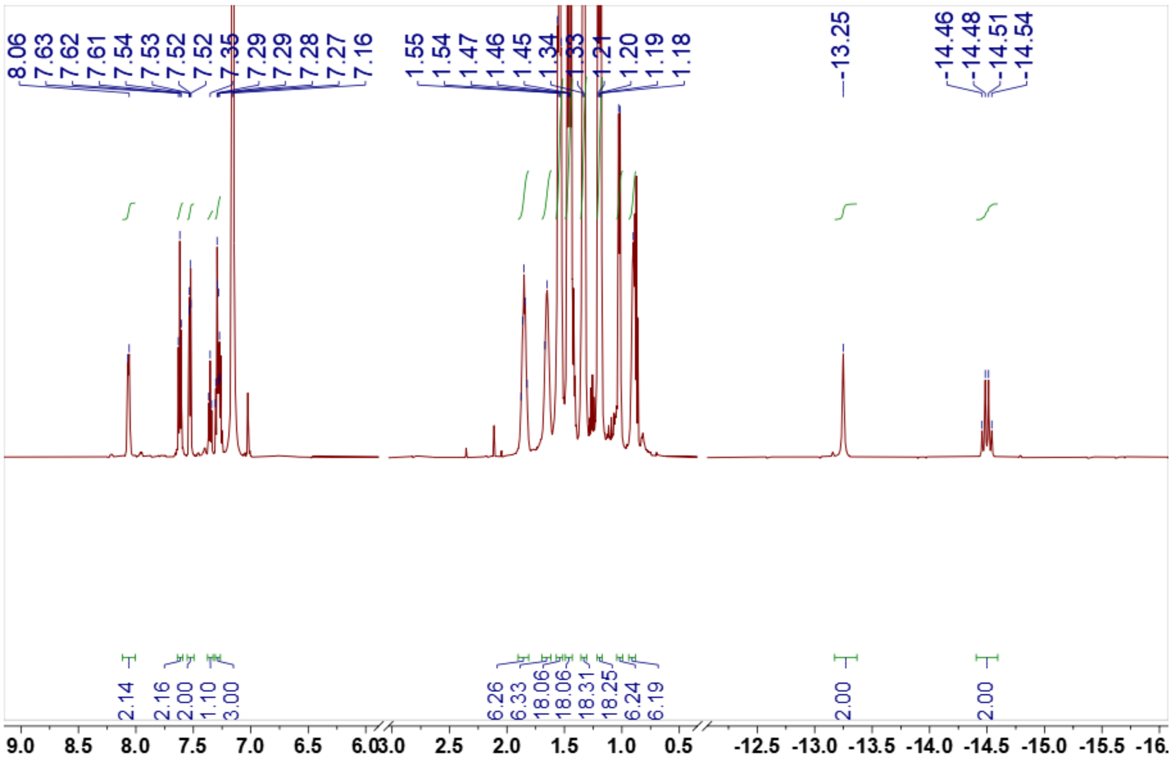

Figure S66. ^{1}H NMR spectrum (600 MHz, benzene-d_6) of **7**.

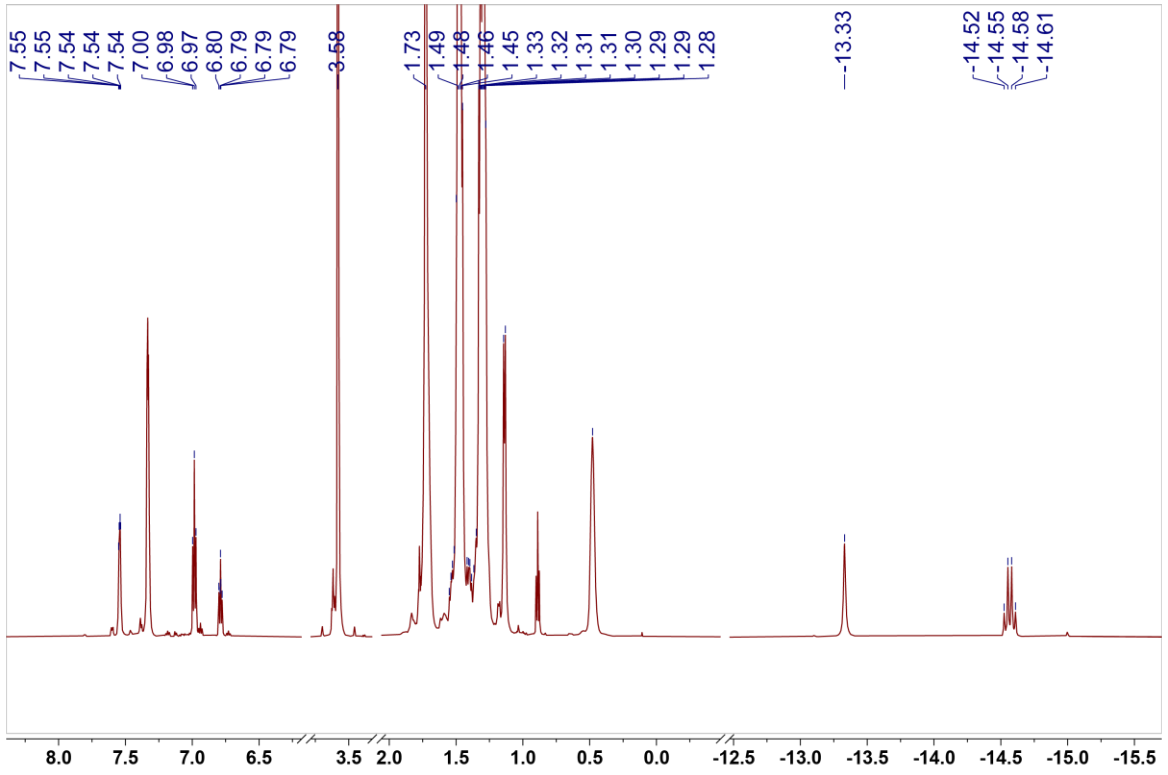

Figure S67. ^{1}H NMR spectrum (600 MHz, THF-d_8) of **7**.

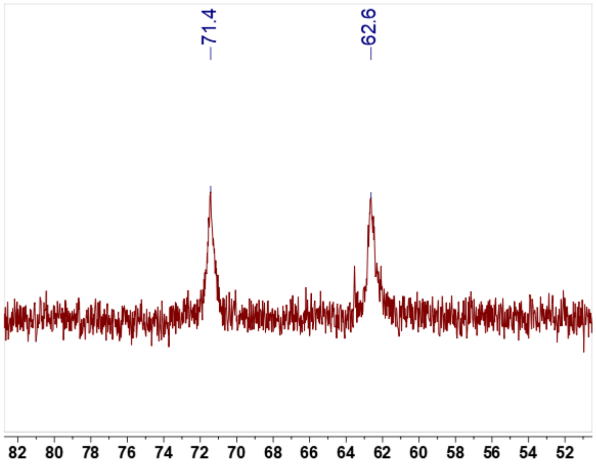

Figure S68. $^{31}P\{^{1}H\}$ NMR spectrum (203 MHz, benzene-d_6) of **7**. The sharp resonance at 63.5 ppm results from an unidentified impurity present in analytically pure samples of **7**.

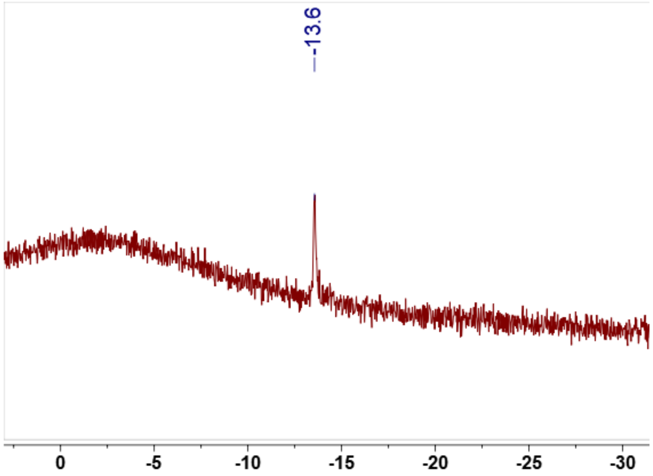

Figure S69. $^{11}B\{^{1}H\}$ NMR spectrum (193 MHz, benzene-d_6) of **7**.

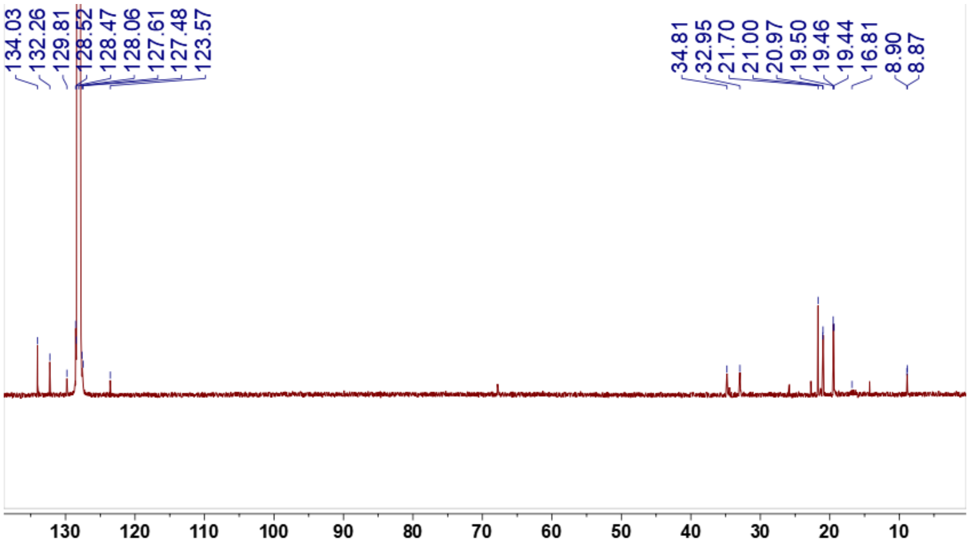

Figure S70. $^{13}C\{^{1}H\}$ NMR spectrum (151 MHz, benzene-d_6) of **7**.

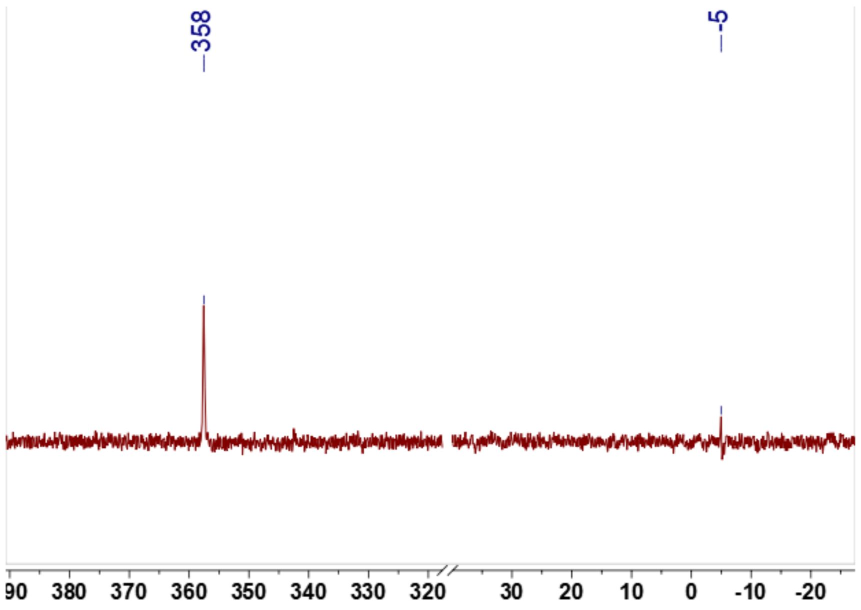

Figure S71. $^{29}Si\{^{1}H\}$ DEPT NMR spectrum (119 MHz, THF-d_8) of **7**.

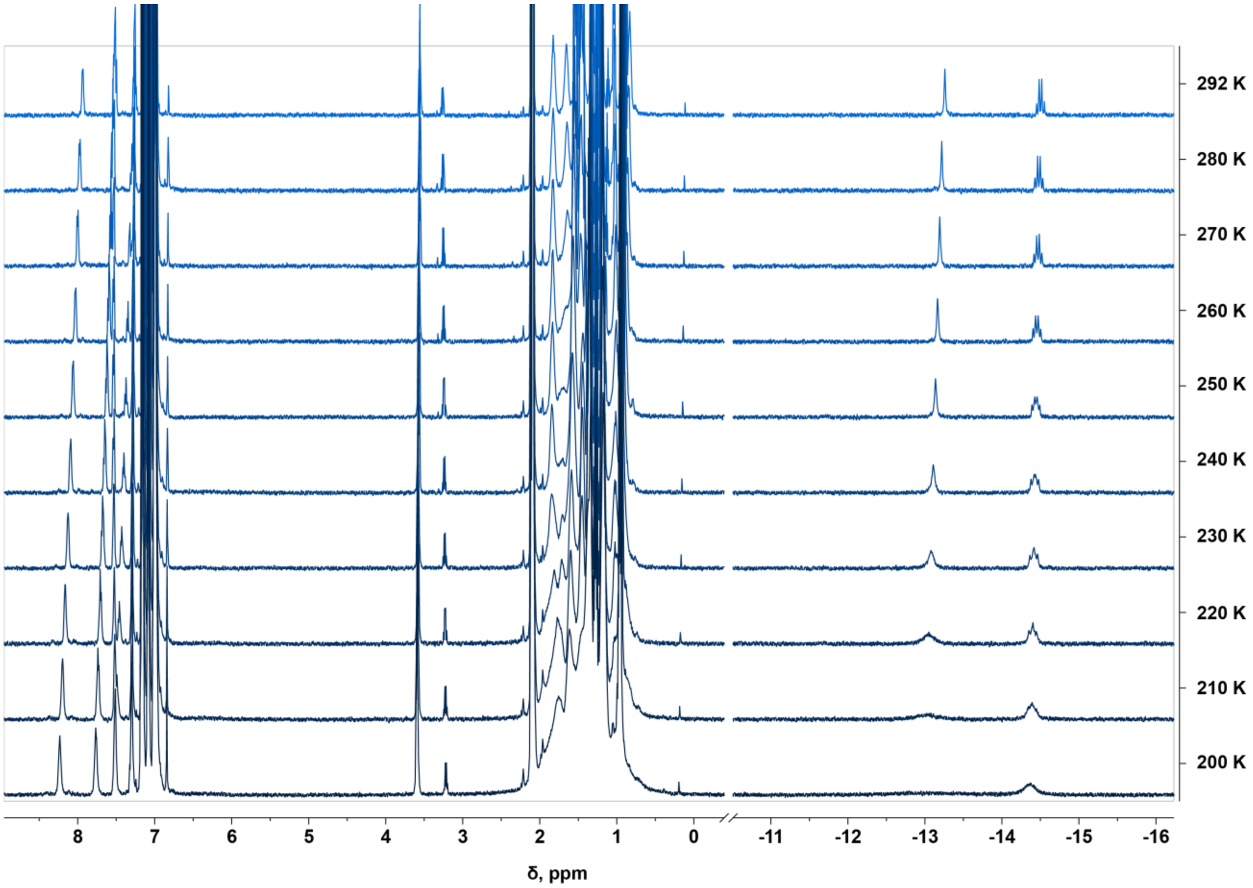

Figure S72. Variable-temperature ^{1}H NMR spectra (500 MHz, toluene-d_8) of **7**

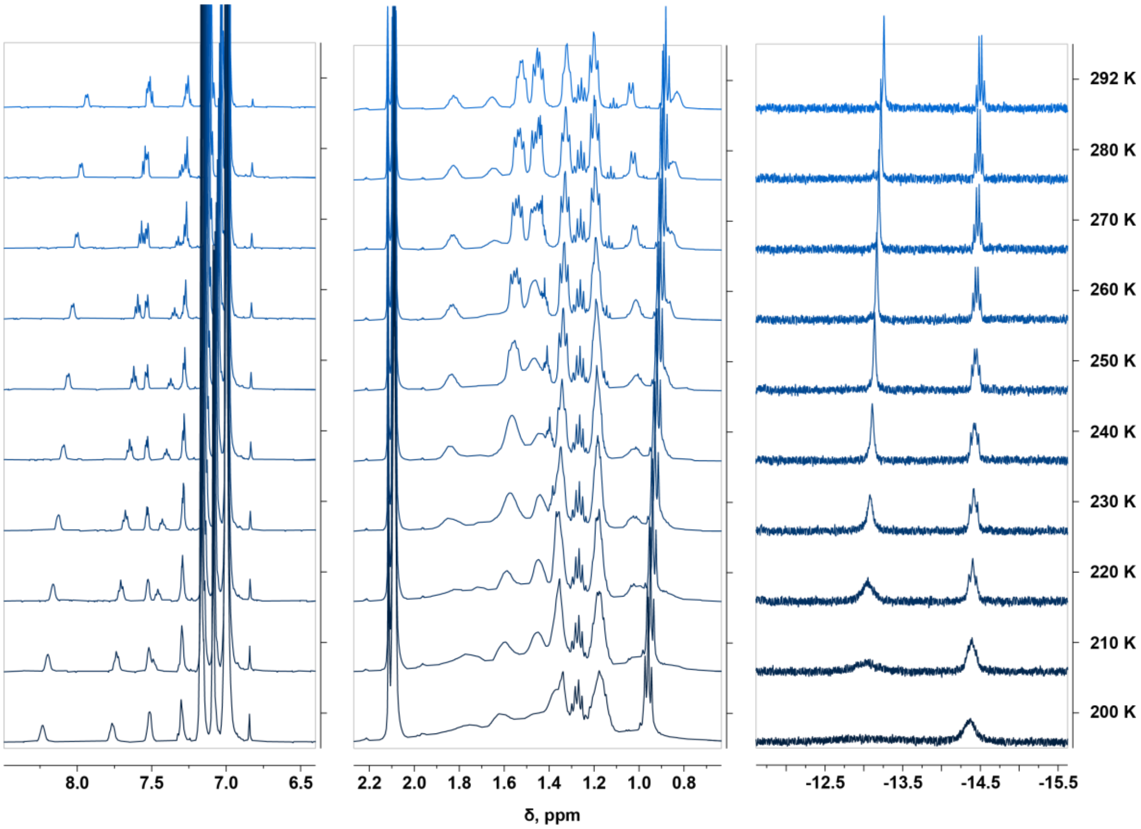

Figure S73. Variable-temperature ^{1}H NMR spectra (500 MHz, THF-d_8) of **7**, showing magnifications of several spectral regions. Note that each region is independently scaled to provide greatest clarity of the spectroscopic features.

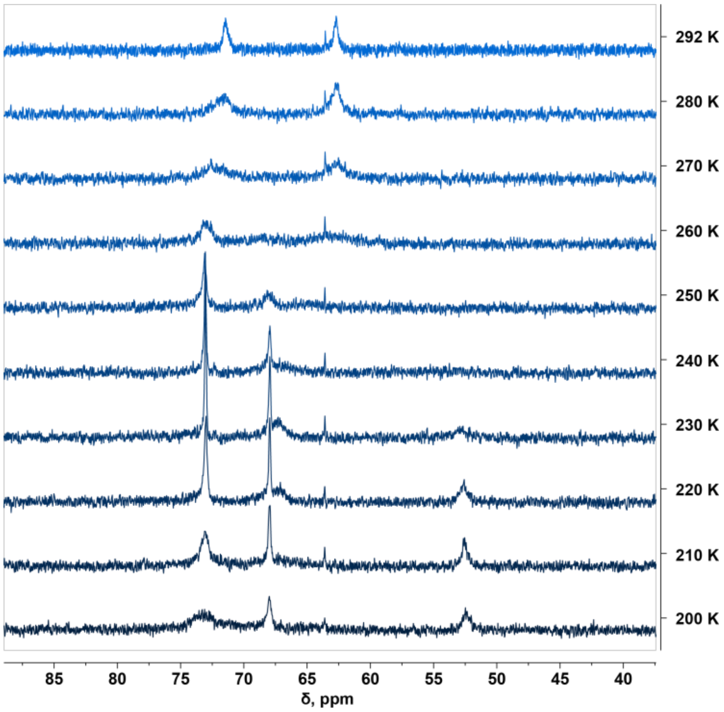

Figure S74. Variable-temperature ^{31}P{^{1}H} NMR spectra (202 MHz, THF-d_8) of **7.** The sharp resonance at 64.0 ppm results from an unidentified impurity present in analytically pure samples of **7**.

APPENDIX: COORDINATES OF COMPUTED STRUCTURES

73			
1*			
Co	-0.23427	0.303373	0.222067
P	1.111098	-0.00304	1.919499
P	1.178729	1.672684	-0.73313
P	0.561118	-1.43544	-0.84672
Si	-1.74285	1.647859	0.883629
H	-2.07692	1.940011	2.325651
H	-2	2.994985	0.25128
N	-7.12771	-0.82649	-0.49184
N	-3.50648	0.987818	0.49846
C	-5.95348	-0.23222	-0.16865
C	5.386491	-1.87612	-0.53554
H	4.738072	-2.73528	-0.73457
C	-5.36879	-0.37507	1.116052
H	-5.84028	-0.96679	1.898273
C	6.769658	-2.07001	-0.58396
H	7.172317	-3.0603	-0.81903
C	-5.23481	0.566118	-1.09579
H	-5.59857	0.738094	-2.1071
C	-4.16841	0.239302	1.393055
H	-3.69144	0.135573	2.37063
C	2.778897	0.908775	-1.13106
H	2.737494	0.554276	-2.17558
H	3.543699	1.702906	-1.12084
C	-4.03902	1.137365	-0.72389
H	-3.45761	1.740867	-1.42517
C	7.097832	0.244008	-0.03539
H	7.762425	1.089752	0.166842
C	4.809347	-0.62988	-0.24358
C	5.71596	0.417674	0.007451
H	5.327018	1.414095	0.249968
C	-7.82029	-1.64442	0.4846
H	-7.21192	-2.50857	0.799692
H	-8.74556	-2.02934	0.041697
H	-8.09265	-1.06477	1.381967
C	2.870659	-0.00032	1.466828
H	3.274808	0.999454	1.702517
H	3.398948	-0.69608	2.140919
C	0.970777	1.134738	3.343712
C	0.891813	-1.6002	2.779236
C	7.636055	-1.00853	-0.33515
H	8.719498	-1.15348	-0.37311
C	1.656347	3.172657	0.201094
C	0.575121	2.445534	-2.27428
C	-0.27266	-3.03098	-0.52796
C	0.403314	-1.38161	-2.66911
C	2.336423	-1.73324	-0.58477
H	2.437266	-2.51354	0.190362
H	2.74097	-2.17592	-1.51172
C	-7.67953	-0.67102	-1.82366
H	-7.90114	0.384498	-2.05309
H	-8.61749	-1.23313	-1.8921
H	-6.99559	-1.05897	-2.59667
B	3.190569	-0.37256	-0.13388
H	1.737239	0.913835	4.101668
H	-0.02652	1.044228	3.798204
H	1.092504	2.172382	3.004991
H	1.131315	-2.42246	2.093307
H	-0.1537	-1.70831	3.099592
H	1.557663	-1.67232	3.651999
H	2.305161	3.819604	-0.40822
H	2.209325	2.889682	1.106054
H	0.760345	3.735681	0.499408
H	1.345924	3.081831	-2.73426
H	-0.31376	3.053267	-2.04985
H	0.281427	1.663463	-2.98648
H	0.204176	-3.8551	-1.08036
H	-1.32737	-2.94727	-0.8276
H	-0.24753	-3.25628	0.546045
H	0.778961	-2.31192	-3.12141
H	0.982563	-0.54364	-3.07732
H	-0.65196	-1.23714	-2.93989
H	-1.2721	-0.51096	0.943313
H	-1.21795	0.513781	-0.89414

92			
1*_DMAP_adduct			
Co	-0.95419	-0.09709	0.075505
P	0.411199	-0.64806	1.692251
P	0.326347	1.584081	-0.50039
P	0.003211	-1.45793	-1.34635
Si	-2.66451	0.85443	0.943889
H	-3.05565	0.776269	2.393558
H	-3.17968	2.198813	0.518977
N	-7.35626	-2.50984	-1.02522
N	-4.27168	-0.07939	0.384473
C	-6.36306	-1.7094	-0.5625
C	4.853207	-1.50662	-1.04731
H	4.292653	-2.34735	-1.46846
C	-5.82818	-1.85632	0.742322

H	-6.19767	-2.61154	1.433378
C	6.249041	-1.5568	-1.09821
H	6.745721	-2.41948	-1.55338
C	-5.79476	-0.68001	-1.35565
H	-6.13803	-0.48157	-2.36939
C	-4.80396	-1.03485	1.158008
H	-4.36485	-1.13634	2.153351
C	1.988331	1.080741	-1.03747
H	1.98021	0.974987	-2.13617
H	2.676155	1.917056	-0.82865
C	-4.76846	0.08678	-0.84939
H	-4.29665	0.867911	-1.4506
C	6.351144	0.572336	0.004665
H	6.929545	1.400582	0.426251
C	4.157207	-0.42762	-0.47941
C	4.958731	0.604716	0.044
H	4.47359	1.4735	0.505414
C	-7.89392	-3.55839	-0.18158
H	-7.12392	-4.29945	0.091399
H	-8.68986	-4.08411	-0.72093
H	-8.32869	-3.15188	0.746503
C	2.167667	-0.39958	1.284159
H	2.491728	0.541299	1.761944
H	2.749058	-1.1949	1.781558
C	0.174371	0.136978	3.325275
C	0.331084	-2.40586	2.189319
C	7.008778	-0.51512	-0.57225
H	8.101354	-0.54888	-0.60953
C	0.671749	2.846785	0.771874
C	-0.34837	2.656985	-1.81635
C	-0.66961	-3.15626	-1.42689
C	-0.16665	-0.99318	-3.10834
C	1.800031	-1.64475	-1.12608
H	1.972209	-2.57111	-0.54925
H	2.243898	-1.82139	-2.12151
C	-7.86354	-2.33585	-2.37165
H	-8.30256	-1.33474	-2.51647
H	-8.64905	-3.07646	-2.55976
H	-7.07503	-2.48257	-3.12867
B	2.519869	-0.35328	-0.35328
H	0.930989	-0.20569	4.047557
H	-0.83035	-0.10201	3.703526
H	0.241987	1.227645	3.222353
H	0.613187	-3.0468	1.344179
H	-0.69677	-2.65751	2.486067
H	1.019193	-2.61014	3.02303

H	1.261062	3.678163	0.355219
H	1.236325	2.405889	1.603127
H	-0.27579	3.234863	1.166173
H	0.368087	3.44185	-2.10214
H	-1.27711	3.121382	-1.45362
H	-0.59417	2.051244	-2.69856
H	-0.1143	-3.77798	-2.14583
H	-1.72815	-3.10761	-1.72056
H	-0.61934	-3.62229	-0.43415
H	0.307497	-1.74062	-3.76241
H	0.310687	-0.02204	-3.29073
H	-1.23309	-0.90519	-3.35884
H	-1.88194	-1.17159	0.550971
H	-1.94964	0.266603	-0.98811
N	-2.51171	3.683355	2.948197
C	-1.62192	3.286082	3.85573
H	-1.87532	2.379398	4.417567
C	-2.20687	4.794462	2.277818
H	-2.93955	5.12522	1.531877
C	-1.03896	5.526399	2.448408
H	-0.87548	6.411998	1.835213
C	-0.07413	5.081579	3.379809
C	-0.41545	3.924296	4.113135
H	0.25373	3.505209	4.863961
N	1.127152	5.717418	3.546997
C	1.472002	6.837091	2.701139
H	1.535381	6.556059	1.633727
H	2.448411	7.232507	3.005688
H	0.739767	7.656006	2.794655
C	2.132221	5.130173	4.404168
H	2.424942	4.11721	4.072567
H	3.030454	5.759139	4.395035
H	1.787517	5.060073	5.449459
**92**			
**TS**			
Co	-0.31042	-0.43608	-0.22929
P	1.083985	-1.20449	1.274789
P	1.16787	1.080649	-0.84246
P	0.348215	-1.8674	-1.74772
Si	-2.07796	0.52021	0.63406
H	-2.6052	0.304643	2.027877
H	-2.81829	1.684638	0.04133
N	-6.33744	-3.31777	-1.69634
N	-3.59232	-0.63931	-0.04582
C	-5.45132	-2.44488	-1.15804
C	5.147236	-2.63101	-1.80704

H	4.444364	-3.3907	-2.16469
C	-4.90094	-2.63396	0.136414
H	-5.17203	-3.48824	0.754614
C	6.515072	-2.8667	-1.97966
H	6.851813	-3.78932	-2.46335
C	-5.01729	-1.28773	-1.85503
H	-5.38295	-1.04923	-2.85246
C	-3.99309	-1.72213	0.631591
H	-3.54609	-1.85886	1.620621
C	2.666936	0.332159	-1.55985
H	2.513693	0.24506	-2.64958
H	3.489458	1.056819	-1.43709
C	-4.10568	-0.43731	-1.26484
H	-3.75143	0.454853	-1.78965
C	6.993964	-0.76377	-0.92271
H	7.712116	-0.01794	-0.56699
C	4.650847	-1.46597	-1.19755
C	5.626057	-0.54711	-0.76045
H	5.30285	0.379414	-0.27021
C	-6.74084	-4.49543	-0.95271
H	-5.88211	-5.14952	-0.72655
H	-7.45574	-5.07075	-1.55146
H	-7.2316	-4.22852	-0.00209
C	2.823111	-1.22101	0.719459
H	3.329734	-0.36108	1.190932
H	3.3033	-2.11638	1.152175
C	1.136344	-0.45782	2.943232
C	0.779466	-2.94512	1.747895
C	7.44968	-1.93154	-1.53918
H	8.520844	-2.10876	-1.67267
C	1.899485	2.208511	0.401831
C	0.591285	2.302189	-2.07369
C	-0.58198	-3.44127	-1.81666
C	0.127686	-1.3311	-3.48385
C	2.104788	-2.338	-1.66583
H	2.168318	-3.28805	-1.10513
H	2.440081	-2.56762	-2.69288
C	-6.862	-3.09092	-3.02894
H	-7.40869	-2.1356	-3.09404
H	-7.5599	-3.89671	-3.28234
H	-6.0612	-3.08285	-3.78728
B	3.050923	-1.1752	-0.93775
H	1.900925	-0.94355	3.568623
H	0.152152	-0.56902	3.421968
H	1.363816	0.613556	2.86754
H	0.87359	-3.59513	0.868399

H	-0.24153	-3.04493	2.142928
H	1.502812	-3.2785	2.50719
H	2.567873	2.931306	-0.09022
H	2.491943	1.6327	1.124104
H	1.117972	2.750333	0.945953
H	1.408314	2.963847	-2.3991
H	-0.2156	2.90769	-1.63614
H	0.18329	1.775575	-2.94705
H	-0.19677	-4.10405	-2.60693
H	-1.64343	-3.22199	-2.00663
H	-0.51288	-3.95677	-0.84953
H	0.465588	-2.11093	-4.18356
H	0.700601	-0.41559	-3.6782
H	-0.93567	-1.11486	-3.66046
H	-1.25957	-1.45536	0.276127
H	-1.2896	0.036764	-1.25503
N	-1.29655	2.568303	1.840004
C	-0.746	2.553841	3.054984
H	-0.81481	1.609228	3.603816
C	-1.25201	3.722189	1.171898
H	-1.72837	3.721793	0.184373
C	-0.64399	4.873593	1.644741
H	-0.65036	5.767022	1.021387
C	-0.02002	4.858281	2.916517
C	-0.10493	3.638127	3.631409
H	0.325387	3.527352	4.626154
N	0.619836	5.948689	3.421708
C	0.704076	7.164728	2.640196
H	1.22763	7.002167	1.682344
H	1.263112	7.918872	3.206018
H	-0.29368	7.579687	2.418278
C	1.262838	5.874595	4.717061
H	2.056597	5.108114	4.739265
H	1.722211	6.842741	4.947329
H	0.542169	5.643579	5.519618
**92**			
**Int**			
Co	-0.84578	0.427323	0.450029
P	0.574598	-0.36935	1.912972
P	0.642552	1.891285	-0.236
P	-0.27883	-1.03911	-1.06349
Si	-2.57308	1.452244	1.328196
H	-3.16865	1.167163	2.681436
H	-3.39674	2.51002	0.646822
N	-6.67516	-2.6261	-1.18499
N	-4.13139	0.179159	0.588975

C	-5.85467	-1.70692	-0.60202
C	4.502761	-1.96747	-1.18051
H	3.772844	-2.72803	-1.47623
C	-5.34077	-1.8815	0.706129
H	-5.58655	-2.75589	1.306738
C	5.860076	-2.25632	-1.3449
H	6.165676	-3.22052	-1.76345
C	-5.4642	-0.52153	-1.27
H	-5.80638	-0.29104	-2.27757
C	-4.49617	-0.92632	1.23922
H	-4.06979	-1.05011	2.239775
C	2.100573	1.101766	-0.98798
H	1.915829	1.004213	-2.07174
H	2.944965	1.805451	-0.89986
C	-4.61584	0.368603	-0.63803
H	-4.28729	1.284385	-1.13999
C	6.412694	-0.10174	-0.44222
H	7.156217	0.64439	-0.14442
C	4.049788	-0.74859	-0.65033
C	5.054483	0.168339	-0.28771
H	4.762914	1.136164	0.137941
C	-7.01554	-3.8433	-0.48054
H	-6.11987	-4.43583	-0.22749
H	-7.65608	-4.46346	-1.11826
H	-7.56811	-3.64011	0.452767
C	2.28929	-0.43989	1.299401
H	2.837663	0.406738	1.747536
H	2.762549	-1.34499	1.718001
C	0.737696	0.358486	3.58739
C	0.227598	-2.09907	2.400716
C	6.826045	-1.32329	-0.97561
H	7.890028	-1.5433	-1.10165
C	1.434576	3.021106	0.972206
C	0.060941	3.115918	-1.46512
C	-1.25846	-2.58558	-1.06703
C	-0.56594	-0.52845	-2.79863
C	1.467155	-1.55336	-1.05041
H	1.532108	-2.50068	-0.48572
H	1.762717	-1.79493	-2.08658
C	-7.17449	-2.40342	-2.52489
H	-7.77298	-1.47893	-2.59005
H	-7.82104	-3.24021	-2.81359
H	-6.35859	-2.33633	-3.26502
B	2.467448	-0.40963	-0.36378
H	1.530024	-0.15101	4.156739
H	-0.21537	0.258739	4.127992

H	0.987594	1.425314	3.510497
H	0.279405	-2.75168	1.520124
H	-0.78674	-2.16492	2.818719
H	0.958938	-2.45715	3.140639
H	2.097813	3.729669	0.452851
H	2.043799	2.437681	1.674497
H	0.685442	3.581494	1.54348
H	0.88968	3.735289	-1.84044
H	-0.69968	3.764639	-1.00791
H	-0.41069	2.592742	-2.30759
H	-0.92436	-3.2781	-1.85507
H	-2.31861	-2.33416	-1.22123
H	-1.17298	-3.08231	-0.09189
H	-0.29313	-1.3327	-3.49896
H	0.031669	0.359377	-3.04144
H	-1.62791	-0.27445	-2.9255
H	-1.80095	-0.56534	0.983119
H	-1.82721	0.902944	-0.56374
N	-1.82673	3.263291	2.344463
C	-1.26794	3.213608	3.554572
H	-1.32497	2.248713	4.065147
C	-1.80974	4.434419	1.706055
H	-2.30542	4.446458	0.72981
C	-1.2116	5.575258	2.209288
H	-1.23808	6.486453	1.61373
C	-0.575	5.528971	3.472814
C	-0.63964	4.289199	4.154105
H	-0.19936	4.154059	5.140894
N	0.060233	6.611909	4.001938
C	0.129721	7.846377	3.250246
H	0.636993	7.708828	2.279844
H	0.699104	8.587384	3.82311
H	-0.87156	8.267894	3.057962
C	0.717427	6.508276	5.286982
H	1.520822	5.751657	5.278987
H	1.168473	7.474273	5.541535
H	0.007753	6.248196	6.090407
**92**			
**TS'**			
Co	-0.29961	-0.03325	-0.46982
P	0.800554	-1.24152	0.9866
P	1.430204	1.334796	-0.68303
P	0.329104	-1.2402	-2.17692
Si	-1.93902	0.824579	0.730403
H	-2.31031	0.381946	2.121575
H	-3.01104	1.719695	0.186218

N	-6.044	-3.80628	-1.14053
N	-3.619	-0.68882	0.248454
C	-5.26332	-2.78932	-0.6839
C	4.914486	-2.80207	-1.90883
H	4.142067	-3.37486	-2.43222
C	-4.6162	-2.84461	0.575804
H	-4.72324	-3.70432	1.236093
C	6.238984	-3.23932	-2.00673
H	6.476185	-4.13017	-2.59719
C	-5.04691	-1.61115	-1.44136
H	-5.50187	-1.46914	-2.42069
C	-3.82222	-1.78535	0.979524
H	-3.30564	-1.81951	1.945576
C	2.899772	0.474584	-1.34486
H	2.899089	0.611261	-2.44039
H	3.786194	1.023632	-0.98474
C	-4.23052	-0.61634	-0.9346
H	-4.03798	0.291189	-1.51758
C	6.927809	-1.41239	-0.60868
H	7.713344	-0.85499	-0.08844
C	4.544592	-1.66444	-1.17061
C	5.60066	-0.99248	-0.52371
H	5.376533	-0.1015	0.075276
C	-6.20677	-5.00831	-0.3488
H	-5.24343	-5.51586	-0.16953
H	-6.86067	-5.70584	-0.88454
H	-6.66944	-4.79603	0.629662
C	2.573422	-1.43732	0.601832
H	3.13193	-0.74453	1.255908
H	2.87809	-2.45057	0.918391
C	0.773339	-0.77326	2.756316
C	0.200004	-2.96282	1.134149
C	7.257568	-2.54439	-1.35749
H	8.296235	-2.87925	-1.43213
C	2.141425	2.167186	0.787953
C	1.231046	2.784595	-1.78232
C	-0.80639	-2.60088	-2.62868
C	0.427585	-0.39684	-3.79853
C	1.969079	-2.01093	-2.00267
H	1.814717	-3.03281	-1.61117
H	2.394046	-2.13407	-3.01469
C	-6.65963	-3.71348	-2.44866
H	-7.34543	-2.85235	-2.5172
H	-7.24139	-4.62246	-2.63901
H	-5.90837	-3.6164	-3.25108
B	2.986538	-1.15791	-0.99397
H	1.401242	-1.45156	3.353822
H	-0.25927	-0.80847	3.133521
H	1.148744	0.252365	2.874476
H	0.288217	-3.47174	0.165484
H	-0.86142	-2.95603	1.420454
H	0.77986	-3.52465	1.881759
H	2.997832	2.793308	0.495061
H	2.494859	1.417021	1.507481
H	1.393752	2.797186	1.284908
H	2.187948	3.310419	-1.92076
H	0.494904	3.482877	-1.36082
H	0.858871	2.450637	-2.76002
H	-0.42206	-3.18426	-3.47951
H	-1.78845	-2.17788	-2.88736
H	-0.94477	-3.26666	-1.76613
H	0.686241	-1.11086	-4.59527
H	1.193254	0.388807	-3.77452
H	-0.54123	0.070682	-4.02364
H	-1.37407	-1.01401	-0.36598
H	-1.15143	0.757409	-1.412
N	-1.23892	2.549868	1.633318
C	-0.78841	2.5784	2.892668
H	-0.88298	1.645316	3.454444
C	-1.16434	3.680441	0.919972
H	-1.56441	3.627034	-0.09718
C	-0.62309	4.852888	1.403933
H	-0.59903	5.724269	0.751451
C	-0.11352	4.896721	2.727961
C	-0.22929	3.697327	3.476338
H	0.116696	3.629436	4.506451
N	0.446708	6.017327	3.245648
C	0.565361	7.211429	2.4315
H	1.175202	7.029905	1.530735
H	1.053004	7.999641	3.015754
H	-0.42023	7.587051	2.109952
C	0.95556	6.013747	4.603207
H	1.76221	5.273236	4.736159
H	1.362939	7.003592	4.836926
H	0.161284	5.792586	5.335348
**92**			
**1*'_DMAP_adduct**			
Co	-0.8299	1.065785	0.30601
P	0.126773	-0.3978	1.64128
P	1.052488	2.147483	0.05858
P	-0.46476	-0.16624	-1.47495
Si	-2.20779	2.044926	1.593505

H	-2.59384	1.502182	2.939512
H	-3.46308	2.680365	1.098361
N	-4.57971	-3.29353	-0.85782
N	-5.09959	0.075623	1.616
C	-4.76205	-2.20075	-0.05193
C	3.872204	-2.3381	-1.50033
H	3.009126	-2.76577	-2.02031
C	-4.4742	-2.23446	1.329918
H	-4.11002	-3.13998	1.814599
C	5.116875	-2.94574	-1.6842
H	5.20432	-3.82006	-2.3369
C	-5.24355	-0.97095	-0.55077
H	-5.49269	-0.83938	-1.6035
C	-4.65786	-1.0869	2.090699
H	-4.42485	-1.116	3.162216
C	2.375998	1.142389	-0.68117
H	2.370314	1.330114	-1.76893
H	3.338834	1.541826	-0.32128
C	-5.37929	0.106891	0.313614
H	-5.73498	1.06515	-0.0829
C	6.105174	-1.32982	-0.21017
H	6.979337	-0.92026	0.305633
C	3.694569	-1.21286	-0.67906
C	4.85522	-0.73763	-0.03986
H	4.781522	0.134238	0.621387
C	-4.03144	-4.51005	-0.30532
H	-3.05305	-4.33466	0.17422
H	-3.87808	-5.2388	-1.11032
H	-4.69939	-4.97031	0.445228
C	1.843836	-0.79397	1.189797
H	2.506633	-0.20105	1.844611
H	2.033025	-1.84795	1.456938
C	0.183751	-0.04053	3.436549
C	-0.70056	-2.02622	1.680679
C	6.244689	-2.44269	-1.04048
H	7.222529	-2.91205	-1.18109
C	1.864834	2.851591	1.542805
C	0.974568	3.656446	-0.97358
C	-1.78744	-1.35023	-1.90542
C	-0.34139	0.728452	-3.06617
C	1.072171	-1.14028	-1.41916
H	0.806954	-2.16271	-1.09522
H	1.444464	-1.23944	-2.45377
C	-4.97843	-3.24248	-2.24544
H	-6.04798	-2.99265	-2.35662
H	-4.81772	-4.22478	-2.70591

H	-4.39304	-2.50394	-2.82012
B	2.23683	-0.50286	-0.40998
H	0.725291	-0.82903	3.980454
H	-0.83927	0.037574	3.832413
H	0.693807	0.918785	3.602993
H	-0.62162	-2.49981	0.693307
H	-1.7658	-1.88771	1.909798
H	-0.23678	-2.69208	2.423647
H	2.773808	3.404356	1.260835
H	2.151778	2.046211	2.23143
H	1.179527	3.530426	2.068937
H	1.980825	4.062738	-1.15674
H	0.36346	4.420423	-0.47283
H	0.498283	3.420158	-1.93381
H	-1.51998	-1.9572	-2.78441
H	-2.70971	-0.78613	-2.10571
H	-1.98965	-2.01211	-1.05311
H	-0.2177	0.025456	-3.9041
H	0.523589	1.40397	-3.05438
H	-1.24952	1.328613	-3.21712
H	-2.21025	0.457487	0.545564
H	-1.50309	2.100004	-0.53155
N	-1.57042	3.722861	2.399379
C	-1.08673	3.765294	3.648301
H	-1.18679	2.842948	4.225923
C	-1.48014	4.829031	1.647486
H	-1.89068	4.748605	0.637048
C	-0.90289	5.997396	2.093513
H	-0.86878	6.850189	1.417971
C	-0.36784	6.062252	3.406064
C	-0.48892	4.884455	4.186351
H	-0.11795	4.833256	5.208282
**73**			
**1*'**			
Co	-0.81009	1.071022	0.310597
P	0.14943	-0.39318	1.648256
P	1.075285	2.146709	0.060141
P	-0.455	-0.15035	-1.48274
Si	-2.1881	2.038637	1.598408
H	-2.59105	1.475498	2.936702
H	-3.47151	2.629393	1.107915
C	3.858076	-2.35889	-1.51272
H	2.988978	-2.77906	-2.02857
C	5.094842	-2.98191	-1.69715
H	5.169787	-3.86004	-2.34617
C	2.394117	1.138645	-0.68144

H	2.39208	1.332122	-1.76809
H	3.358108	1.530813	-0.31682
C	6.105951	-1.37247	-0.23142
H	6.986074	-0.9719	0.281205
C	3.695978	-1.22885	-0.6948
C	4.863548	-0.76467	-0.06042
H	4.801989	0.110093	0.598337
C	1.857017	-0.80662	1.179878
H	2.531375	-0.22813	1.835768
H	2.034636	-1.86518	1.43601
C	0.231474	-0.01828	3.439344
C	-0.69839	-2.01017	1.714106
C	6.230001	-2.49009	-1.05767
H	7.201635	-2.97208	-1.19818
C	1.882331	2.844171	1.549564
C	0.994327	3.66019	-0.9643
C	-1.78781	-1.32175	-1.919
C	-0.32228	0.757014	-3.06541
C	1.073427	-1.13442	-1.42664
H	0.799459	-2.15381	-1.10065
H	1.442357	-1.23878	-2.46184
B	2.245269	-0.50803	-0.41948
H	0.774822	-0.80591	3.982647
H	-0.78534	0.068242	3.849146
H	0.749542	0.938753	3.592108
H	-0.65611	-2.48832	0.727063
H	-1.75382	-1.86256	1.982861
H	-0.2197	-2.67998	2.44378
H	2.784922	3.409536	1.272337
H	2.179411	2.034598	2.228829
H	1.189487	3.509792	2.083493
H	2.00019	4.066646	-1.14891
H	0.386045	4.421414	-0.45563
H	0.51396	3.430437	-1.92398
H	-1.51887	-1.9288	-2.79691
H	-2.70831	-0.75886	-2.12971
H	-1.99048	-1.98749	-1.0699
H	-0.20739	0.058343	-3.908
H	0.550559	1.422206	-3.05002
H	-1.22384	1.36783	-3.21257
H	-2.19198	0.456559	0.54845
H	-1.48529	2.109696	-0.52189
N	-1.59664	3.72255	2.41056
C	-1.11632	3.772006	3.661405
H	-1.2135	2.851974	4.243139
C	-1.50505	4.825954	1.653037

H	-1.90832	4.739897	0.640131
C	-0.93213	5.997089	2.095764
H	-0.89631	6.846267	1.415758
C	-0.40043	6.069256	3.40982
C	-0.52289	4.894717	4.1957
H	-0.1545	4.848632	5.218796
N	0.18985	7.19296	3.884375
C	0.306031	8.364728	3.038375
H	0.907937	8.159505	2.137308
H	0.80164	9.166558	3.596753
H	-0.68119	8.737326	2.71912
C	0.731366	7.216618	5.228968
H	1.525445	6.463322	5.364052
H	1.167905	8.20243	5.423724
H	-0.04932	7.037703	5.986797
**19**			
**DMAP**			
N	0.125695	0.319135	-3.02556
C	1.120554	-0.21831	-2.32196
H	1.998549	-0.5437	-2.89336
C	-0.93567	0.712232	-2.32371
H	-1.7596	1.15589	-2.89597
C	-1.06215	0.600093	-0.94484
H	-1.97483	0.956628	-0.46773
C	-0.00801	0.029933	-0.19769
C	1.116598	-0.38803	-0.94304
H	1.984103	-0.84167	-0.46411
N	-0.07263	-0.10775	1.164253
C	-1.24971	0.330336	1.877528
H	-1.43179	1.412006	1.751028
H	-1.11726	0.137969	2.948987
H	-2.15768	-0.20611	1.550056
C	1.027529	-0.709	1.88086
H	1.965287	-0.1404	1.753301
H	0.794997	-0.72918	2.952401
H	1.215751	-1.74861	1.559448
**171**			
**3***			
Co	2.037304	11.78634	2.794972
Co	0.548652	8.912337	5.309232
P	1.292783	12.12558	0.670638
P	4.177748	11.67008	2.181529
P	2.199979	13.96713	3.220211
P	-0.50774	6.939944	4.489547
P	1.493196	8.321744	7.275651
Si	1.427027	10.47453	4.276977

N	-1.6953	8.410989	7.142642
N	-1.0657	9.29872	6.35719
C	3.296803	14.82454	2.05987
H	2.919575	15.84822	1.926672
H	4.274878	14.93621	2.557651
C	-2.09204	6.212578	8.507146
C	1.983974	13.65516	-0.01095
H	1.988773	13.59803	-1.10837
H	1.288601	14.47444	0.226924
C	4.196034	15.08273	-0.49017
C	4.501583	12.74978	0.755205
H	5.545327	13.09755	0.809832
H	4.451449	12.13795	-0.15902
C	-1.37766	6.180198	5.900289
H	-2.45309	6.235558	5.679304
H	-1.14041	5.109394	5.960473
C	0.455767	6.943692	7.860998
H	0.915886	6.020899	7.474689
H	0.54548	6.865483	8.955068
C	1.824733	10.80934	-0.54287
H	2.92027	10.78934	-0.44544
C	-1.89439	6.431316	9.882362
H	-1.06425	7.060332	10.21664
C	1.506191	11.12471	-2.00666
H	0.425343	11.24853	-2.17943
H	1.839807	10.29528	-2.64997
H	2.009028	12.03487	-2.35993
C	4.424097	14.65523	-1.81216
H	4.117251	13.64418	-2.10829
C	4.62728	16.38358	-0.18768
H	4.489094	16.7783	0.823973
C	-3.79139	5.066513	10.49291
H	-4.44416	4.628968	11.25298
C	-2.83349	8.95357	7.604864
H	-3.48297	8.383994	8.265268
C	-3.1779	5.383563	8.176167
H	-3.39223	5.164154	7.126851
C	-1.10837	13.39036	-0.28629
H	-1.03845	14.2607	0.385026
H	-2.17342	13.25832	-0.53512
H	-0.5762	13.64714	-1.21286
C	-1.89292	7.381266	3.254708
C	5.436983	16.7574	-2.43598
H	5.908786	17.40153	-3.18298
C	5.433479	12.24547	3.431321
H	5.233712	13.32943	3.427806
C	5.029181	15.46523	-2.77056
H	5.183986	15.08883	-3.78626
C	-1.39394	11.71683	1.584687
H	-1.03751	10.77744	2.027314
H	-2.44975	11.5771	1.302352
H	-1.35712	12.48213	2.374407
C	5.234385	17.21179	-1.13578
H	5.549856	18.22096	-0.85389
C	5.199519	11.73498	4.853061
H	4.137679	11.77386	5.133173
H	5.770154	12.34055	5.575646
H	5.535877	10.69478	4.966936
C	-2.72179	5.880937	10.86043
H	-2.52728	6.087888	11.91662
B	3.48788	14.0716	0.59238
C	0.513869	14.74333	3.00302
H	0.106341	14.14241	2.177888
C	6.899291	12.06243	3.029608
H	7.214166	11.01259	3.126713
H	7.545225	12.65677	3.695054
H	7.104454	12.38624	1.999799
C	4.641978	9.936228	1.67573
H	3.746663	9.633312	1.111121
C	5.839299	9.790368	0.733424
H	5.775517	10.46092	-0.13453
H	5.883558	8.75822	0.350334
H	6.79391	9.989906	1.239072
C	-0.40497	14.50795	4.203061
H	-0.35658	13.47076	4.56682
H	-1.44991	14.71397	3.922597
H	-0.15893	15.17491	5.042508
C	4.753875	8.983699	2.86604
H	5.696742	9.126965	3.415286
H	4.732469	7.936564	2.525256
H	3.927283	9.116383	3.578323
C	-1.27021	7.837544	1.929183
H	-2.04699	8.276987	1.281608
H	-0.80646	7.014406	1.368245
H	-0.50267	8.607878	2.094759
C	2.255975	13.75281	6.083372
H	1.257575	14.12223	6.354792
H	2.901065	13.87966	6.967017
H	2.171963	12.67598	5.885753
C	0.521104	5.54706	3.695675
C	1.667618	6.191404	2.900145
H	1.296573	6.846761	2.099175

H	2.287227	5.409235	2.430823
H	2.313274	6.799647	3.542401
C	-4.01321	4.81748	9.140942
H	-4.8437	4.176306	8.831855
C	2.848442	14.48901	4.885692
H	3.896657	14.15865	4.80098
C	0.465036	16.20406	2.549745
H	0.922899	16.89056	3.276056
H	-0.5859	16.51195	2.426752
H	0.963059	16.35836	1.582493
C	-0.56712	12.11471	0.362234
H	-0.69708	11.30776	-0.37586
C	1.092562	4.675608	4.828594
H	1.360014	5.25237	5.719071
H	1.996223	4.150832	4.478937
H	0.371056	3.910938	5.148956
C	-0.22358	4.576026	2.768412
H	-1.08133	4.101627	3.266332
H	0.471861	3.769445	2.481526
H	-0.57052	5.04313	1.837645
C	-2.69431	8.562318	3.824505
H	-2.09045	9.473155	3.89378
H	-3.11166	8.35435	4.820972
H	-3.53988	8.775258	3.150814
C	-1.79753	10.41865	6.335977
H	-1.46487	11.27895	5.757726
C	1.29373	9.427052	-0.16075
H	1.520214	9.169891	0.883878
H	1.739527	8.653066	-0.80481
H	0.201337	9.360004	-0.28643
C	-2.90984	6.255729	2.995115
H	-3.74405	6.664314	2.40207
H	-3.3422	5.863932	3.927243
H	-2.5002	5.412493	2.432416
C	2.871547	16.00296	5.107501
H	3.33352	16.54037	4.266982
H	3.451148	16.24212	6.013
H	1.859074	16.40681	5.257533
B	-1.14226	6.917293	7.361541
C	3.565879	6.947465	5.94063
H	2.955307	6.046572	5.846358
H	4.620408	6.627854	5.921399
H	3.39089	7.575629	5.060795
C	3.301448	7.71074	7.242961
C	-0.01565	9.79911	9.22222
H	-0.68504	10.27134	8.498525

H	0.030442	10.46681	10.09743
H	-0.46853	8.859662	9.558479
C	-2.94474	10.25012	7.113002
H	-3.73938	10.96756	7.298347
C	1.414257	9.61875	8.687241
C	4.271603	8.901214	7.213031
H	5.290489	8.531658	7.013136
H	4.313737	9.472	8.147311
H	4.010911	9.596666	6.401311
C	2.250586	9.226447	9.917579
H	1.927547	8.261238	10.3365
H	2.092532	9.98476	10.7012
H	3.329125	9.180749	9.734206
C	3.641018	6.742964	8.388959
H	2.998446	5.850716	8.364212
H	3.558997	7.184709	9.386435
H	4.680723	6.396118	8.269278
C	1.854208	10.98799	8.155963
H	2.875791	11.00273	7.757175
H	1.801729	11.73403	8.966146
H	1.177663	11.31294	7.354021
H	1.832138	8.74572	4.495403
H	0.642699	11.83204	3.430267
H	-0.04156	9.644311	4.068761
H	1.861224	10.264	2.612439
**175**			
**5***			
Co	8.664927	11.34253	5.859012
P	7.09703	9.737449	6.008289
P	9.144698	11.17641	8.041306
P	7.197343	13.04131	6.163793
Si	10.21796	11.03518	4.524633
C	11.85783	11.99294	8.092798
H	12.26644	11.15443	8.675696
H	12.55402	12.83715	8.211532
H	11.86371	11.70406	7.032497
C	7.688516	11.55128	9.056084
H	7.73992	10.95003	9.977471
H	7.768377	12.59686	9.391209
C	7.74176	7.989044	5.938282
H	8.598666	8.05535	6.62303
C	8.306967	7.614772	4.570935
H	7.511036	7.417091	3.837297
H	8.914434	6.699355	4.643924
H	8.951521	8.408771	4.167659
C	5.093958	11.3736	9.544638

C	6.826464	6.887113	6.479725
H	6.401443	7.136108	7.461363
H	7.404885	5.956761	6.597771
H	5.993129	6.664631	5.800193
C	6.173556	9.853066	7.568514
H	5.135192	9.548477	7.365984
H	6.574831	9.090273	8.256074
C	9.679444	9.495749	8.642976
H	8.736104	8.935118	8.530922
C	10.46114	12.38784	8.567549
H	10.17337	13.28462	7.998956
C	7.896133	14.55823	6.996471
H	8.134665	14.14752	7.991976
C	5.752864	9.878972	4.718639
H	5.288617	10.83316	5.019904
C	6.916175	15.71425	7.216694
H	6.739985	16.2722	6.285287
H	7.334942	16.42792	7.943716
H	5.942775	15.38419	7.604889
C	5.838611	12.54773	7.25714
H	5.459404	13.43765	7.780618
H	4.999412	12.21036	6.62654
C	6.419335	13.5895	4.563813
H	6.157681	12.61534	4.121294
C	4.658343	8.81016	4.779106
H	5.009613	7.856126	4.358865
H	3.795032	9.130286	4.174733
H	4.292763	8.620572	5.797245
C	3.244348	11.5457	11.70863
H	2.532904	11.61213	12.53629
C	4.022413	12.64844	11.3528
H	3.925158	13.58834	11.90453
C	10.07553	9.406146	10.11851
H	11.07188	9.838225	10.29489
H	10.12312	8.350168	10.42771
H	9.36149	9.910847	10.78393
C	7.417925	14.25239	3.614949
H	7.004745	14.29986	2.595541
H	8.367593	13.69906	3.566992
H	7.642731	15.28552	3.918755
C	9.200326	15.08533	6.391269
H	9.893724	14.27913	6.116699
H	9.714096	15.74369	7.109913
H	9.016288	15.67876	5.484404
C	10.72768	8.819403	7.758627
H	10.51156	8.975025	6.693194

H	10.75548	7.734975	7.951114
H	11.73771	9.209242	7.948216
C	4.300423	10.28383	9.935259
H	4.385014	9.332342	9.400374
C	3.389921	10.36061	10.99286
H	2.789216	9.485356	11.25836
C	6.236416	10.03852	3.276595
H	7.030118	10.78892	3.182993
H	5.399919	10.35173	2.631976
H	6.625642	9.095771	2.866222
C	4.922453	12.55339	10.29402
H	5.519449	13.43892	10.04272
C	5.123714	14.39783	4.663469
H	5.307825	15.43756	4.966646
H	4.409897	13.96249	5.376484
H	4.629972	14.43006	3.678955
C	10.47743	12.78208	10.04692
H	9.493747	13.11037	10.40805
H	11.17867	13.61905	10.19388
H	10.81183	11.96132	10.69561
B	6.203637	11.32576	8.332591
H	9.8612	12.29067	5.697348
H	8.490649	11.43971	4.336441
H	10.57879	12.29343	3.35746
H	11.94686	11.42844	4.714162
Co	11.77182	11.34182	3.190876
P	13.34083	9.738045	3.041381
P	11.29163	11.17552	1.009
P	13.23851	13.04155	2.88655
C	8.578514	11.99295	0.957488
H	8.169358	11.15475	0.374556
H	7.88275	12.83754	0.838952
H	8.572538	11.7039	2.01774
C	12.74771	11.55052	-0.00597
H	12.69672	10.94855	-0.92692
H	12.66709	12.59577	-0.34194
C	12.69714	7.989472	3.110881
H	11.83909	8.056163	2.427584
C	12.13513	7.613326	4.478925
H	12.93281	7.418633	5.211523
H	11.53089	6.695675	4.406777
H	11.48809	8.405041	4.882734
C	15.34222	11.37564	-0.49499
C	13.61229	6.888903	2.566472
H	14.03773	7.140816	1.585791
H	13.03367	5.959079	2.445257

H	14.44518	6.664091	3.245836
C	14.26464	9.854	1.481493
H	15.30338	9.550735	1.684231
H	13.8643	9.090521	0.794132
C	10.75746	9.494699	0.40724
H	11.70088	8.934289	0.519919
C	9.975378	12.38721	0.482635
H	10.26341	13.28383	1.051304
C	12.53889	14.55839	2.054327
H	12.30041	14.14786	1.058744
C	14.68461	9.879515	4.331466
H	15.15048	10.83255	4.029075
C	13.51862	15.71468	1.834367
H	13.69459	16.27247	2.765912
H	13.09972	16.42843	1.107496
H	14.49212	15.38498	1.446143
C	14.59723	12.54904	1.792669
H	14.97554	13.43926	1.269041
H	15.43696	12.21247	2.423017
C	14.0164	13.58952	4.486591
H	14.27868	12.6153	4.928636
C	15.7772	8.808683	4.272793
H	15.42417	7.855978	4.694794
H	16.641	9.128413	4.876656
H	16.14257	8.616576	3.255032
C	17.19016	11.54938	-2.66025
H	17.90105	11.61647	-3.48834
C	16.41233	12.65183	-2.30304
H	16.50886	13.59203	-2.85439
C	10.36242	9.404687	-1.06855
H	9.366035	9.836335	-1.24569
H	10.31548	8.348641	-1.3776
H	11.07673	9.909578	-1.73354
C	13.01725	14.2511	5.4358
H	13.43045	14.29855	6.455195
H	12.06814	13.69682	5.48357
H	12.79146	15.28413	5.132469
C	11.23468	15.08541	2.65959
H	10.54097	14.27931	2.933612
H	10.72125	15.74422	1.941113
H	11.4186	15.67836	3.566776
C	9.70901	8.818175	1.291178
H	9.924992	8.973564	2.356671
H	9.681138	7.7338	1.098403
H	8.699066	9.208169	1.101586
C	16.13613	10.28644	-0.88642

H	16.05209	9.334619	-0.35204
C	17.04569	10.36397	-1.94478
H	17.64694	9.489235	-2.21075
C	14.20156	10.04129	5.773431
H	13.41045	10.79433	5.867096
H	15.03919	10.35203	6.417777
H	13.80946	9.099946	6.184249
C	15.51279	12.55586	-1.24392
H	14.91601	13.4412	-0.99147
C	15.31132	14.39897	4.387307
H	15.12606	15.43898	4.085805
H	16.02509	13.96535	3.673174
H	15.80566	14.43019	5.371551
C	9.959183	12.7817	-0.99666
H	10.94302	13.10946	-1.35789
H	9.258457	13.61915	-1.1434
H	9.624165	11.96131	-1.64547
B	14.2329	11.32666	0.717312
**175**			
**7***			
Co	23.9324	4.785285	3.138637
Fe	27.98859	4.579067	2.454737
P	29.22888	3.019927	3.441697
P	29.15339	6.349919	3.142074
P	22.65492	6.493621	2.428111
P	22.62877	3.281286	2.111983
Si	31.50653	4.614696	2.103586
P	28.9159	4.44178	0.427349
P	22.83115	4.577103	5.113999
B	20.4445	4.788956	3.416683
C	33.37936	4.640016	1.960555
C	30.72285	4.824285	0.419167
H	30.85406	5.880343	0.146279
H	31.25202	4.238623	-0.34584
C	18.80585	4.706415	3.547106
C	20.92603	3.340444	2.74272
H	20.23815	3.066717	1.926759
H	20.81026	2.542866	3.493647
C	23.20852	1.527942	2.383616
H	23.48026	1.5592	3.449053
C	21.07345	5.001929	4.946533
H	20.49563	4.448232	5.701977
H	20.95276	6.063466	5.219632
C	23.51032	5.701119	6.438213
H	23.64732	6.633727	5.867829
C	30.97139	6.020743	3.223199

H	31.19845	5.694186	4.250607
H	31.58058	6.920466	3.053465
C	22.59382	6.027248	7.619728
H	22.47034	5.171959	8.298298
H	21.59185	6.343808	7.298044
H	23.02711	6.851059	8.210201
C	28.24863	5.606888	-0.87353
H	28.11462	6.516075	-0.2684
C	20.88767	6.051182	2.43306
H	20.3106	6.952099	2.693646
H	20.60488	5.813056	1.393328
C	16.52606	5.369487	2.918312
H	15.87085	6.009673	2.3194
C	22.78294	2.854743	5.83793
H	22.17663	2.338854	5.074244
C	28.95553	2.742055	-0.3638
H	29.62095	2.20935	0.339185
C	29.51031	3.191863	5.287743
H	30.09026	4.131251	5.314523
C	36.19669	4.698348	1.835818
H	37.28839	4.719536	1.786434
C	22.9665	7.100523	0.692251
H	22.9003	6.150892	0.141448
C	28.85048	6.854905	4.923751
H	28.95809	5.891856	5.44477
C	34.16345	4.413832	3.104089
H	33.6795	4.211037	4.06602
C	29.64316	0.125815	3.374358
H	30.60674	0.294713	2.873205
H	29.2234	-0.80676	2.963728
H	29.84837	-0.05579	4.437813
C	21.58571	2.403381	-0.44061
H	21.29213	2.754857	-1.44276
H	20.66465	2.134567	0.095523
H	22.17903	1.487404	-0.58096
C	15.97606	4.432485	3.7891
H	14.89145	4.329719	3.884323
C	30.97233	2.974495	2.842837
H	31.04346	2.239499	2.025335
H	31.67581	2.63164	3.615021
C	34.04629	4.89909	0.754681
H	33.47173	5.083081	-0.15796
C	28.64855	1.268428	3.159234
H	28.42112	1.301183	2.082357
C	29.13624	5.973387	-2.06467
H	29.25796	5.140283	-2.77008

H	30.14129	6.306538	-1.76903
H	28.67281	6.804007	-2.62094
C	22.39179	3.490208	0.272826
H	21.77721	4.4058	0.251752
C	27.63242	8.368391	1.90116
H	27.29605	7.720838	1.079993
H	27.56368	9.415682	1.565195
H	26.91188	8.225777	2.715827
C	28.22936	3.43026	6.092653
H	27.55405	4.13713	5.595273
H	28.47367	3.829388	7.090086
H	27.66736	2.49813	6.245451
C	17.91338	5.496437	2.805208
H	18.30453	6.243805	2.107105
C	35.55504	4.441086	3.047678
H	36.14289	4.260041	3.95123
C	21.81585	9.172055	3.169906
H	22.27292	9.773582	2.37086
H	21.69137	9.835662	4.040402
H	20.81143	8.874479	2.840031
C	23.68604	3.789371	-0.4801
H	24.29097	4.527184	0.062572
H	23.47193	4.186075	-1.48597
H	24.30475	2.889392	-0.60807
C	27.33141	0.992596	3.885841
H	27.49244	0.818891	4.960471
H	26.85331	0.087186	3.48146
H	26.62128	1.82511	3.776398
C	24.37963	7.634912	0.474875
H	24.52033	8.622283	0.941108
H	24.58441	7.75423	-0.6014
H	25.13969	6.954644	0.88718
C	22.0506	2.69265	7.172202
H	21.84802	1.626487	7.363316
H	21.08672	3.21996	7.198399
H	22.66168	3.058492	8.01124
C	22.1693	0.415713	2.219818
H	21.23476	0.626615	2.756887
H	22.57558	-0.52695	2.621594
H	21.91455	0.234937	1.166824
C	26.85574	5.187356	-1.33473
H	26.34577	6.024826	-1.8357
H	26.23296	4.880457	-0.48428
H	26.89582	4.354063	-2.05272
C	29.06723	8.031749	2.308943
H	29.37227	8.735335	3.100234

C	24.10973	8.489547	3.890806
H	24.56828	9.016019	3.040423
H	24.78375	7.674053	4.177644
H	24.06473	9.203684	4.72874
C	27.43213	7.351507	5.193464
H	27.27493	8.367667	4.800825
H	27.24606	7.393711	6.277854
H	26.67349	6.691553	4.7529
C	35.43992	4.928794	0.689052
H	35.93675	5.131435	-0.26326
C	24.14133	2.149273	5.893006
H	24.72974	2.46354	6.767017
H	24.74813	2.354993	5.001174
H	24.00417	1.05849	5.971314
C	29.58177	2.632927	-1.75673
H	29.77439	1.575472	-1.99782
H	30.53732	3.167082	-1.85497
H	28.89971	3.018088	-2.52915
C	29.86702	7.8271	5.526571
H	30.90714	7.490059	5.420631
H	29.67039	7.946453	6.603871
H	29.79321	8.830622	5.079419
C	27.61238	2.009465	-0.3471
H	26.93567	2.377913	-1.13125
H	27.0947	2.126741	0.611778
H	27.7645	0.933151	-0.52681
C	24.89649	5.266864	6.913423
H	25.39866	6.091288	7.444172
H	25.53895	4.966539	6.072696
H	24.83619	4.421057	7.615002

C	21.92034	8.037235	0.081734
H	20.89092	7.72307	0.303209
H	22.03276	8.053317	-1.01451
H	22.03851	9.071633	0.431598
C	30.36073	2.121884	5.976856
H	29.78663	1.197609	6.13736
H	30.67636	2.479781	6.969946
H	31.27165	1.857857	5.420692
C	30.01276	8.266793	1.129418
H	29.73304	7.666983	0.252699
H	31.06626	8.058393	1.364444
H	29.95655	9.321745	0.817701
C	22.7064	7.986034	3.553088
H	22.28189	7.551994	4.473731
C	24.48504	1.198659	1.612212
H	24.28835	1.051248	0.539568
H	24.92809	0.263592	1.989218
H	25.23861	1.991783	1.71567
C	16.83265	3.625164	4.53899
H	16.42048	2.881321	5.22789
C	18.21269	3.766589	4.412127
H	18.85583	3.114786	5.016182
Si	25.93676	4.860607	2.524471
H	26.94086	3.473895	2.049788
H	27.14027	4.709455	3.769726
H	24.93356	3.688317	3.46608
H	24.93452	5.754374	3.75588

REFERENCES

(1) Handford, R. C.; Smith, P. W.; Tilley, T. D. Activations of All Bonds to Silicon (Si–H, Si–C) in a Silane with Extrusion of [CoSiCo] Silicide Cores. *J. Am. Chem. Soc.* **2019**, *141*, 8769–8772.

(2) Handford, R. C.; Nguyen, T.; Teat, S. J.; Britt, R. D.; Tilley, T. D. Direct Transformation of SiH_4 to a Molecular $(H)_2Co{=}Si{=}Co(H)_2$ Silicide Complex. *Submitted* **2022**.

(3) Neumeyer, F.; Lipschutz, M. I.; Tilley, T. D. Group 8 Transition Metal Complexes of the Tripodal Triphosphino Ligands $PhSi(CH_2PR_2)_3$ (R = Ph, *i*Pr): Group 8 Transition Metal Complexes of Triphosphino Ligands. *Eur. J. Inorg. Chem.* **2013**, *2013*, 6075–6078.

(4) Fulmer, G. R.; Miller, A. J. M.; Sherden, N. H.; Gottlieb, H. E.; Nudelman, A.; Stoltz, B. M.; Bercaw, J. E.; Goldberg, K. I. NMR Chemical Shifts of Trace Impurities: Common Laboratory Solvents, Organics, and Gases in Deuterated Solvents Relevant to the Organometallic Chemist. *Organometallics* **2010**, *29*, 2176–2179.

(5) Mestrelab Research: Mnova. https://mestrelab.com/software/mnova/.

(6) Ammann, C.; Meier, P.; Merbach, A. E. A Simple Multinuclear NMR Thermometer. *J. Magn. Reson.* **1982**, *46*, 319–321.

(7) Sandström, J. *Dynamic NMR Spectroscopy*; Academic Press: London, 1982.

(8) Sheldrick, G. M. *SHELXT*– Integrated Space-Group and Crystal-Structure Determination. *Acta Crystallogr.* **2015**, *A71*, 3–8.

(9) Sheldrick, G. M. Crystal Structure Refinement with SHELXL. *Acta Cryst* **2015**, *C71*, 3–8.

(10) Dolomanov, O. V.; Bourhis, L. J.; Gildea, R. J.; Howard, J. A. K.; Puschmann, H. OLEX2: A Complete Structure Solution, Refinement and Analysis Program. *J Appl Cryst* **2009**, *42*, 339–341.

(11) Kratzert, D.; Krossing, I. Recent Improvements in *DSR*. *J. Appl. Crystallogr.* **2018**, *51*, 928–934.

(12) Neese, F.; Wennmohs, F.; Becker, U.; Riplinger, C. The ORCA Quantum Chemistry Program Package. *J. Chem. Phys.* **2020**, *152*, 224108.

(13) Chemcraft - Graphical Software for Visualization of Quantum Chemistry Computations. Https://Www.Chemcraftprog.Com.

(14) Wiberg, K. B. Application of the Pople-Santry-Segal CNDO Method to the Cyclopropylcarbinyl and Cyclobutyl Cation and to Bicyclobutane. *Tetrahedron* **1968**, *24*, 1083–1096.

(15) Bader, R. F. W. Atoms in Molecules. *Acc. Chem. Res.* **1985**, *18*, 9–15.

(16) Lu, T.; Chen, F. Multiwfn: A Multifunctional Wavefunction Analyzer. *J. Comput. Chem.* **2012**, *33*, 580–592.

(17) Lin, Y.-S.; Li, G.-D.; Mao, S.-P.; Chai, J.-D. Long-Range Corrected Hybrid Density Functionals with Improved Dispersion Corrections. *J. Chem. Theory Comput.* **2013**, *9*, 263–272.

(18) Weigend, F.; Ahlrichs, R. Balanced Basis Sets of Split Valence, Triple Zeta Valence and Quadruple Zeta Valence Quality for H to Rn: Design and Assessment of Accuracy. *Phys. Chem. Chem. Phys.* **2005**, *7*, 3297–3305.

(19) Reed, A. E.; Weinstock, R. B.; Weinhold, F. Natural Population Analysis. *J. Chem. Phys.* **1985**, *83*, 735–746.

(20) Glendening, E. D.; Landis, C. R.; Weinhold, F. *NBO 6.0* : Natural Bond Orbital Analysis Program. *J. Comput. Chem.* **2013**, *34*, 1429–1437.

(21) Henkelman, G.; Uberuaga, B. P.; Jónsson, H. A Climbing Image Nudged Elastic Band Method for Finding Saddle Points and Minimum Energy Paths. *J. Chem. Phys.* **2000**, *113*, 9901–9904.

(22) Barone, V.; Cossi, M. Quantum Calculation of Molecular Energies and Energy Gradients in Solution by a Conductor Solvent Model. *J. Phys. Chem. A* **1998**, *102*, 1995–2001.

(23) Kelly, C. P.; Cramer, C. J.; Truhlar, D. G. Single-Ion Solvation Free Energies and the Normal Hydrogen Electrode Potential in Methanol, Acetonitrile, and Dimethyl Sulfoxide. *J. Phys. Chem. B* **2007**, *111*, 408–422.
